# De Novo Gene Transcription of Connexin Mediates Cytoplasmic Fluid Exchange and Flocking Transitions in Physiological and Cancerous Epithelial Systems

**DOI:** 10.1002/advs.202508648

**Published:** 2025-12-23

**Authors:** Hind Abdo, Leonardo Barzaghi, Yuan Shen, Edoardo Bellini, Emanuele Martini, Serena Magni, Sara Barozzi, Fabrizio Orsenigo, Dario Parazzoli, Galina V. Beznoussenko, Jasmin Di Franco, Fabian Krautgasser, Jasmin Kaivola, Mario Cinquanta, Alessandro Lazzarin, Sara Sigismund, Johanna Ivaska, Roberto Cerbino, Giorgio Scita

**Affiliations:** ^1^ IFOM the FIRC Institute of Molecular Oncology Milan 20139 Italy; ^2^ Department of Oncology and Haemato‐Oncology University of Milan Milan 20122 Italy; ^3^ Universite Paris Cite CNRS Institut Jacques Monod Paris F‐75013 France; ^4^ Faculty of Physics University of Vienna Vienna 1010 Austria; ^5^ Vienna Doctoral School in Physics University of Vienna Vienna 1010 Austria; ^6^ Turku Bioscience Center University of Turku and Åbo Akademi University Turku FI‐20520 Finland; ^7^ Cogentech Società Benefit srl via Adamello 16 Milan 20139 Italy; ^8^ IEO European Institute of Oncology IRCCS Milan 20141 Italy; ^9^ Department of Life Technologies University of Turku Turku 20520 Finland; ^10^ InFLAMES Research Flagship Center University of Turku Turku 20521 Finland; ^11^ Foundation for the Finnish Cancer Institute Helsinki 00130 Finland

**Keywords:** collective motility, connexins and fluid exchange, EGFR signaling, jamming transition, tissue‐level phase transition

## Abstract

The initial invasion of tumors requires a transition from a solid, jammed state to a fluid‐like, flocking, unjammed state that enables collective migration. Here, we show that de novo gene transcription is essential for the emergence of flocking in epithelial tissues and identify connexins (Cx) as key mediators of this transition. Using quiescent HaCaT keratinocytes, tumorigenic A431 epidermoid carcinoma cells, primary bronchial epithelial explants, and vocal fold carcinoma (VFC) cells, we find that flocking induction depends on transcriptional programs activated downstream of epidermal growth factor (EGF). EGF stimulation upregulates Cx26 and Cx31 and enhances gap‐junctional intercellular communication (GJIC), which is necessary—though not sufficient—to generate the large‐scale cell‐volume fluctuations and density heterogeneity that accompany unjamming. Sustained signaling through extracellular signal‐regulated kinase 1/2 (ERK1/2) and AKT serine/threonine kinase (AKT) downstream of the EGF receptor (EGFR) is required for connexin induction, linking mechanical state transitions to extracellular cues. Pharmacological inhibition and CRISPR‐Cas9 (clustered regularly interspaced short palindromic repeats–CRISPR associated protein 9) knockout of connexins block unjamming and collective motility. VFC cells display constitutively elevated connexins and persistent flocking that is highly sensitive to connexin inhibition. Consistently, high Cx26 expression correlates with reduced survival across carcinomas. These findings reveal a transcriptionally controlled, connexin‐dependent mechanism that enables tissue fluidization and collective invasion.

## Graphical abstract

EGF‐induced de novo transcription of connexins Cx26 and Cx31 promotes flocking behavior that fluidizes epithelia and enables coordinated collective migration. Connexin‐driven cytoplasmic exchange mechanistically links growth‐factor signaling to invasive dynamics. These results uncover how flocking underpins early cancer dissemination and position connexins as essential regulators and promising therapeutic targets.

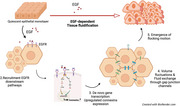

## 1 Introduction

The first steps of invasion of a tumor in its immediate surroundings are critical for its dissemination and for the development of metastasis. In the early stage of tumor development, malignant carcinomas frequently evolve within the confined boundary of epithelial tissues, where they can display highly cohesive growth associated with extreme cell packing and density. This condition exerts compressive stress and favors a transition to a solid, jammed state that prevents cell motility and exerts tumor suppressive function.^[^ ^1^ ^]^ To become malignant and disseminate, cancerous cells need to acquire a certain level of fluidity, undergoing a solid‐to‐liquid‐like or jammed‐to‐unjammed phase transition (PT).^[^ ^2^, ^3^, ^4^ ^]^ A remarkable form of unjamming PT can occur through the emergence of flocking fluid mode of motion, which describes the shift from a densely packed, static arrangement of cells to a fluid‐like, dynamic state characterized by highly coordinated, collective movement and local fluctuations in cell velocity akin to the movement observed in flocks of birds or herds of animals and is essential for the dissemination of solid tumors.^[^ ^1^, ^5^ ^]^

Solid‐to‐liquid PT is influenced by multiple factors, including cell number, density, and shape, all of which arise from the complex interplay of cell–cell adhesion, cell‐substrate interaction, cellular activity, stiffness, and cell–cell communication. In the context of cancer progression or during wound repair and tissue regeneration, understanding how these factors influence cell behavior in a dense environment and facilitate their migration is crucial.^[^ ^6^, ^7^ ^]^

Cells that move collectively within tissues by undergoing a flocking fluid PT represent a type of active matter, where collective motion is heavily influenced by fluctuations at the single‐cell level.^[^ ^4^, ^8^ ^]^ Although fluctuations in cell area and density are commonly observed in monolayers undergoing a flocking transition, their specific role in facilitating collective migration remains unclear,^[^ ^9^, ^10^ ^]^ limiting our understanding of how single‐cell dynamics influence collective cell motion. In Madin‐Darby canine kidney (MDCK) cell monolayers, for example, it was shown that cell volumes can fluctuate by 20%, oscillating on a timescale of ≈4 h. Furthermore, estimates of cell permeability indicated that these volume fluctuations may facilitate fluid transport between cells.^[^ ^9^, ^10^ ^]^ These results suggest that fluid transport associated with cell volume fluctuations may be sufficient to overcome the energy barrier needed for a tissue to become “liquid” and contribute to the onset of collective motion in monolayers and tissues both in physiological and pathological conditions. However, whether these alterations are causative of unjamming via flocking PT has remained unclear, and even less explored are the molecular determinants that control cell volume fluctuation and intercellular fluid transport.

Transfer of fluids and cytoplasmic material among neighbouring cells can be controlled by Gap junctional intercellular communication (GJIC). Connexins are the main components of Gap junctions (GJ) and comprise 21 isoforms in human cells, expressed in a cell‐and tissue‐specific manner.^[^ ^11^, ^12^ ^]^ Connexins and GJ are required in broad physiological functions such as cell growth, cell differentiation, and tissue homeostasis,^[^ ^11^ ^]^ but they are also involved in several pathological conditions such as wound repair, inflammation, and cancer progression.^[^ ^13^, ^14^, ^15^, ^16^ ^]^ Connexins and Gap junctions have been implicated as modulators of cell migration in various contexts, such as development and wound healing.^[^ ^15^, ^17^, ^18^, ^19^, ^20^, ^21^ ^]^ Connexins function as important components of GJ channels or hemichannels, but they can also trigger different downstream signaling pathways.^[^ ^22^, ^23^, ^24^ ^]^ Although initially considered as tumors suppressors,^[^ ^25^ ^]^ increasing evidence portrays GJ and connexins as the main players in cancer progression.^[^ ^13^, ^16^, ^26^, ^27^, ^28^ ^]^ Furthermore, Connexins have been recently involved in promoting breast cancer metastasis progression via FAK activation^[^ ^23^ ^]^ and Connexins 43 and 26 have been shown to be instrumental for the seeding of breast cancer metastatic noduli into the brain.^[^ ^27^ ^]^

Here, we hypothesize that interactions among neighboring cells in dense collectives, mediated by GJ, enable fluid exchange that drives large and frequent fluctuations in cell area and density. We posit that these fluctuations generate critical perturbations that are required for unjamming the otherwise solid, immobile cell population, triggering collective motility in the form of a highly coordinated, flocking movement.

To explore this hypothesis, we use a model of quiescent, jammed epithelial keratinocyte cells that undergo a robust phase‐transition and migrate collectively upon EGF stimulation.^[^ ^29^ ^]^ This model of mitogen‐induced reawakening of quiescent epithelial monolayers recapitulates key aspects of physiological responses to wounding but also of early neoplastic transformation, including the activation of ERK signaling, mechanical tension buildup, and coordinated motility, as previously reported in keratinocyte systems and other epithelial contexts.^[^ ^29^, ^30^, ^31^ ^]^ Furthermore, this paradigm holds significant pathophysiological relevance for understanding cancer progression, particularly concerning quiescent cancerous cells. Epidermal Growth Factor (EGF) stimulation can mimic critical events in tumor development by reactivating motility programs in dormant or quiescent cancer cells, which are often resistant to therapy and contribute to recurrence. This includes promoting Epithelial‐Mesenchymal Transition (EMT), a crucial process for cancer cell invasion and metastasis, and directly enhancing the coordinated motility and invasiveness of these cells.^[^ ^32^, ^33^ ^]^

Consistently, we further extend our investigation to explants from healthy individuals of primary bronchial epithelial cells and to epithelial squamous carcinoma and vocal fold cancers. Notably, Vocal Fold Cancer (VFC) typically originates in the epithelial layer of the vocal cords, specifically from keratinocytes, the primary cell type in this region. As a squamous cell carcinoma, VFC arises when the keratinocytes within the pseudo‐stratified epithelium undergo malignant transformation, often driven by factors like smoking, alcohol use, and human papillomavirus (HPV) infection.^[^ ^34^, ^35^ ^]^ VFC is a unique malignancy arising within the mechanically active environment of the vocal folds. These structures rely on a well‐organized extracellular matrix (ECM) to maintain the viscoelasticity required for regular vocal fold vibration and function. In VFC, however, ECM composition is significantly altered, with increased ECM deposition and tissue stiffening that disrupts normal tissue mechanics. This enhanced rigidity impacts their physical state and progression by triggering collective dynamic movements and facilitating mechanotransduction pathways that respond to tissue stiffness, contributing to aggressive tumor behaviors.^[^ ^34^, ^35^ ^]^

Here, we hypothesize that fluid exchange‐mediated fluctuations, regulated downstream of EGFR signaling, are critical to trigger flocking‐like collective behavior in epithelial monolayers. We aim to uncover whether such transcriptionally regulated, connexin‐mediated perturbations are sufficient to promote or sustain this transition.

Our findings reveal a novel role for connexins in promoting unjamming and collective flocking motility. While endocytosis and sustained EGFR‐dependent ERK1/2 signaling are already established requirements for unjamming, our data now show that de novo transcriptional upregulation of connexins is also critical. We identified connexins 26 and 31 as key gene products that drive intercellular fluid exchange, as their depletion disrupts EGF‐induced flocking. Moreover, aggressive vocal fold cancer (VFC) cells exhibit heightened, constitutive connexin expression even in the absence of EGF and enhanced and sustained EGF‐dependent flocking behavior. Pharmacological inhibition of connexins in these cancer cells blocks both their constant flocking and their enhanced active wetting on solid substrates, a behavior mimicking VFC collective spreading and dissemination. Together, our results emphasize the relevance of these findings for understanding coordinated motility in epithelial monolayers; however, we acknowledge that these in vitro models may not fully recapitulate the complexity of carcinoma behavior in vivo.

## 2 Results

### 2.1 EGF Stimulation Promotes Flocking Fluid Transition of Epithelial Ensembles

**Figure 1:**
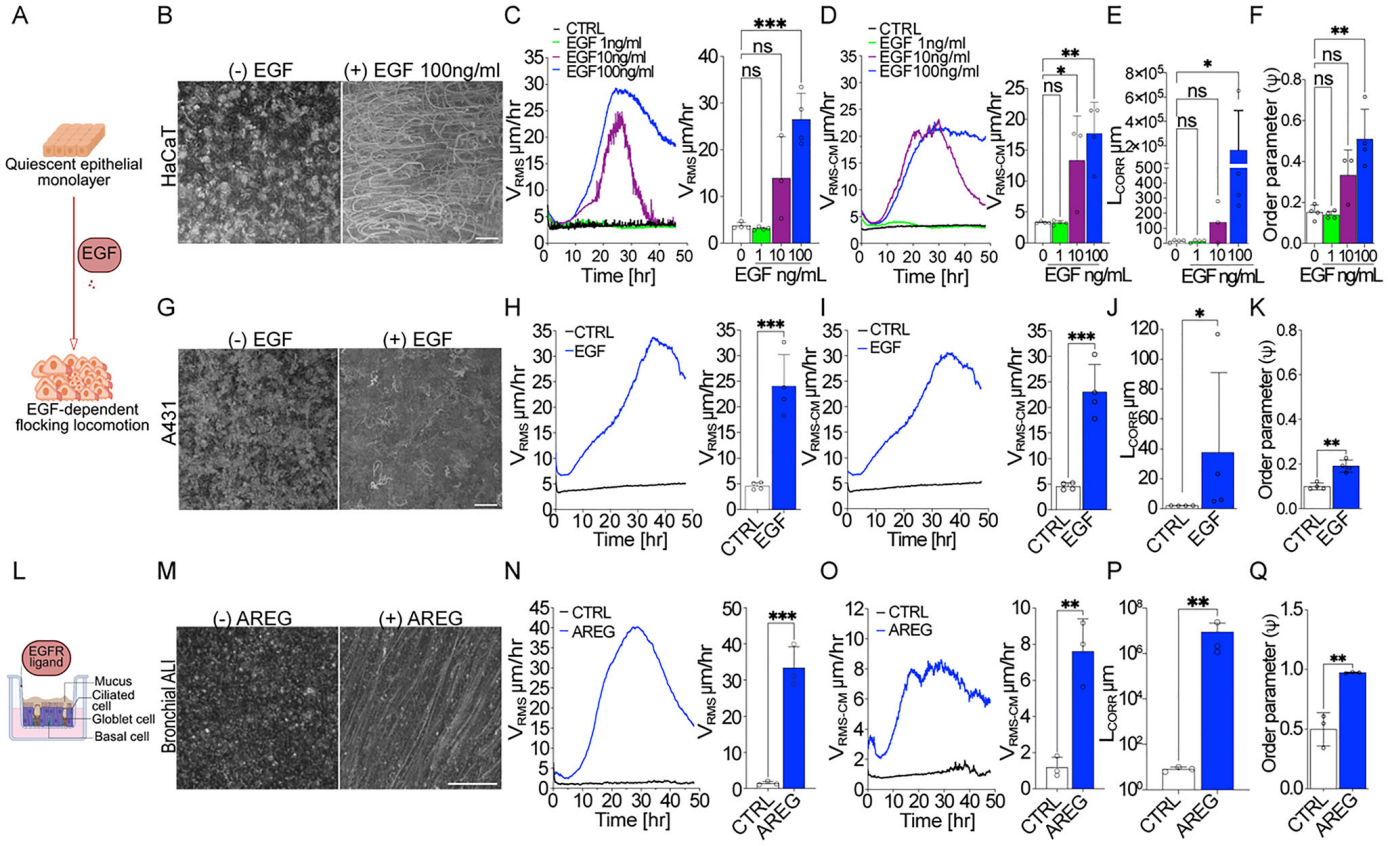
EGF stimulation promotes flocking locomotion in several epithelial ensembles. A) Schematic drawing illustrating experimental model of EGF‐induced tissue fluidification (Biorender). B–F) Collective motility induced in HaCaT quiescent monolayer seeded on tissue culture plastic without additional ECM coating (panels A‐B) upon EGF treatment: Dose response (1, 10, and 100 ng mL^−1^). B) Representative phase contrast images of the maximum intensity projection (MIP) of all frames acquired over a 24‐h period (5 min frame^−1^) showing collective dynamics in starved and EGF‐stimulated HaCaT monolayer. Representative phase contrast image representative images from *n* = 4 time‐lapse series. C) Quantification of total cell motility (V_RMS_) over time (left) and of its mean measured in the 15–40 h framecut (right). D) Quantification of local cell velocity fluctuations measured as the Mean square Velocity of the Center of Mass (V_RMS‐CM_) over time (left) and of its mean in the framecut 15–40 h (right). E) Length of Correlation on 15–40 h period. F) Order parameter (ψ) on 15–40 h period. V_RMS_, V_RMS‐CM_, L_CORR,_ and Order Parameter are the mean ± SD (*n* = 4 independent experiments). G–K) Collective motility induced in A‐431 cells upon 100 ng mL^−1^ of EGF treatment. G) Representative phase contrast image of the maximum intensity projection (MIP) of all frames acquired over a 24‐h period (5min frame^−1^). Representative images from *n* = 4 time‐lapse series H) Quantification of total cell motility (V_RMS_) over time (left) and of its mean in the 10–40 h framecut (right). I) Quantification of local cell velocity fluctuations measured as the Mean square Velocity of the Center of Mass (V_RMS‐CM_) over time (left) and of its mean measured in the framecut 10–40 h (right). J) Length of Correlation on 15–40 h time period. K) Order parameter (ψ) on 15–40 h period. V_RMS_, V_RMS‐CM_, L_CORR,_ and Order Parameter are the mean ± SD (*n* = 4 independent experiments). L) Schematic drawing illustrating experimental model of primary cultures of bronchial epithelial cells in Air‐Liquid‐Interface (ALI) (Biorender). M–R) Collective motility induced in Bronchial epithelial primary cultures in Air‐Liquid interface (ALI) upon treatment with 100 ng mL^−1^ Amphiregulin (AREG). M) Representative phase contrast image of the maximum intensity projection (MIP) of all frames acquired over a 24‐h period (5min frame^−1^). Representative images from *n* = 3 time‐lapse series. N) Quantification of total cell motility (V_RMS_) over time (left) and of its mean in the framecut 10–40 h (right). O) Quantification of local cell velocity fluctuations as the Mean square Velocity of Center of Mass (V_RMS‐CM_) over time (left) and of its mean in the framecut 10–40 h (right). P) Length of Correlation on 15–40 h time period. Q) Order parameter (ψ) on 15–40 h period. V_RMS_, V_RMS‐CM_, L_CORR,_ and Order Parameter are the mean ± SD (*n* = 3 independent experiments). Scale bars, 100 µm. Statistical tests and significance are indicated in **Table** **1**.

As an experimental model of unjamming via flocking transition, we employed Human keratinocyte HaCaT cells, confluent and serum‐starved to induce a quiescent, immobile state, then treated with EGF before recording cell motion (**Figure** **1**A). Please note that in the absence of EGF, but in the presence of serum no monolayers are fully jammed and kinetically arrested (Figure S1A, Supporting Information), as previously reported.^[^ ^29^ ^]^ This quiescent‐reawakening protocol leads to the emergence of flocking through a striking reawakening of long‐range collective motility^[^ ^29^ ^]^ (Figure 1A). This system further enables us to quantify several locomotory parameters using particle image velocimetry (PIV), cell tracking, and hydrodynamics (flow and forces) measurements.^[^ ^3^ ^]^ In addition, we genetically engineered HaCaT cells with Fluorescent Ubiquitination‐Based Cell Cycle Indicator (FUCCI), EGFP‐E‐Cadherin, or H2B‐mCherry constructs to monitor cell cycle progression, area, volume, and density fluctuations in real‐time.^[^ ^36^ ^]^

**Table 1:** Statistical tests performed and significance. The following table indicates for each of the experiments in the various figures the statistical tests that have been used to evaluate significance.

Figure	panel	n	test	significance	significance	significance	Additional information
**Figure 1**							
	1C	4	One‐way ANOVA, Benferroni Post Hoc test	ns (CTRLvsEGF1ng/ml)	ns (CTRLvsEGF10ng/ml)	***p = 0,0001 (CTRLvsEGF100ng/ml)	framecut15‐40h
	1D	4	One‐way ANOVA, Benferroni Post Hoc test	ns (CTRLvsEGF1ng/ml)	*p = 0,024 (CTRLvsEGF10ng/ml)	**p = 0,0012 (CTRLvsEGF100ng/ml)	framecut15‐40h
	1E	4	Kruskal‐Wallis, Dunn's Post Hoc test	ns (CTRLvsEGF1ng/ml)	ns (CTRLvsEGF10ng/ml)	*p = 0,0342(CTRLvsEGF100ng/ml)	framecut15‐40h
	1F	4	One‐way ANOVA, Benferroni Post Hoc test	ns (CTRLvsEGF1ng/ml)	ns (CTRLvsEGF10ng/ml)	**p = 0,0014(CTRLvsEGF100ng/ml)	framecut15‐40h
	1H	4	unpaired t‐test	***p = 0,008			framecut15‐40h
	1I	4	unpaired t‐test	***p = 0,005			framecut15‐40h
	1J	4	Mann‐whitney test	* *p* = 0,0286			framecut15‐40h
	1K	4	unpaired t‐test	**p = 0,0011			framecut15‐40h
	1N	3	unpaired t‐test	***p = 0,0007			framecut15‐40h
	1O	3	unpaired t‐test	***p = 0,0041			framecut15‐40h
	1P	3	Ratio paired t test	**p = 0.0045			framecut15‐40h
	1Q	3	unpaired t‐test	**p = 0,0041			framecut15‐40h
**Figure 2**							
	2A	5	unpaired t‐test	*p = 0,0249(EGFvsEGF + Dyn)			framecut15‐40h
	2B	4	unpaired t‐test	**p = 0,0053 (RTN3KDvsNEG)	ns− *p* = 0.8871 (RTN4KDvsNEG)		framecut15‐40h
	2C	4	unpaired t‐test	ns− *p* = 0.651 (EGF vs CLTHCKD)	ns− *p* = 0.7386 (NEG vs CLTHCKD)		framecut20‐40h
	2I	4	unpaired t‐test	****p < 0.0001 (EGF + PD vs EGF)	ns− *p* = 0.716 (EGF + PD vs CTRL)		framecut15‐40h
	2K	6	unpaired t‐test	**p = 0.0082 (EGF + MK2206 vs EGF)	ns− *p* = 0.1581 (EGF + MK2206 vs CTRL)		framecut20‐40h
	2M	4	unpaired t‐test	ns− *p* = 0.0884	***p = 0,0009		framecut20‐40h
**Figure 3**							
	3B	6	unpaired t‐test	**p = 0,0094 (EGF + AD vs EGF)			framecut15‐40h
	3C	6	Ratio paired t‐test	**p = 0,0011 (EGF vs EGF + DRB)			framecut 20‐40h
**Figure**	**panel**	**n**	**test**	**significance**	**significance**	**significance**	**Additional information**
**Figure 4**							
	4C	3	One‐way ANOVA, Benferroni Post Hoc test	****p < 0.0001 (0h vs 30h)	****p < 0.0001 (6h vs 30h)	ns− *p* = >0.99999	n=38‐48 fields of view analyzed from 3 independent experiments
	4E	3	One‐way ANOVA, Benferroni Post Hoc test	****p < 0.0001 (0h vs 30h)	****p < 0.0001 (6h vs 30h)	ns− *p* = >0.99999	n=38‐48 fields of view analyzed from 3 independent experiments
	4H	3	unpaired t‐test	***p = 0,0009			framescut 12h
	4I	3	unpaired t‐test	*p = 0,0337			
	4J	3	unpaired t‐test	ns− *p* = 0,0573 (30h vs 0h)			
	4K	3	unpaired t‐test	*p = 0.0360 (30h vs 0h)			
**Figure**	**panel**	**n**	**test**	**significance**	**significance**	**significance**	**Additional information**
**Figure 5**							
	5B	5	One‐way ANOVA, Benferroni Post Hoc test	**p = 0,0054 (CTRL vs EGF)	*p = 0,0148 (EGF + CBX vs EGF)		framescut 15‐40h
	5C	3	One‐way ANOVA, Benferroni Post Hoc test	**p = 0,0012 (CTRL vs EGF)	**p = 0,0054 (CTRL vs EGF)		framescut 15‐40h
	5D	3	paired t‐test	*p = 0,0274 (AREG + CBX vs AREG)			framescut applied to each experiment
	5E	3	One‐way ANOVA, Benferroni Post Hoc test	****p < 0.0001 (CTRL vs EGF)	****p < 0.0001 (EGF + CBX vs EGF)	ns− *p* = 0.984 (EGF + CBX vs CTRL)	n=38‐48 fields of view analyzed from 3 independent experiments
	5F	3	One‐way ANOVA, Benferroni Post Hoc test	****p < 0.0001 (CTRL vs EGF)	****p < 0.0001 (EGF + CBX vs EGF)	ns− p>0.99 (EGF + CBX vs CTRL)	n=36‐47 fields of view analyzed from 3 independent experiments
	5H	3	unpaired t‐test	**p = 0,0091			
	5J	n=4‐6	unpaired t‐test	***p = 0,0006			
	5L	3	Two‐way ANOVA, Benferroni Post Hoc test	*p = 0,0329 (t24h, EGF‐CBX vs EGF)	**p = 0,0076(t30h, EGF‐CBX vs EGF)		
	5N	n=3‐6	One‐way ANOVA, Benferroni Post Hoc test	**p = 0,0024(Cx26KO vs WT)	***p = 0,0008 (Cx31KO vs WT)	**p = 0,0057 (Cx26/31KO vs WT)	
**Figure 6**							
	6C	n=5‐6	unpaired t‐test	****p < 0.0001 (HaCaT, CTRL vs EGF)	**p = 0,0044 (T1, CTRL VS EGF)	*p = 0,0199 (T3, CTRL VS EGF)	framescut 0‐48h
	6D	3	One‐way ANOVA, Benferroni Post Hoc test	ns− p>0.99 (HaCaT vs T1)	ns− *p* = 0.5962 (HACAT vs T3)	ns− *p* = 0.1615 (T1 vs T3)	
	6E		One‐way ANOVA, Benferroni Post Hoc test	ns− p>0.99 (HaCaT vs T1)	*‐*p* = 0,0394 (HACAT vs T3)	ns− *p* = 0,0788 (T1 vs T3)	framescut 15‐40h
	6K	n=4‐6	One‐way ANOVA, Benferroni Post Hoc test	****p < 0.0001 (CTRL vs EGF)	***p = 0.0002 (EGF + CBX vs EGF)	ns− p>0.999 (CTRL vs EGF + CBX)	framescut10–48h
	6L	n=3‐6	One‐way ANOVA, Benferroni Post Hoc test	****p < 0.0001 (CTRL vs EGF)	*p = 0.0131 (EGF + CBX vs EGF)	**p = 0.0033 (CTRL vs EGF + CBX)	framescut10–48h
	6N	n=3‐4	Two‐way ANOVA, Benferroni Post Hoc test	****p < 0.0001 (30h, EGF vs EGF + CBX)			
	6P	n=3‐4	Two‐way ANOVA, Benferroni Post Hoc test	****p < 0.0001 (30h, EGF vs EGF + CBX)			
**Supplementary Figure 1**							
	1A	3	One‐way ANOVA, Benferroni Post Hoc test	ns− p>0.99 (Starved vs NonStarved)	**p = 0.0066 (NonStarved vs EGF)	**p = 0.0060 (Starved vs EGF)	
	1E	4	unpaired t‐test	*p = 0,0463 (EGF + Y27632 vs EGF)			framecut10‐40h
	1G	4	One‐way ANOVA, Benferroni Post Hoc test	*p = 0,0475 (CTRL vs EGF + Y27632)	ns− *p* = 0.0574 (EGF vs EGF + Y27632)		
	1I	3	unpaired t‐test	ns− *p* = 0.6792			framecut10‐40h
	1K	3	One‐way ANOVA, Benferroni Post Hoc test	p**=0,0044 (EGF vs CTRL)	**p = 0,0086 (EGF vs EGF‐PALB)	ns‐p>0.999 (EGF + PALB vs CTRL)	
**Supplementary Figure 4**							
	4B	4	One‐way ANOVA, Benferroni Post Hoc test	*p = 0,0475 (CTRL vs EGF + Y27632)	**‐ *p* = 0,0034(CTR vsEGF)	**‐ *p* = 0,0010 (EGF vs EGF + VA)	framecut24‐36h

PIV analysis revealed a substantial increase in cell motility upon EGF treatment, as quantified by the root mean square velocity (V_RMS_), which rose in a dose‐dependent manner (Figure 1B,C; Movie S1, Supporting Information). Notably, this increase in motility and the emergence of coordinated, collective movement were observed only at high, saturating doses of EGF (>10 ng mL^−1^). In contrast, a low EGF dose (1 ng mL^−1^) did not reawaken cell motility, although it was sufficient to induce cell cycle entry (see below) and activate canonical EGFR signaling pathways (Figure S1B, Supporting Information). The difference was stark, with mean velocities reaching 26.49 ± 5.63 µm h^−1^ at 100 ng mL^−1^ EGF, compared to only 3.12 ± 0.325 µm h^−1^ at 1 ng mL^−1^.

Further analysis of the motility of the center of mass of each individual cell (V_RMS‐CM_) provided insights into local fluctuations in cell velocity (Figure 1D). Both total V_RMS_ and V_RMS‐CM_ significantly increased with high doses of EGF, with V_RMS‐CM_ reaching an average velocity of 17.7 ± 5.04 µm h^−1^ (Figure 1D).

To assess the degree of coordinated movement, we calculated the velocity correlation length (L_CORR_), which quantifies the spatial scale over which velocity vectors of neighboring cells remain aligned, and showed a remarkable 10^4^‐fold increase in the presence of EGF, reflecting a millimeter‐scale coordination of cell migration (Figure 1E). We further determined the order parameter (ψ = |⟨v⟩|/⟨|v|⟩, which also increases significantly after EGF stimulation (Figure 1F; Movie S1, Supporting Information). This parameter can vary from 0 to 1, quantifies the uniformity of the velocity field v, with ψ = 1 indicating perfectly coordinated motion where all cells move in the same direction and at the same speed, while ψ ≃ 0 for randomly oriented velocities.

This increase in L_CORR_ and order parameter underscores the extensive, collective alignment of cells under high‐dose EGF stimulation, revealing the critical role of EGF in triggering flocking motion in epithelial cell populations. The collective alignment of cells refers, here, to their coordinated directional movement, which we quantified using the spatial correlation length (L_CORR_) derived from V_PIV (V_RMS_) velocity fields and the order parameter ψ. While L_CORR_ and the order parameter capture the scale of collective kinematic coordination, additional shape metrics such as aspect ratio and shape index were used elsewhere to further assess cellular morphology and orientation. To ensure consistent interpretation of the observed collective behaviors, we classified tissue states according to a combination of physical metrics. A jammed solid was defined by V_RMS_ ≈0, low L_CORR_, and minimal shape deformation or neighbor exchange. An unjammed, disordered fluid exhibited elevated V_RMS_ but low L_CORR_. A flocking fluid combined high V_RMS_ with extended L_CORR_, indicating directional coordination. Finally, a flocking solid maintained alignment (high L_CORR_) but exhibited moderate neighbor exchange (please see also Experimental Section for additional details).

To further investigate how cells respond to EGF, we monitored the cell cycle status of migrating cells using the FUCCI construct, which is used to monitor cell cycle dynamics via expression of Cdt1‐mCherry and Geminin‐GFP.^[^ ^36^ ^]^ Several reports have shown that one mechanism driving tissue fluidization is through increased cell division.^[^ ^37^, ^38^, ^39^ ^]^ This tool allows us to visualize different stages of the cell cycle, with cells in G2/M phases marked by green fluorescent nuclei. After treating HaCaT‐FUCCI cells with EGF at various concentrations (1–100 ng mL^−1^), we observed that EGF stimulation led to re‐entry into the cell cycle, indicated by an increased proportion of cells with green nuclei (Figure S1C,D and Movie S2, Supporting Information). Interestingly, unlike cell motility, cell cycle re‐entry occurred even at low doses of EGF, suggesting that cell proliferation and motility are regulated independently and further indicating that cell division is insufficient to drive the onset of flocking motility in this system. To confirm this independence, we used pharmacological inhibitors to selectively disrupt cell motility and cell cycle progression. First, we applied the Rho kinase inhibitor, Y27632, which effectively blocked cell motility without impacting EGF‐induced cell cycle re‐entry (Figure S1E–H and Movie S3, Supporting Information). Conversely, we treated HaCaT cells with Palbociclib, a CDK4/6 inhibitor that arrests the cell cycle in the G1 phase. Despite this block in cell proliferation, Palbociclib did not alter the motility response to EGF, reinforcing the notion that EGF‐induced cell motility does not depend on cell cycle entry or proliferation (Figure S1I–L and Movie S4, Supporting Information).

These findings highlight that EGF independently regulates cell motility and cell cycle progression, with each pathway responding to different thresholds of EGF signaling and likely governed by distinct signaling responses.

In addition to HaCaT cells, we used the A431 cell line as a tumorigenic model to study EGF‐dependent cell motility, as these cells are characterized by particularly high levels of EGFR expression.^[^ ^40^ ^]^ In this model, EGF treatment induced a significant increase in cell motility (Figure 1G,H; Movie S5, Supporting Information). The total cell velocity in A431 cells reached 24.06 ± 6.17 µm h^−1^, a level similar to that observed in HaCaT cells. Interestingly, this increase in A431 motility was almost entirely attributed to uncoordinated cell motion, measured by plotting the velocity of the center of mass at 23.03 ± 5.35 µm h^−1^ (Figure 1I), indicating a high degree of local fluctuations in cell velocity. This is further reflected in the velocity correlation length (L_CORR_) and the order parameter (Ψ), which increased by only ≈20‐ and 2‐fold, respectively in A431 cells, compared to a dramatic 1.63 × 10^4^‐fold increase and 4‐fold in HaCaT cells (Figure 1J,K). This suggests that, while A431 cells exhibit robust motility and fluidization in response to EGF stimulation, presumably due to their high EGFR expression, they display a less coordinated, flocking behavior as compared to that seen in HaCaT cells.

To expand our investigation to a more complex, physiologically relevant system, we utilized the air‐liquid interface (ALI) culture model, which allows us to study the primary explant of airway epithelial tissues (Figure 1L). These ALI cultures, derived from bronchial epithelium, closely mimic in vivo airway structure and function.^[^ ^41^, ^42^, ^43^, ^44^, ^45^, ^46^ ^]^ Studies have shown that these primary cultures can undergo unjamming transitions under various conditions, such as during embryonic development, after irradiation or compressive stress, and in disease contexts, like asthma and idiopathic pulmonary fibrosis.^[^ ^41^, ^42^, ^43^, ^45^, ^46^ ^]^ Notably, recent research indicates that the activation of the ERBB family of EGFR receptors is both necessary and sufficient to induce fluidization in distal airway epithelia, highlighting the potential role of EGFR signaling in promoting cellular fluidity within complex epithelial tissues.^[^ ^46^ ^]^

We used ALI cultures of primary explants derived from the brush biopsies of proximal bronchial airways of healthy donors. When these ALI cultures were treated with EGF (Figure S1M and Movie S6, Supporting Information) or Amphiregulin, ligands of the EGFR, they exhibited robust and sustained flocking motion (Figure 1L–Q Movie S7, Supporting Information). Amphiregulin, like EGF, activates the EGFR signaling cascade, albeit with reduced binding affinity and slower receptor internalization kinetics, which may explain its more modest effects on collective migration compared to EGF.^[^ ^47^ ^]^ Notably, however, prolonged exposure to EGF but not Amphiregulin, as previously reported,^[^ ^46^ ^]^ compromised epithelial barrier integrity of this culture. We therefore focused further analysis on the Amphiregulin.

The collective movement induced by Amphiregulin was marked by a substantial increase in total cell velocity, reaching 33.36 ± 65.79 µm h^−1^. However, the intrinsic velocity of the center of mass (V_RMS‐CM_) was moderately elevated to 7.6 ± 1.8 µm h^−1^, suggesting a less fluid but more stable collective motion consistent with the emergence of a flocking‐solid mode of motion previously predicted by biophysical modeling but lacking definitive experimental validation^[^ ^48^ ^]^ (Figure 1N,O). This flocking behavior in ALI cultures was also characterized by a high level of coordination among cells, as evidenced by the correlation length, which increased by 10^6^‐fold—higher than the 10^4^‐fold increase in HaCaT cells and 40‐fold in A431 cells and the order parameter ψ = 0.97, indicating almost perfect coordination and alignment of the collective motility (Figure 1P–Q)This indicates that while ALI cultures exhibit somewhat reduced local cell rearrangement, their cell movements are highly coordinated over larger distances.

Collectively, these findings suggest that, across various epithelial models with distinct levels of complexity, EGFR stimulation consistently induces a flocking motion phenotype. However, the specific characteristics of this collective behavior vary, reflecting differences in the fluidity and coordination levels among cell types.

### 2.2 EGF‐Mediated Flocking Requires Endocytosis and Activation of ERK and AKT Downstream Pathways

Next, we sought to identify the downstream signaling pathways and processes activated by EGFR that contribute to the initiation and maintenance of this flocking motion.

To this end, we initially examined the role of endocytosis in EGF‐induced cell motility. Endocytosis is essential for EGFR signaling, as it enables receptor internalization and subsequent modulation of downstream EGFR‐dependent pathways. It is also required for RAB5A‐induced flocking‐fluid motion.^[^ ^3^, ^49^ ^]^ Importantly, EGFR can be internalized via two main routes: clathrin‐dependent and non‐clathrin‐dependent endocytosis (NCE), with the chosen route significantly influenced by the dose of EGF.^[^ ^50^ ^]^

**Figure 2:**
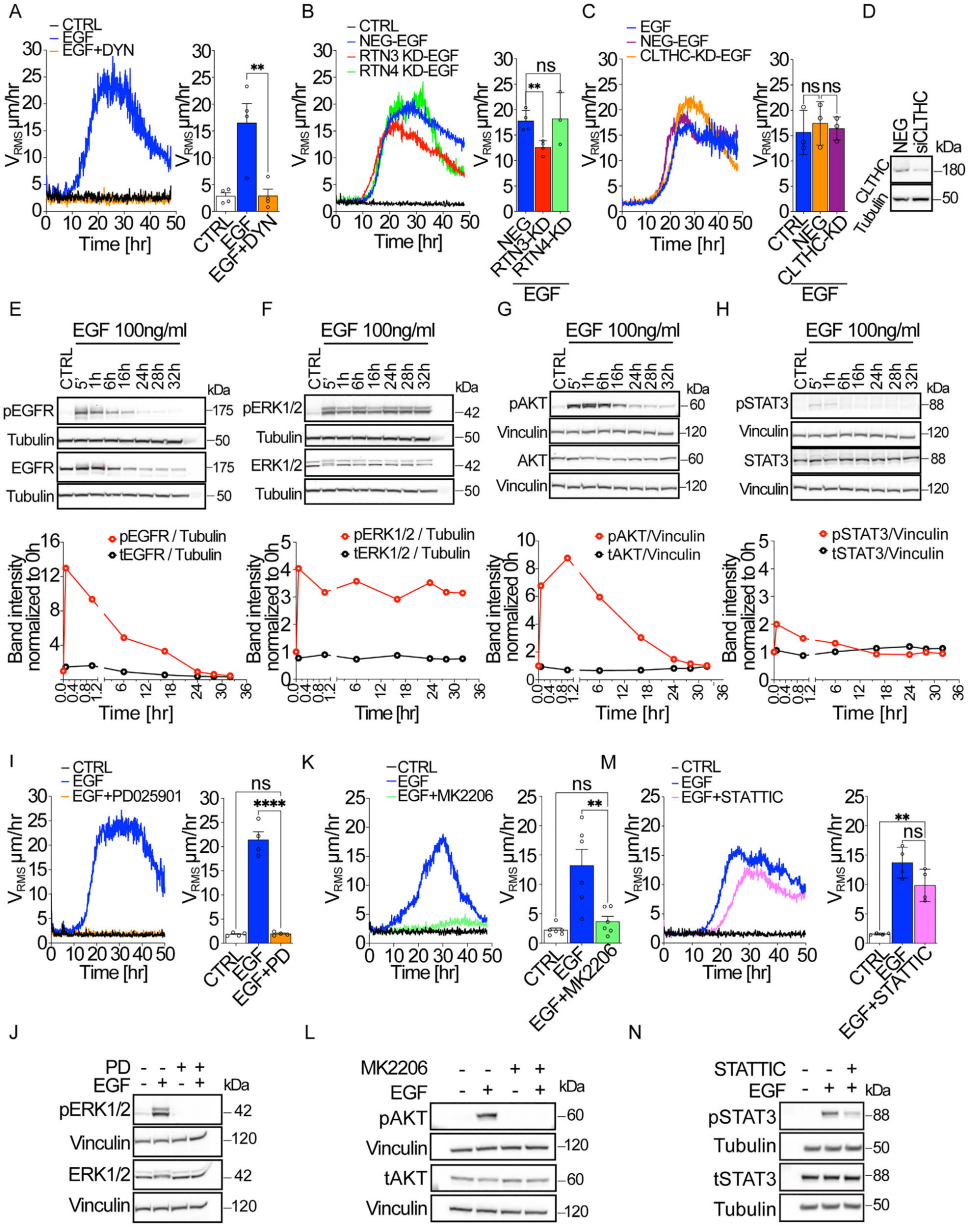
EGF‐mediated flocking requires endocytosis proficiency and activation of MAPK and AKT downstream pathways. A–C) Time evolution of the Root Mean Square Velocity (V_RMS_) over 48 h (left) and its mean measured in the 10–40 h framecut (right) in the absence (CTRL) of the presence of an inhibitor of Dynamin (Dynasore) (A) or after silencing of RTN3 or RTN4 (B) or Clathrin Heavy Chain (CLTHC) (C). The efficacy of RTN3 and RTN4 silencing was measured by qRT‐PCR (mRNA fold increase relative to the levels of control cells after normalizing for GAPDH and 18S mRNA levels) (Figure S2, Supporting Information). D) The efficacy of CLTHC silencing in HaCaT cells was analyzed by WB. V_RMS_ is expressed as the mean ± SD (*n* = 4 independent experiments). NEG‐EGF, refers to monolayers treated with scrambled oligos and EGF (Negative Control+EGF). E–H) Expression and phosphorylation status of EGFR (E) and the following signaling effectors: ERK1/2 (F), AKT (G), and STAT3 (H) in HaCaT cells, stimulated with EGF (100 ng mL^−1^), collected at different timepoints, and analyzed by WB. Tubulin and Vinculin are used as loading controls. Molecular weights are indicated on the side. The representive time evolution of bands intensity normalized with respect to the loading control is shown on the bottom (*n*= 3 independent experiments). I–N) Role of MAPK (I,J), AKT (K,L) and STAT3 (M,N) pathways in EGF‐induced flocking motion in HaCaT cells. (I,K,M) Root Mean Square Velocity (V_RMS_) measured by Particle velocimetry analysis (PIV) over 48 h (left) and its mean within the 15–40 h framecut (right) in the absence or the presence of the inhibitor of ERK1/2 (PD025901, 1 µm) (I), AKT (MK2206, 10 µm) (K), or STAT3 (STATTIC, 5 µm) (L). V_RMS_ is expressed as the mean ± SD (*n* = 4–6 independent experiments). (J,L,N) The effective inhibition of ERK1/2 (J), AKT (L), and STAT3 (N) phosphorylation in HaCaT cells, as compared to mock‐treated control, were analyzed by WB. Statistical tests and significance are indicated in Table 1.

We found that EGF‐induced collective motility is strictly dependent on high doses of EGF, under conditions that trigger robust NCE.^[^ ^51^ ^]^ Pharmacological inhibition of Dynamin, a critical mediator of both endocytotic pathways, completely blocked EGF‐induced flocking motion in HaCaT monolayers, indicating that endocytosis is crucial for this process (**Figure** **2**A; Movie S8, Supporting Information). Notably, dose‐response experiments confirmed that only high doses of EGF triggered collective motility (Figure 1C), suggesting that NCE is required for the cellular behaviors associated with flocking.

To further explore this, we performed knockdowns of specific genes involved in NCE and clathrin‐mediated pathways.^[^ ^50^ ^]^ Knockdown of Reticulon 3 (RTN3), a specific regulator of NCE that is driven by an increase in contact site between the plasma membrane and the Endoplasmic Reticulum (ER) induced by EGF stimulation, significantly reduced the V_RMS_ of flocking cells as a function of time. The reduction in V_RMS_ velocity over time reflects a dampened dynamic response of the monolayer to EGF stimulation, indicating that genetic inhibition of NCE interferes with the emergence of coordinated motility. Conversely, knockdown of Reticulon 4 (RTN4), an analogous ER resident protein but not essential for NCE, did not affect motility (Figure 2B; Figure S2 and Movie S9, Supporting Information). On the other hand, impairing clathrin‐dependent endocytosis through knockdown of Clathrin Heavy Chain had no impact on EGF‐induced collective motility (Figure 2C,D; Movie S10, Supporting Information). Interestingly, the reduction in flocking motility following RTN3 silencing suggests that either the silencing effect was temporary, or alternative clathrin‐independent internalization pathways are involved.

These findings underscore that the route of EGFR internalization is dose‐dependent, with high‐dose EGF favoring NCE to promote the initiation of flocking motion.

Next, we explored EGFR downstream signaling pathways, focusing on MAPK (ERK1/2) and AKT, both of which are required for the onset and maintenance of collective motility,^[^ ^50^ ^]^ and STAT3. Analysis of protein phosphorylation following EGF treatment revealed a robust and rapid activation of EGFR, ERK1/2, AKT, and STAT3 (Figure 2E–H). However, ERK1/2 showed sustained phosphorylation over time that persists even after 32 h, AKT and EGFR display elevated levels of phosphorylation even after 18 h from EGF stimulation, while STAT3 activation rapidly declined after 1 h. Interestingly, ERK1/2 displayed a prolonged activation pattern, with a secondary phosphorylation wave that continued beyond 32 h (Figure 2F).

To dissect the signaling pathways mediating EGF‐induced motility, we employed pathway‐specific inhibitors targeting MEK (PD025901) and AKT (MK‐2206), which are critical effectors of the two major arms of EGFR signaling—the MAPK and AKT cascades, respectively.^[^ ^52^ ^]^ Using these inhibitors, we confirmed that the activation of ERK1/2 and AKT pathways is essential for EGF‐induced flocking motion, while STAT3 signaling, which was inhibited using STATTIC, played only a minor role (Figure 2I–N; Movies S11–S13, Supporting Information). Together, these findings indicate that EGF‐induced collective motility is dependent on high‐dose EGFR signaling through NCE and requires sustained ERK1/2 and AKT activation.

### 2.3 EGF‐Mediated Flocking Motion Requires De Novo Transcription and is Associated with Upregulation of the Expression of Connexins mRNA

Given that flocking motion began several hours after EGF treatment, we hypothesized that this delay may be due to the upregulation of de novo gene transcription—a step that might underpin the sustained motility response to EGF. The potential role of transcriptional activation further highlights the complexity of signaling and molecular events driving the transition from a jammed, static state to a dynamic, coordinated cell migration.

**Figure 3:**
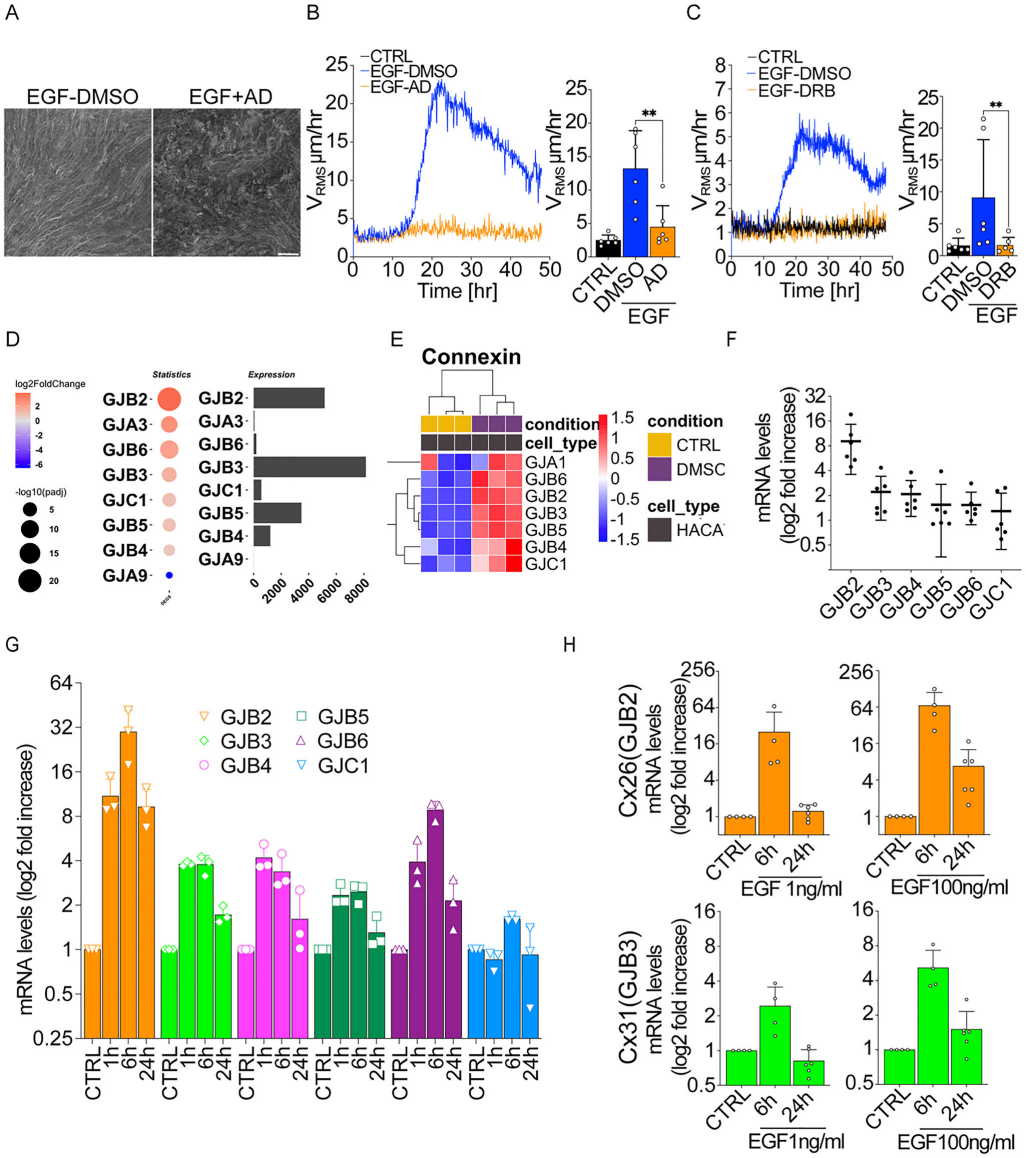
De novo transcription is required for the emergence of flocking motion. A–C) Emergence of Flocking motility of HaCaT cells upon EGF treatment in the absence or presence of Actinomycin D (AD) (A,B) or 5, 6‐dichloro‐1‐β‐D‐ribofuranosylbenzimidazole (DRB) (C); DMSO was used as vehicle control for the pharmacological treatment. A) Representative phase contrast image of the maximum intensity projection (MIP) of all frames acquired over a 24‐h period (5 min frame^−1^). Representative images from *n* = 6 time‐lapse series. Scale bar 100 µM. (B,C) Root Mean Square Velocity (V_RMS_) measured by Particle velocimetry analysis (PIV) over 48 h (left) and its mean within the 10–40 h framecut (right). V_RMS_ is expressed as the mean ± SD (*n* = 6 independent experiments). D,E) RNAseq analysis of HaCaT cells serum starved for 48h then treated or not with EGF (100ng mL^−1^) for 24 h (*n* = 3 independent experiments). D) Differential expression analysis of connexin genes. A comparison of the statistical significance and expression levels of different connexin genes. The left panel (Statistics) displays a bubble plot representing the significance of differentially expressed genes (DEGs), where the size of the dots corresponds to the ‐log10 (adjusted *p*‐value), and the color represents the log2 fold change (red indicates upregulation, blue indicates downregulation). The right panel (Expression) shows the absolute expression levels of the same genes as bar plots. Genes such as GJB2 and GJB3 exhibit high differential gene expression. E) Hierarchical clustering heatmap of connexin gene expression. The heatmap represents the expression levels of deregulated connexin genes (GJA1, GJB6, GJB2, GJB3, GJB5, GJB4, GJC1) in control quiescent vs EGF‐treated (for 24 h) HaCat cells. The rows correspond to genes, while the columns represent individual samples. Expression levels are scaled and color‐coded, with red indicating upregulation and blue indicating downregulation. Hierarchical clustering was applied to group genes with similar expression patterns. The top annotation bar denotes the experimental condition (CTRL: yellow, EGF: purple), and the cell type (HaCaT: black). The color scale represents the standardized expression values. F) mRNA levels of Connexins genes in HaCaT cells treated with EGF for 24 h, quantified by qRT‐PCR. Data are the mRNA fold increase relative to the levels of control cells after normalizing for GAPDH and 18S mRNA levels (*n* = 6 independent experiments). G) mRNA levels of Connexins genes in HaCaT cells treated with EGF for 0, 1, 6, and 24 h; quantified by qRT‐PCR. Data is the mRNA fold increase relative to the levels of control cells after normalizing for GAPDH and 18S mRNA levels) (*n* = 3 independent experiments). H) mRNA levels of Connexin 26 (GJB2) and Connexin 31 (GJB3) treated with low (1 ng mL^−1^) or high (100 ng mL^−1^) doses of EGF for 6 and 24 h. Data are mRNA fold increase relative to the levels of control cells after normalizing for GAPDH and 18S mRNA levels (*n* = 4–6 independent experiments). Statistical tests and significance are indicated in Table 1.

To test this hypothesis, we inhibited transcription pharmacologically using two distinct inhibitors, Actinomycin D (AD) and 5,6‐dichloro‐1‐β‐D‐ribofuranosylbenzimidazole (DRB), two mechanistically distinct inhibitors. Actinomycin D intercalates into DNA to prevent transcription elongation, while DRB induces the proteasomal degradation of RNA polymerase II. The consistent suppression of EGF‐induced motility by both compounds confirms the requirement of active transcription independently of the inhibitory mechanism employed.^[^ ^53^, ^54^ ^]^ Treatment with either inhibitor completely blocked EGF‐induced collective motility in the monolayer (**Figure** **3**A–C; Movies S14 and S15, Supporting Information). These results indicate that the onset of flocking motion upon EGF stimulation requires de novo gene transcription, as both DRB and Actinomycin D effectively suppressed these responses.

To identify specifi genes involved, we conducted RNA‐seq analysis on HaCaT cell monolayers that were confluent, starved, and then treated with EGF for 24 h. As expected, EGF‐treated cells displayed a wide range of deregulated genes (Figure S3A, Supporting Information). Among these, we observed significant upregulation of mRNA levels for certain connexins, such as Cx26 (GJB2), Cx31 (GJB3), and Cx30 (GJB6), which we subsequently validated by quantitative qRT‐PCR (Figure 3D–F; Figure S3B, Supporting Information). Notably, the ectopic expression of Cx26 (GJB2) has been reported to promote cell coupling and enhances wound migration in Hela cells.^[^ ^19^ ^]^ Importantly, here and throughout this manuscript, the term “de novo gene transcription” refers to a transcriptional response that is either newly induced or strongly upregulated following EGF stimulation. This includes transcripts present at very low or near‐undetectable baseline levels that become clearly detectable post‐stimulation. Specifically, for Connexins, this term denotes a strong and significant upregulation following EGF stimulation. Our operational definition captures EGF‐dependent activation of transcription, as measured through differential RNA‐seq expression (logFC, adjusted p‐value) and validated by qRT‐PCR.

Further examination of connexin mRNA levels at various time points showed peak expression at 6 h post‐EGF treatment, preceding the initiation of flocking motion. These levels slightly declined but remained elevated at 24 h, coinciding with the active phase of cell motility (Figure 3G). By contrast, a low dose of EGF (1 ng mL^−1^) also briefly increased connexin mRNA levels, but this transcriptional upregulation was short‐lived, with levels dropping between 6 and 24 h post‐treatment (Figure 3H; Figure S3C, Supporting Information). Furthermore, treatment of HaCaT cells with 100 ng mL^−1^ EGF in the presence of MEK (targeting ERK1/2 pathway) or AKT‐specific inhibitors prevented the upregulation of the mRNA of these connexins (Figure S3D,E, Supporting Information). Importantly, the upregulation of connexin mRNA was not unique to HaCaT cells; a similar increase was detected in other epithelial models displaying flocking motion, such as the A431 cell line and the primary explants of bronchial epithelial cells in ALI cultures (Figure S3F–G, Supporting Information).

These results indicate that connexins are consistently elevated upon EGF stimulation across multiple models, suggesting their central role in the cellular response to EGF.

### 2.4 EGF‐Induced Flocking Motility is Accompanied by Large Fluctuations in Cell Density, Shape, and Volume

Connexins, the main components of gap junctions, are crucial for intercellular communication, enabling the direct exchange of fluids, ions, small molecules, and other cytoplasmic content between neighbouring cells. This cytoplasmic continuity allows cells to coordinate their activities, a process that becomes particularly important in dense cell collectives where synchronized responses are essential for movement and other collective behaviors. Given this role, we hypothesized that EGF stimulation could activate connexins and gap junctions to promote unjamming via flocking within the cell monolayer. This collective movement, we propose, is mediated by fluid exchange and fluctuations in cell volume, which facilitate a coordinated and dynamic transition from a static, jammed state to a flocking‐like, migratory state.

**Figure 4:**
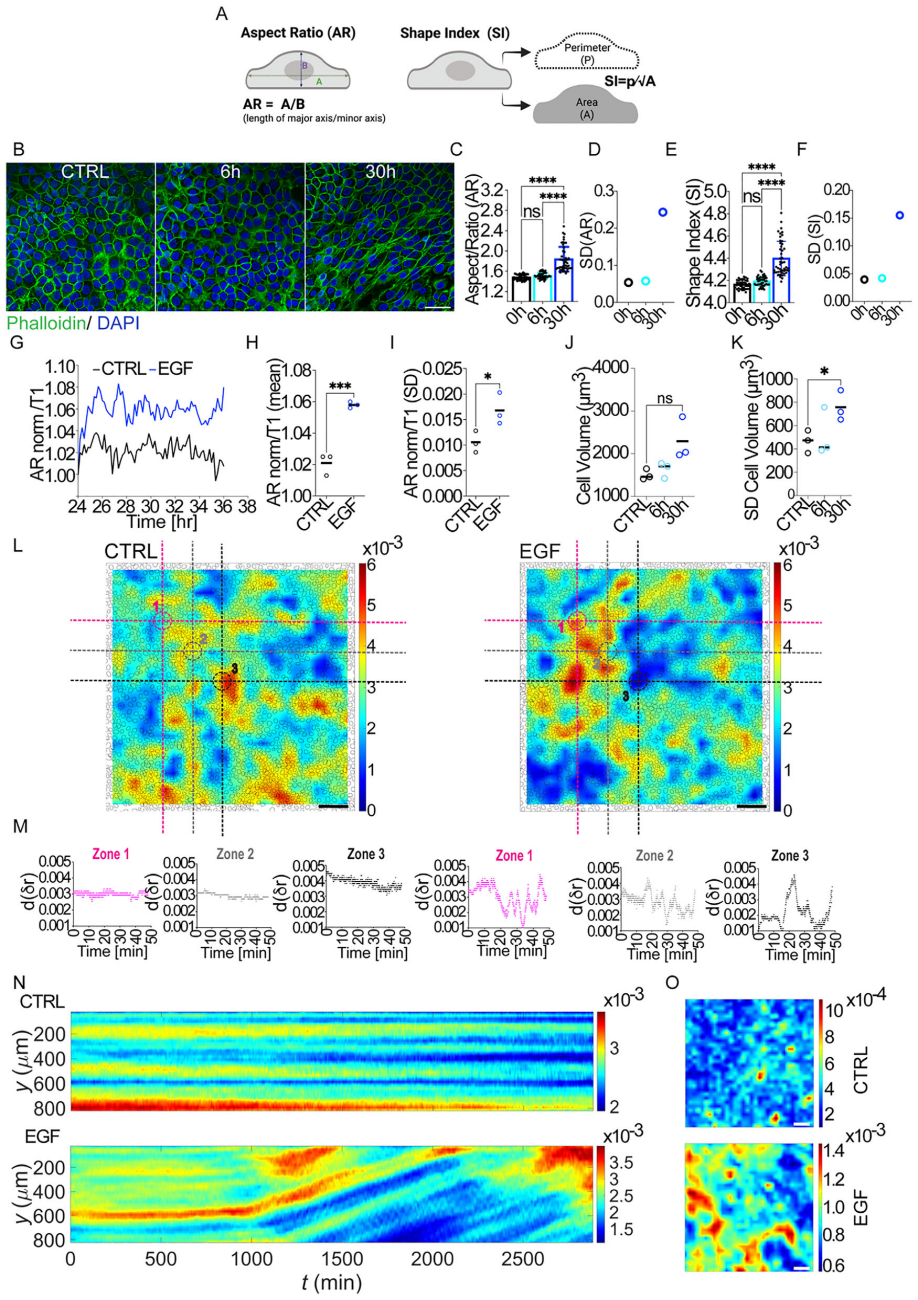
Cell shape fluctuations during EGF‐mediated flocking locomotion A) Diagrammatic inset depicting: Aspect Ratio (AR): length of major axis/minor axis. Shape Index (p⁄√A): perimeter divided by the square root of area. (Biorender) B–F) A. HaCaT cells are cultured to confluency, serum starved for 48 h, and treated with 100 ng mL^−1^ EGF for 6 or 30 h. Cells were fixed and stained with Phalloidin‐FITC to visualize Actin filaments and DAPI to visualize DNA/nuclei. (*n* = 38–48 fields of view analyzed from 3 independent experiments). B) Representative confocal images of cells fixed at different time points (CTRL, 6 and 30 h) and used for segmentation with CellPose and Fiji softwares, Scale bar 50µm. C) Analysis of Aspect /Ratio (AR) of HaCaT cells at 0, 6, and 24 h after EGF treatment. D) Standard deviation of Aspect Ratio. E) Analysis of the shape index (SI, Perimeter/SquareRootArea) at 0, 6, and 30 h as an indication of cell shape. F) Standard Deviation of Shape Index. G–I) Analysis of shape variations in HaCaT Ecadherin‐EGFP treated with EGF (100 ng mL^−1^) for 24 h before recording (*n* = 3 independent experiments). G) The time evolution of the Aspect/Ratio normalized to the time T1 is plotted. H) Normalized Aspect ratio Mean over 12 h. I) Standard deviation of AR. J,K) Analysis of cell volume fluctuations. HaCaT cells are cultured to confluency, serum starved for 48 h, and treated with 100 ng mL^−1^ EGF for 6 or 30 h Cells were fixed and stained with Phalloidin‐FITC to visualize Actin filaments and DAPI to visualize DNA/nuclei. (*n* = 3 independent experiments). J) Estimation of Cell volume by 3D segmentation. K) Standard Deviation of Cell Volume. L–O) Analysis of density fluctuations in HaCaT‐H2Bmcherry cells treated with EGF (100 ng mL^−1^) (*n* = 3 independent experiments). L) Density Distribution Across Monolayer Zones: segmentation images of nuclei of untreated (CTRL) (left) and EGF‐stimulated (right) HaCaT cells, respectively. The color bar represents the local cell density which scales linearly from 0 to 0.006 um^−2^. Scale bar 100 µm. M) Mean cell density [d(dr)] in a local circular region with a radius of 50 µm as a function of time. The pink, grey and black dots represent the mean densities in the three selected areas in pink (zone 1), grey (zone 2) and black (zone 3) dashed circles as shown in Figure L. N) Evolution of Cell density over time: kymographs of the density fields of untreated (top) and EGF stimulated (bottom) HaCaT cells, respectively. O) Fluctuations of cell density over time: colormaps showing the temporal density fluctuations of untreated (top) and EGF‐stimulated (bottom) HaCaT cells, respectively. The color of each pixel represents the standard deviation of the cell density at that point at different time moments. Scale bar 100 µm. Statistical tests and significance are indicated in Table 1.

To test this hypothesis, we first analyzed cell shape fluctuations in a starved HaCaT monolayer before and after EGF treatment. Shape analysis revealed a marked increase in both the cell aspect ratio and shape index at 30 h post‐EGF treatment—during the peak of collective motion—but not at 6 h, before flocking motion had emerged (**Figure** **4**A–F). This increase indicates a significant elongation of cell shape, which may be an adaptive response allowing cells to align and coordinate their movement as a group. Additionally, variance analysis showed large fluctuations in cell shape over time, coinciding with increases in the correlation length L_CORR_, confirming visual observations of heterogeneity in cell shape (Figure 4B,D,F). We confirmed the fluctuations in cell shape by monitoring E‐Cadherin‐EGFP expressing cells and extracting the aspect ratio, AR, defined as the ratio between the major and minor axes of fitted ellipses, as a function of time (Figure 4A,G–I; Movie S16, Supporting Information).

Estimates of cell volume obtained by a 3D segmentation algorithm indicate similar trends, with evidence of volume fluctuations occurring during the peak of collective motion (30 h post‐EGF) compared to starved untreated cells (Figure 4J,K; Movie S17, Supporting Information). Variations in cell volume during unjamming via collective flocking have been documented in other cellular systems, highlighting a potential role for these changes in facilitating coordinated motion.^[^ ^9^ ^]^

Notably, flocking monolayers display pronounced and sustained density variations that extend over the entire duration of collective motility, characterized by the coexistence of densely packed and more sparsely populated regions (Figure 4L). In contrast, starved (quiescent) HaCaT cells remain immobile within a dense monolayer and exhibit only minor, transient density fluctuations over time (Figure 4L,M). Upon EGF stimulation, however, the monolayer begins to stream—≈16 h post‐treatment—and robust fluctuations in local density emerge and persist (Figure 4L–N; Movie S18, Supporting Information). These are evident in the density maps (Figure 4L) and the time traces of mean cell density [d(δr)] from selected ROIs (zones 1, 2, and 3, Figure 4M), which show dynamic oscillations in cell packing in the EGF‐treated condition compared to the flat profiles in controls. Kymographs along the y‐axis (Figure 4N) reveal stable profiles in starved monolayers, while EGF‐treated cells exhibit clear spatiotemporal modulation of density. Fluctuation maps (Figure 4O) further underscore the increased temporal fluctuations of local cell density under EGF treatment. This evolving pattern of density and shape variation likely reflects continuous rearrangements within the monolayer, suggesting that cells dynamically adapt their positions and morphology in response to local cues.

Taken together, these observations support our hypothesis that fluctuations in cell shape, density, and volume, all emerging in response to EGF and evolving in parallel, strictly correlate with the emergence of flocking motion. We propose that these changes are mediated by gap junction activation, where connexins facilitate the exchange of cytoplasmic material, enabling cells to sense and respond to their neighbors’ states. This recruitment and activation of gap junctions may therefore be critical for driving the dynamic transition from a jammed, solid‐like state to a fluid‐like, flocking configuration, characterized by increased motility, local velocity alignment, and morphological remodeling. This specification distinguishes our observations from simpler unjamming transitions without flocking characteristics. We set out to demonstrate this contention through loss‐of‐function approaches.

### 2.5 Flocking Transition of Epithelial Ensembles Requires Functional Gap Junction Channels and Specifically Involves Cx26 and Cx31

To directly assess the involvement of gap junctions in the EGF‐induced fluid transition, we employ pharmacological and molecular genetic loss‐of‐function approaches.

**Figure 5:**
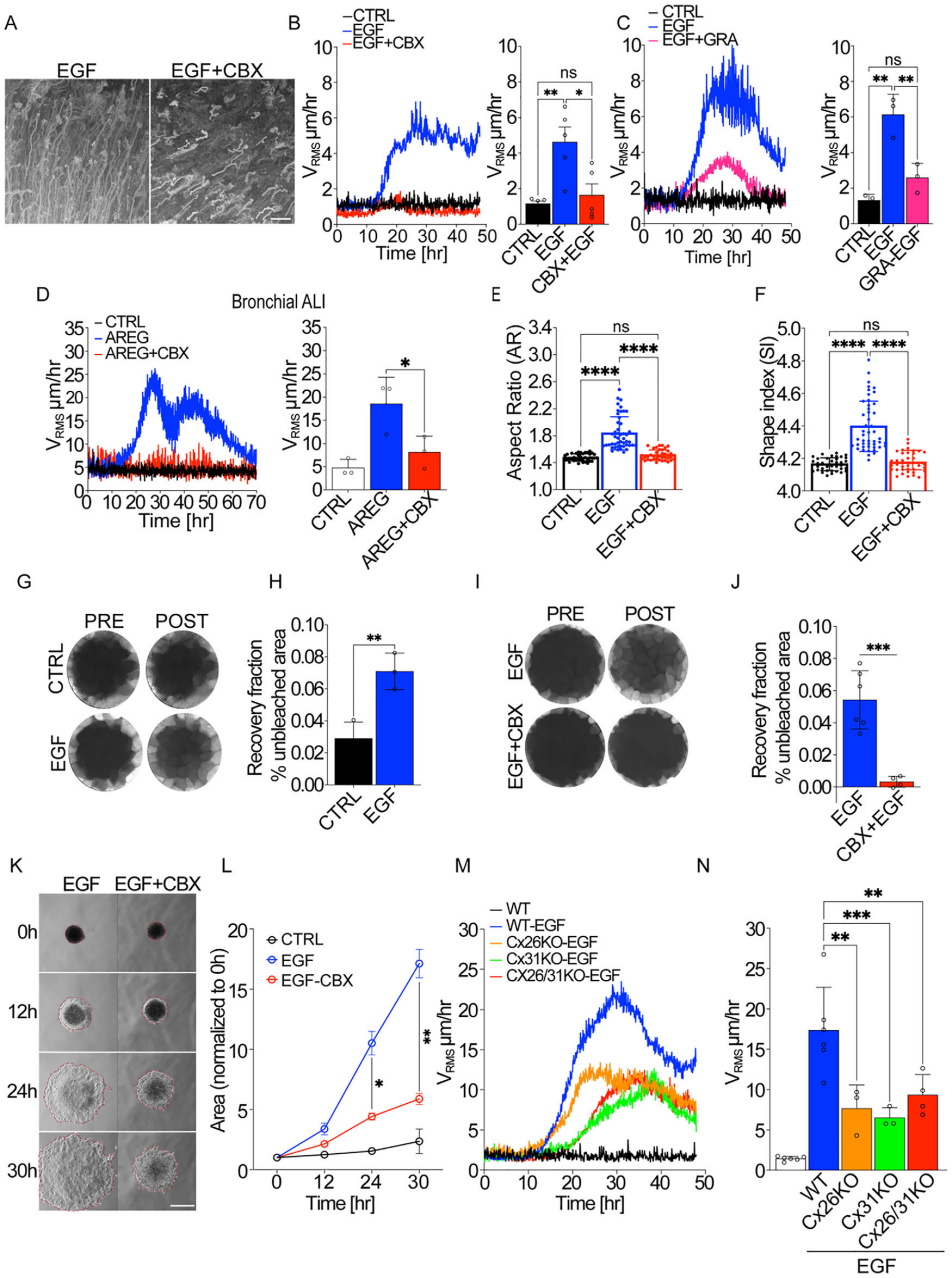
Shape variation and intercellular fluid transfer require functional Gap junction channels and specifically Connexins 26 and 31. A–C) HaCaT cells are cultured to confluency, serum starved, and treated with EGF 100 ng mL^−1^ in the absence or in the presence of Carbonexolone (CBX, 16 µm). (A,B) or 18beta‐Glycyrrhetinic acid (GRA, 10 µm) (C). A) Representative phase contrast image of the maximum intensity projection (MIP) of all frames acquired over a 24‐h period (5 min frame^−1^). Representative images from *n* = 5 independent experiments. Scale bar 100 µm. B,C) Root Mean Square Velocity (V_RMS_) measured by Particle velocimetry analysis (PIV) over 48h (left) and its mean within cell flocking framecut (right) in control or in cells treated with Connexin inhibitors. V_RMS_ is expressed as the mean ± SD (*n* = 3–5 independent experiments). D) Flocking locomotion induced by Amphiregulin (AREG) in Bronchial epithelial primary cultures in Air‐Liquid interface (ALI) in the absence (CTRL) or presence of 50 um Carbonexolone (CBX). Time evolution of the Root Mean Square Velocity (V_RMS_) measured by Particle velocimetry analysis (PIV) over 48 h (left) and its mean within cell flocking framecut (right). V_RMS_ is expressed as the mean ± SD (*n* = 3 independent experiments). E,F) Analysis of Aspect/Ratio (AR) (E) and shape index (SI) obtained through automatic cellpose segmentation (F) of HaCaT cells at 0 and 24 h following EGF treatment (100 ng mL^−1^) in the presence of DMSO (EGF), used as vehicle control for the pharmacological treatment, or 16 µm CBX (EGF‐CBX). (*n* = 38–48 fields of view analyzed from 3 independent experiments). G–J) Analysis of intercellular Dye transfer by FRAP (Fluorescence Recovery After Photobleaching) in HaCaT cells loaded with Calcein‐AM (20 µm, 45 min). The recovery of fluorescence was monitored by acquiring images before (Pre) and after (Post) the recovery. G) Representative images of epithelial cells loaded with a fluorescent dye (Calcein‐AM) in the control condition or treated with 100 ng mL^−1^ EGF for 24 h. H) Quantification of recovery of fluorescence over 10 min normalized to unbleached area. *n* = 10–12 fields of view analyzed from 3 independent experiments. I) Representative images of epithelial cells loaded with a fluorescent dye (Calcein‐AM) treated with EGF in the presence of CBX (EGF‐CBX, 16 µm) or DMSO EGF (EGF), used as vehicle control. J) Quantification of recovery of fluorescence over 30 min normalized to unbleached area. K,L) Active wetting of FN‐coated substrate of HaCaT spheroids. HaCaT cells were treated with EGF in the presence of CBX (EGF‐CBX, 100 µM) or DMSO (EGF), used as a control. K) Representative phase contrast images of spheroids at different timepoints during active wetting. The spheroid contour is indicated by a dotted line. (*n* = 3 independent experiments). Scale bar 500 µm. L) Quantification of the spreading area at different timepoints normalized to T0h. M,N) Collective flocking motility induced by EGF treatment in HaCaT clones following depletion of Connexin 26, Connexin 31, or both (Cx26KO, Cx31KO, and Cx26/31KO respectively). Time evolution of the Root Mean Square Velocity (V_RMS_) measured by Particle velocimetry analysis (PIV) over 48 h (left) and its mean within the 10–40 h framecut (right). V_RMS_ is expressed as the mean ± SD (*n* = 3–6 independent experiments). Statistical tests and significance are indicated in Table 1.

First, we stimulated HaCaT cells with EGF in the presence of gap junction (GJ) blockers. Treatment with Carbenoxolone (CBX), and to a lesser extent with its analogue 18β‐glycyrrhetinic acid (18βGA), completely inhibited the EGF‐induced flocking motion in HaCaT cells (**Figure** **5**A–C; Movies S19 and S20, Supporting Information). A similar inhibitory effect was observed in primary bronchial epithelial ALI cultures, where CBX also abolished EGF‐induced collective motility (Figure 5D; Movie S21, Supporting Information). Importantly, blocking GJ channels not only disrupted flocking motion but also prevented the EGF‐induced cell shape changes typically associated with this transition (Figure 5E,F). Conversely, we also evaluated a pharmacological activator of gap junctions, valproic acid, recently shown to enhance the channel activity of several connexins.^[^ ^55^ ^]^ While this agent alone was not sufficient to trigger flocking in the absence of EGF, the co‐treatment with EGF significantly enhanced collective motility (increased V_RMS_) in HaCaT monolayers (Figure S4A,B and Movie S22, Supporting Information). These findings support the notion that connexins are required and their activation potentiates EGF‐induced flocking, even if not sufficient on their own.

Importantly, Carbenoxolone treatment did not alter the expression of junctional proteins, their organization, or morphology as detected by immunoblotting, immunofluorescence (Figure S4C,D, Supporting Information), and ultrastructural Electron Microscopy analysis (Figure S5A, Supporting Information). It also did not affect the levels of EGFR phosphorylation (despite a slight reduction in total EGFR) nor of phosphorylated ERK1/2 and AKT, indicating that EGF signaling remains intact in the absence of functional connexins (Figure S5B, Supporting Information).

To further confirm the role of cytoplasmic fluid exchange in this transition, we performed fluorescent recovery after photobleaching (FRAP) using Calcein‐AM, a membrane‐impermeable dye that diffuses between cells only through gap junctions. Under normal conditions, FRAP analysis revealed enhanced fluid transfer during EGF‐induced flocking (Figure 5G,H; Movie S23, Supporting Information). However, this transfer was entirely blocked by CBX (Figure 5I,J; Movie S24, Supporting Information).

We also investigated gap junction communication in a 3D spheroid model. This process, described as an active wetting,^[^ ^56^ ^]^ is driven by a tug of war between intrinsic bulk mechanical and rheological properties of the cell spheroid and the ability to exert traction on the substrate. In this context, a “more fluidized” cell ensemble with flocking‐like dynamics properties is expected to behave analogously to low‐viscosity droplets that spread more rapidly (akin to water) compared to more solid‐like or viscous spheroids (akin to honey or oil). Importantly, this setup models the transition of a carcinoma from a confined, non‐invasive state to one capable of outward dissemination, thereby providing a physiologically relevant framework to assess the impact of altered collective dynamics.^[^ ^56^, ^57^, ^58^ ^]^ HaCaT cells were cultivated as cohesive spheroids under low‐attachment conditions, then plated on fibronectin‐coated ECM to induce a wetting transition. Treatment with a high dose of EGF significantly accelerated the wetting velocity (measured as the increase in spheroid area over time normalized to the initial area), compared to untreated spheroids. The addition of CBX significantly reduced the EGF‐enhanced wetting velocity. These observations highlight the importance of connexin‐mediated gap junctions in facilitating collective spreading at the basal interface between spheroids and the substrate. Although spheroids are 3D, the observed migratory activity primarily occurs within the 2D plane at the cell‐substrate contact zone, and we do not infer substantial motility within the spheroid interior (Figure 5K,L; Movie S25, Supporting Information). Additionally, the EGF‐induced increase in spheroid spreading area over time, accompanied by enhanced basal deformation, is indicative of a wetting‐like transition. While we do not directly measure interfacial tension, the observed dynamics are consistent with classical physical models of wetting transitions, where increased fluidity leads to faster and more extensive spreading of tissue‐like droplets on compliant substrates.^[^ ^56^, ^58^, ^59^ ^]^ In our system, this behavior provides further support to the central hypothesis that transcriptionally regulated fluid exchange modulates 2D tissue rheology, enabling transitions from a jammed to a flocking state that promotes outward migration and spreading.

To identify the specific connexins involved, we used CRISPR‐Cas9 to create knockout (KO) clones of HaCaT cells lacking Cx26 (GJB2) and/or Cx31 (GJB3). These connexins were chosen due to their strong transcriptional upregulation following EGF stimulation (Figure 3D–E) and their high baseline expression levels in HaCaT cells and other epithelial models. While protein‐level validation was attempted, available antibodies did not yield specific signals, likely due to isoform cross‐reactivity and low sensitivity. Therefore, gene selection was based on transcriptional profiling and the observed functional impact of gene knockout on flocking transitions. We generated single knockouts (Cx26KO and Cx31KO) and a double knockout (Cx26/31KO) from Cas9‐expressing HaCaT cells (**Table** = **2**). Particle image velocimetry (PIV) analysis of these knockout clones revealed that loss of Cx26 or Cx31 significantly reduced collective motility and impaired GJ coupling (Figure S6A,B, Supporting Information). Specifically, mean velocities dropped to 7.66 ± 2.93 µm h^−1^ in Cx26KO, 6.51 ± 1.25 µm h^−1^ in Cx31KO, and 9.4 ± 2.53 µm h^−1^ in Cx26/31KO clones, compared to 17.37 ± 5.33 µm h^−1^ in wild‐type cells (Figure 5M,N; Movie S26, Supporting Information). Single or double knockouts of connexins did not completely abolish EGF‐induced flocking motion, suggesting the involvement of additional mechanisms or compensatory upregulation of other connexin family members. Consistent with the latter, gene expression analysis of Cx26 and Cx31 knockout clones revealed increased expression of alternative connexins, likely reflecting a compensatory response (Figure S6C–E, Supporting Information). Notably, while knockdown of *GJB2* and *GJB3* led to upregulation of *GJB6*, the CRISPR double knockout failed to induce *GJB6* but instead upregulated *GJC1* expression. This apparent non‐linear compensation suggests distinct regulatory circuits governing connexin gene expression, potentially mediated by chromatin context or promoter‐specific feedback mechanisms.

These findings underscore the critical role of gap junctions, particularly Cx26 and Cx31, in the fluid‐like transition that drives collective migration. Gap junction‐mediated cytoplasmic fluid exchange, supported by specific connexins, is likely essential for enabling the unjamming and coordinated movement of epithelial cells in response to EGF. Importantly, while our data firmly establish that connexin upregulation is indispensable for EGF‐induced flocking, we do not conclude that connexins are independently sufficient to promote this behavior. Rather, they operate in conjunction with additional EGF‐dependent processes, including ERK activation and lamellipodial remodeling, to enable the transition.

**Table 2:** Connexins Knockout clones selected following Crispr‐Cas9 gene editing. Table summarizing the selection and validation of CRISPR/Cas9‐generated knockout clones targeting different connexin genes (GJB2, GJB3, GJB6, GJA1) in VFC cells. Each row reports the targeted gene, the genotype efficiency (ICE indel %), KO‐Score (predicted knockout efficiency), and the R^2^ value (goodness of fit for the ICE analysis). The “Indels” column specifies the types of insertions/deletions identified. Double knockout clones (targeting two connexins simultaneously) are also indicated. Target sequences and corresponding exons used for CRISPR editing are listed at the bottom. Clones were selected based on high indel frequency, high KO‐score, and confirmed disruption of the target loci. Clones harboring frameshift mutations predicted to abrogate protein production were selected for further experiments. Although mRNA transcripts may still be detected by qRT‐PCR, these clones are functionally null for Cx31 due to the generation of out‐of‐frame transcripts incapable of producing protein.

Selected clones list [single KOs]								
Clone	Gene	Genotype	ICE [indel %]	Ko‐Score	R^2^	Indels		
3–11	GJB2	KO	98	98	0.98	{'1': 98.0}		
3–22	GJB2	KO	97	97	0.97	{'1': 26.0, '−1': 34.0, '−2': 37.0}		
6–1	GJB3	KO	99	99	0.99	{'1': 99.0}		
6–6	GJB3	KO	99	99	0.99	{'1': 26.0, '−2': 73.0}		
Clones list								
	Genotype		GJB2 locus			GJB3 locus		
Clone	GJB2	GJB3	KO‐score	R^2^	Indels	KO‐score	R^2^	Indels
36–2	KO	KO	94	0.95	{'0': 1.0, '−1': 31.0, '−2': 32.0, '−10': 31.0}	98	0.98	{'1': 47.0, '−2': 51.0}
36–41	KO	KO	98	0.98	{'1': 68.0, '−2': 30.0}	98	0.98	{'1': 70.0, '−5': 28.0}
36–60	KO	KO	97	0.97	{'1': 74.0, '−4': 23.0}	97	0.97	{'1': 30.0, '−1': 32.0, '−2': 6.0, '−4': 29.0}
36–84	KO	KO	97	0.97	{'1': 49.0, '−2': 48.0}	97	0.97	{'−1': 29.0, '−5': 36.0, '−7': 32.0}
sgRNAs								
Gene	Sequence	Exon						
GJB2	CCTCCTTTGCAGCCACAACG	2						
GJB3	CCACCACGTATACCAGCACC	2						

### 2.6 Vocal Fold Cancer Cells Exhibit a Sustained Flocking Motion Enhanced by EGF and Involving Gap Junctions

Vocal fold cancer (VFC) arises from transformed keratinocytes in the unique, mechanically dynamic environment of the vocal folds, where normal tissue function relies on a well‐organized extracellular matrix (ECM) for elasticity and vibration. In VFC, however, ECM remodeling leads to increased stiffness, which disrupts tissue mechanics and drives cancer progression by activating mechanosensitive pathways, particularly YAP/TAZ signaling. This enhanced rigidity supports aggressive tumor behaviors, including a shift toward collective “flocking” motility, which facilitates invasion.^[^ ^35^ ^]^ As VFC progresses from early‐stage (T1) to advanced‐stage (T3‐T4), tumor cells invade deeply into the underlying collagen‐rich lamina propria and muscle, resulting in the mechanical fixation characteristic of later stages. Because VFC is highly sensitive to mechanical cues, studying the flocking transition in this context may reveal key targets that are expected to control the invasive behaviors in this mechanically driven cancer.

**Figure 6:**
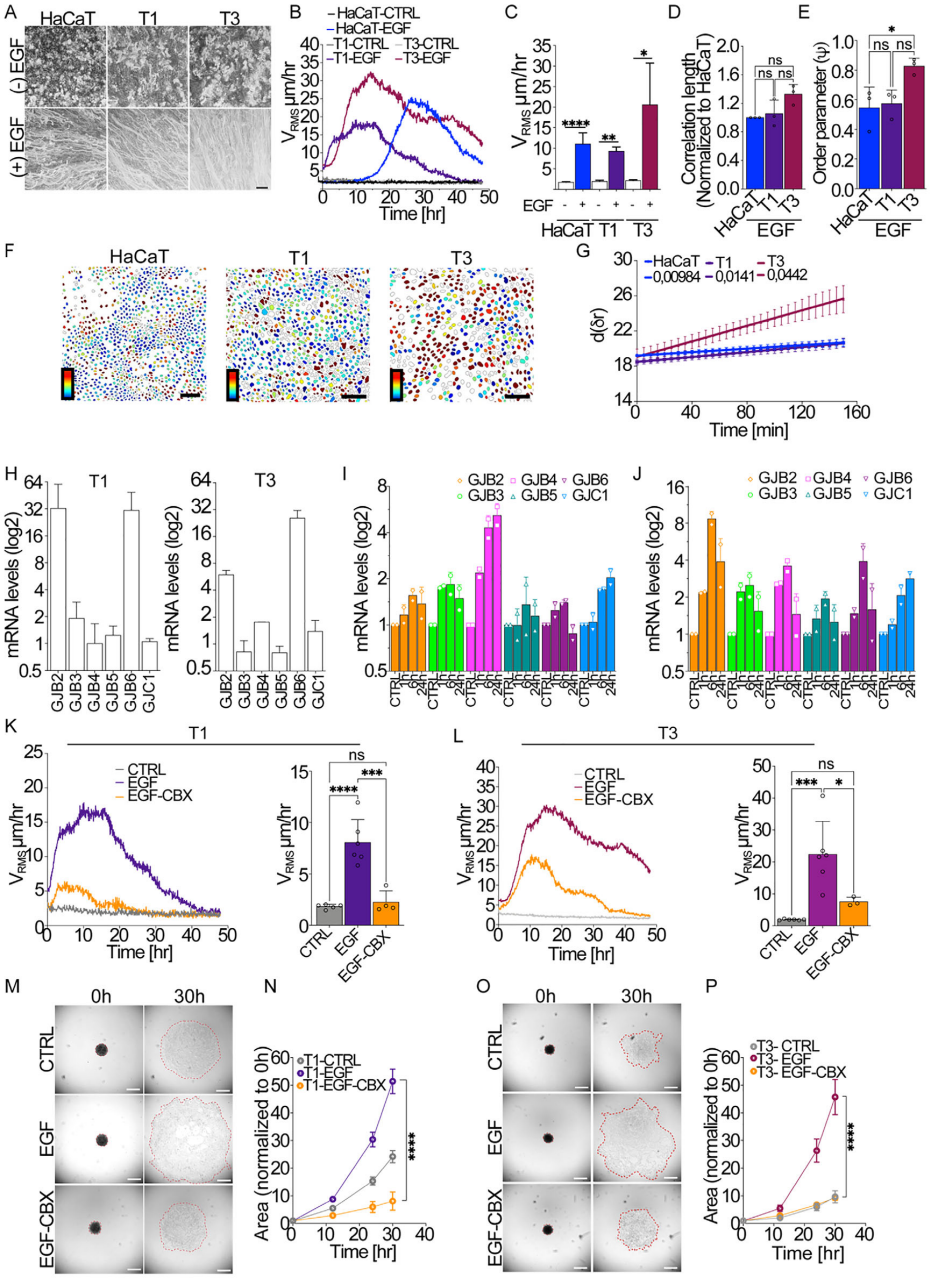
Stage I and stage III Vocal fold cancer cells exhibit a sustained EGF‐induced flocking motion involving gap junctions. A–E) Collective motility induced in serum‐deprived HaCaT, VFC T1, and T3 cells upon EGF (100 ng mL^−1^) treatment. A) Representative phase contrast image of the maximum intensity projection (MIP) of all frames acquired over a 24‐h period (5 min frame^−1^). Representative images from *n* = 3–5 independent experiments. Scale Bar, 100 µm. (B,C) Time evolution of the Root Mean Square Velocity (V_RMS_) measured by Particle velocimetry analysis (PIV) over 48 h (left) and its mean within the 10–40 h framecut (right). (*n* = 3 independent experiments, each including multiple fields of view (FOVs)). D) Normalized *C*orrelation lengths *l_corr_*, used as indicator of the collective nature of cell motion obtained by calculating the velocity correlation length L_COOR_ as the width of the correlation function *C*_V V_(r) = (〈v(x_0_ + r) v(x_0_)〉)/〈|v(x_0_)|^2^〉 of the (vectorial) drift‐corrected velocity v(x_0_) evaluated in the time window comprised between 15 and 40 h after EGF induction of flocking migration as previously described and normalized to the *L_CORR_* of HaCat monolayers, used as control. E) Order parameter (ψ) on 15–40 h period (*n* = 3 independent experiments, each including multiple fields of view (FOVs)). F,G) Neighbor exchange during collective motility in HaCaT, VFC T1 and T3 cells upon EGF (100 ng mL^−1^) treatment. F) The segmentation images of HaCaT, T1, and T3 cell nuclei (from left to right) during flocking, respectively. The color bar represents the change of distance between the reference cell and its neighbors which scales linearly from 0 to 5 um. Scale bars 100 µm. (G) The mean separation distance (*d*) between cells and their initial neighbors as a function of time delay (*δt*). The solid lines are linear fits. H) mRNA levels of Connexins genes in VFC T1 (left) and T3 (Right) cells, quantified by qRT‐PCR. Data are the mRNA fold increase relative to the levels of control cells after normalizing for GAPDH and 18S mRNA levels (*n* = 3 independent experiments). I,J) mRNA levels of Connexins genes in VFC T1(E) and T3 (F) cells treated with EGF for 1, 6, and 24 h; quantified by RT‐qPCR. Data are the mRNA fold increase relative to the levels of control cells after normalizing for GAPDH and 18S mRNA levels (*n* = 2 independent experiments). K,L) Time evolution of the Root Mean Square Velocity (V_RMS_) measured by Particle velocimetry analysis (PIV) over 48 h (left) and its mean within the 10–40 h framecut (right) in VFC T1 (K) and T3 (L) cells treated with EGF in the presence of Carbenoxolone (EGF‐CBX) or DMSO (EGF). K) Serum‐deprived T1 cells treated with EGF in the presence of 16 µm Carbenoxolone (EGF‐CBX) or DMSO (EGF). Data are mean (± SD) (*n* = 4–5 independent experiments). L) Serum‐deprived T3 cells treated with EGF in the presence of 20 µM Carbenoxolone (EGF‐CBX) or DMSO (EGF). Data are mean (± SD) (*n* = 10–14 fields of view analyzed from 2‐3 independent experiments). M–P) Active wetting of FN‐coated substrate of VFC T1 (M,N) and T3 (O,P) spheroids. Spheroids were treated with EGF in the presence of CBX (EGF‐CBX, 150 µm for T1 and 175 um for T3 spheroids) or DMSO (EGF), used as a control. (*n* = 3 independent experiments). M,O) Representative phase contrast images of spheroids at different timepoints during active wetting. The spheroid contour is indicated by a dotted line. Scale bar 500 µm. N,P) Quantification of the spreading area at different timepoints normalized to T0h. Statistical tests and significance are indicated in Table 1.

In previous studies, we found that both T1 and T3 VFC cells exhibit a delayed jamming transition compared to non‐tumorigenic HaCaT cells.^[^ ^35^ ^]^ In response to EGF stimulation, all three keratinocyte lines (HaCaT, T1, and T3) undergo a transition from a quiescent, jammed state to a collectively motile phase. This transition occurs more rapidly in the oncogenic VFC cell lines (T1 and T3) than in the non‐tumorigenic HaCaT monolayer (**Figure** **6**A; Movies S27 and S28, Supporting Information). Quantification of V_RMS_ over time shows a robust increase in motility following EGF treatment, with the T3 line exhibiting the highest velocity amplitude among the three, bypassing the delay typically observed in HaCaT monolayers. Additionally, this collective migration was notably persistent in T3 cells (Figure 6B,C). Furthermore, T3 cells display higher correlation length and order parameter (Ψ), as compared to HaCat and T1 cells; although the correlation length increase did not reach statistical significance, both metrics are consistent with a constitutively elevated flocking phenotype (Figure 6D,E).

To probe the mechanical nature of this collective behavior, we analyzed cell rearrangements by measuring changes in the average distance, *d*, between cells and their initial neighbors as a function of time delay, *δt* (Figure 6F,G; Movie S29, Supporting Information). This analysis reveals a striking divergence in neighbor exchange dynamics between the lines. HaCaT and T1 cells display minimal changes in neighbor identity during flocking, indicating that cells migrate cohesively without significant local rearrangements. This behavior is consistent with a “flocking solid” state, in which coordinated migration occurs within a topologically constrained configuration—a phenomenon we recently characterized in epithelial tissues as a form of active jamming typical of scale‐free flocking in epithelial active solids.^[^ ^35^, ^48^ ^]^ In contrast, T3 cells exhibit a monotonic increase in neighbor exchange compared to HaCaT and T1 cells during migration, as evidenced by a much larger growth rate of *d*(*δt*) (Figure 6F,G; Movie S29, Supporting Information). This dynamic behavior indicates a fluid‐like reorganization within the monolayer and suggests that T3 cells adopt a “flocking‐liquid” mode of collective migration.

These findings imply that although all three lines undergo a flocking transition upon EGF stimulation, the mechanical nature of this transition varies: HaCaT and T1 cells behave as flocking solids, maintaining persistent neighbor relationships, while the aggressive T3 cells exhibit liquid‐like properties characterized by enhanced cellular rearrangements. This distinction may underlie differences in the capacity for tissue remodeling and invasiveness, and echoes recent work demonstrating that fluctuations and neighbor exchange in epithelial collectives can discriminate between jammed solid‐like and fluid‐like flocking regimes.^[^ ^48^ ^]^

Next, we investigated whether gap junctions (GJs) and connexins are involved in EGF‐stimulated flocking motion in VFC cells. Notably, VFC did not display altered levels of total EGFR and EGFR signaling as compared to the non‐tumorigenic keratinocytes (Figure S7, Supporting Information). Analysis of connexin mRNA levels in T1 and T3 cells revealed elevated baseline expression of certain connexins compared to HaCaT cells. Both VFC stages showed increased mRNA levels of connexins, such as Cx26 (GJB2) and Cx30 (GJB6), while Cx31 (GJB3) was upregulated in T1 cells but not significantly affected in T3 cells (Figure 6H).

Upon EGF stimulation, connexin expression in T1 and T3 VFC cells was upregulated, with a marked increase in mRNA levels of key connexins (Figure 6I,J).

To confirm the role of GJs in EGF‐driven flocking motion, we treated T1 and T3 VFC monolayers with EGF in the presence of the GJ inhibitor, Carbenoxolone. Similar to our findings in HaCaT cells, GJ inhibition led to a substantial reduction in cell motility. However, this inhibitory effect was partial, suggesting that while GJs play a critical role in facilitating collective motility, additional mechanisms may also contribute to the motility of T1 and T3 VFC cells (Figure 6K,L; Movies S30 and S31, Supporting Information).

In parallel, we also examined the role of gap junction communication in a 3D spheroid model of active wetting by cultivating T1 and T3 VFC cells under low‐attachment conditions to develop spheroids, before plating them on fibronectin‐coated ECM to induce active wetting. Similarly to what was observed in HaCaT spheroids, treatment with a high dose of EGF significantly accelerated the wetting velocity compared to untreated spheroids. The addition of CBX significantly reduced the EGF‐enhanced wetting velocity, confirming the importance of gap junctions in promoting collective cell dissemination during a 3D‐to‐2D transition (Figure 6M–P; Movies S32 and S33, Supporting Information). Notably, active wetting of spheroids reflects the interplay between cell–cell cohesion (viscosity) and cell–substrate traction, and while we observe that Carbenoxolone inhibits wetting, this compound—at the concentrations used—does not exhibit cytotoxic effects in HaCaT keratinocytes, nor is there evidence of major disruption to intercellular or cell–cell adhesion in this cell type or other epithlial line as we also shown (Figures S4 and S5, Supporting Information),^[^ ^60^, ^61^ ^]^ Therefore, it is most parsimonious to interpret the reduced wetting velocity as a consequence of disrupted collective cell dynamics.

These findings underscore the involvement of connexins and GJs in the sustained collective migration observed in VFC cells, particularly in response to EGF stimulation. The upregulation and prolonged expression of specific connexins, such as Cx26, may enhance intercellular communication, supporting the flocking behavior that likely contributes to the invasive potential of VFC at more locally advanced stages. To bridge our mechanistic observations with clinical relevance, we used the Kaplan–Meier Plotter tool to evaluate whether differential elevated expression of specific connexins correlates with overall survival in breast cancer patients. To this end, we interrogated TCGA RNAseq datasets through the Kaplan–Meier Plotter tool.^[^ ^62^ ^]^ Elevated expression of GJB2 (Cx26) showed a significant and robust association with worse overall survival (OS) in multiple tumor types, including renal clear cell carcinoma, lung adenocarcinoma, pancreatic ductal adenocarcinoma, and cervical squamous cell carcinoma (Figure S8, Supporting Information). In contrast, no significant association was detected in head and neck squamous cell carcinoma, a cohort encompassing a heterogeneous mix of tumor subtypes beyond VFC, where the sample size or tumor diversity may have obscured the detection of subtype‐specific upregulation patterns. This analysis supports a potential prognostic role for connexin‐mediated fluid dynamics in carcinoma progression.

## 3 Discussion

In this study, we unveil a novel mechanism driving the transition from a jammed to a flocking state in epithelial cell populations, a process pivotal for both physiological tissue dynamics and cancer metastasis. Central to this transition is the de novo transcription of specific connexin genes, particularly Cx26 and Cx31, which orchestrate cytoplasmic fluid exchange through gap junctional intercellular communication (GJIC). Our findings demonstrate that the upregulation of these connexins in response to epidermal growth factor (EGF) stimulation facilitates large‐scale cell volume fluctuations and density heterogeneity, thereby enabling collective cell migration. Importantly, such fluctuations are negligible in the starved condition and remain minimal in non‐starved monolayers, where endogenous EGF signaling is insufficient to trigger coordinated motility, underscoring the necessity of acute EGF activation to induce these dynamics. The disruption of connexin function, either pharmacologically or via genetic knockout, significantly impairs—but does not completely abolish—the emergence of coordinated flocking cell motility and active wetting, indicating that gap junction‐mediated cytoplasmic fluid exchange is necessary to enable this transition, though not alone sufficient, and may act in concert with other pathways or compensatory mechanisms. Moreover, the sustained flocking behavior observed in vocal fold carcinoma (VFC) cells and the linkage between Cx26 upregulation and tumor aggressiveness, contingent upon connexin activity, highlights the potential pathological relevance of this mechanism in cancer progression.

Biological systems exhibit rheological transitions at confluence driven by the active generation of forces, distinguishing them from inert materials.^[^ ^44^, ^49^, ^63^, ^64^ ^]^ Minimal theoretical models have predicted that such transitions in fully confluent tissues are governed not only by the interplay between cell–cell adhesion and cortical tension but also by the speed and persistence of single‐cell motility.^[^ ^8^, ^63^, ^65^, ^66^ ^]^ Our data extend these results by demonstrating that EGFR signaling, through the induction of connexin expression, plays a pivotal role in mediating these transitions.

Previous studies have identified EGFR as a critical regulator of tissue fluidization, particularly in airway epithelia, where mechanical compression or biochemical signals can activate EGFR pathways, subsequently triggering ERK1/2 signaling.^[^ ^5^, ^67^, ^68^ ^]^ Similarly, direct stimulation of EGFR with ligands such as amphiregulin or cytokines like IL‐6 has been shown to induce collective fluidization via YAP activation and actomyosin signaling.^[^ ^46^, ^69^ ^]^ In quiescent monolayers of human keratinocytes, serum‐induced fluidization also relies on EGFR, ERK, and actomyosin pathways.^[^ ^29^, ^70^ ^]^ Moreover, enhanced expression of the endocytic protein RAB5a in mammary epithelia promotes EGFR internalization, leading to hyper‐activation of ERK1/2 and phosphorylation of WAVE2, which are associated with increased traction forces and coordinated cell displacements.^[^ ^3^, ^49^ ^]^ Our findings integrate seamlessly into this existing framework by revealing that EGFR not only activates ERK1/2 and AKT signaling pathways but also drives the de novo transcription of connexins, thereby introducing a new layer of regulation in collective cell behavior. This transcriptional activation links extracellular EGFR signals to the intracellular machinery that governs collective dynamics, providing a mechanistic insight into how external stimuli can induce large‐scale cellular reorganization.

The upregulation of connexins Cx26 and Cx31 represents a critical molecular advancement in understanding how cell–cell communication facilitates collective behaviors. Importantly, although connexin upregulation is required for this transition, it likely functions as part of a broader program of EGF‐mediated reactivation, including transcriptional, cytoskeletal, signaling, and mechanical components.^[^ ^3^, ^49^ ^]^ Thus, connexins are essential enablers, but not stand‐alone initiators, of the flocking transition. Connexins form gap junction channels that enable the direct exchange of ions, small molecules, and cytoplasmic components between neighboring cells. Our data indicate that while cytoplasmic fluid exchange through gap junctions might be a passive process, its facilitation depends on the active transcriptional upregulation of connexins in response to EGF stimulation. This molecular regulation enables the emergence of cell volume fluctuations and density heterogeneity necessary for the flocking transition. Importantly, the elevation of gap junction‐mediated fluid exchange works in concert with localized contractile activity or volume changes induced by mitogen signaling, in line with previous findings showing rapid activation of mechanical stress and intercellular pressure gradients in quiescent epithelial sheets following serum exposure.^[^ ^29^, ^31^, ^71^ ^]^ Such gradients can drive passive redistribution of water and solutes via connexins, thus enabling coordinated cell swelling/shrinkage and contributing to the emergence of collective motility.

In our experimental models, inhibition or genetic knockout of Cx26 and Cx31 compromises flocking transition and collective motility, underscoring the indispensable role of these connexins in mediating cytoplasmic fluid exchange. This finding is particularly significant as it provides a molecular basis for the active regulation of tissue rheology through connexin‐mediated communication. By facilitating synchronized cell volume changes, connexins introduce the necessary fluctuations that overcome the energy barriers associated with transitioning from a solid to a fluid‐like state. This mechanism aligns with theoretical predictions that active fluctuations at the single‐cell level can drive rheological transitions in confluent tissues.^[^ ^8^, ^9^, ^35^, ^63^, ^66^ ^]^ Notably, we employed pharmacological and CRISPR‐Cas9‐mediated gene editing of Cx26 and Cx31. Direct assessment of protein depletion was not possible due to the lack of reliable isoform‐specific antibodies under our experimental conditions. To overcome these limitations, we ensured stable genetic inactivation as detected by Tracking of Indels by Decomposition (TIDE) (Table 2). Importantly, the ablation of Cx26 or Cx31 impairs, but does not abolish, EGF‐induced flocking and partially reduced gap junction–mediated cell coupling (Figure S6A,B, Supporting Information). This residual coupling is consistent with compensatory upregulation of alternative connexins, as confirmed by qRT‐PCR (Figure S6C–E, Supporting Information). This reciprocal compensation suggests feedback regulatory mechanisms and highlights the challenge of fully suppressing GJIC through targeted genetic silencing of individual isoforms. These findings underscore the robustness of gap junction‐mediated coupling in epithelial tissues and the likely presence of redundant or plastic expression programs that preserve intercellular connectivity. Interestingly, our transcriptomic analysis revealed a non‐linear pattern of compensatory connexin expression: while single KO of either GJB2 (Cx26) or GJB3 (Cx31) led to upregulation of GJB6 (Cx30)—a closely related isoform—the double KO did not elicit a similar GJB6 response. Instead, the dual KO induced upregulation of GJC1 (Cx45), a more distantly related connexin associated with different gating and permeability properties. This divergence suggests that feedback regulation of connexin gene expression is not merely additive but may involve context‐specific buffering or hierarchical compensation mechanisms. One possibility is that the presence of either Cx26 or Cx31 is required to trigger the GJB6 compensatory axis, whereas their simultaneous absence shifts the cellular response toward activation of alternate pathways, such as GJC1. Such behavior has precedent in other multigene families with overlapping function, where regulatory cross‐talk modulates isoform deployment based on cellular need and stress signaling. While further functional studies are required to dissect whether this compensation maintains residual GJIC or merely reflects transcriptional adaptation, our findings highlight the plasticity and complexity of the connexin network in sustaining epithelial coordination.

While our data strongly support a role for connexins in facilitating intercellular cytoplasmic fluid exchange and volume modulation, we recognize that connexin channels may also enable the passage of signaling molecules or ions, such as ATP or calcium, which could contribute to the emergence of flocking transitions. Although the requirement for intercellular ATP signaling has been proposed in other models of collective migration,^[^ ^22^, ^72^ ^]^ these mechanisms largely reflect paracrine ATP release and purinergic signaling rather than direct intercellular ATP exchange through gap junctions. As such, while ATP passage through connexins is possible, its sufficiency or necessity in driving flocking transitions remains unproven and would require further dedicated experiments. We are currently investigating whether calcium transients, which can also propagate through gap junctions, may play a concurrent role. Further studies will be needed to dissect the relative contribution of metabolite and ion fluxes in driving the flocking behaviors described here. Additionally, we also acknowledge that connexins can signal via hemichannel activity or cytoskeletal scaffolding. Although the former has not been directly tested in our system, it may be partially affected by carbenoxolone treatment. The latter, well‐documented for Cx43, remains less defined for Cx26 and Cx31, yet could represent an additional mode of functional contribution deserving future exploration.

Furthermore, the sustained flocking behavior observed in vocal fold carcinoma (VFC) cells, which exhibit elevated connexin expression, highlights the pathological relevance of this mechanism in cancer progression. In the mechanically dynamic environment of the vocal folds, connexin‐mediated cytoplasmic fluid exchange supports the invasive potential of cancerous cells by maintaining the fluid‐flocking‐like state necessary for collective migration.^[^ ^35^ ^]^ This is consistent with our broader findings that connexins play a dual role in both physiological tissue dynamics and pathological invasion.

The identification of connexin‐mediated cytoplasmic fluid exchange as a mediator of collective cell migration has profound implications for understanding cancer metastasis. Traditional views of connexins have oscillated between their roles as tumor suppressors and promoters of metastasis.^[^ ^13^, ^25^, ^26^, ^27^, ^28^ ^]^ Our study reconciles these perspectives by positioning connexins as key facilitators of collective invasion through their ability to mediate intercellular communication and cytoplasmic fluid exchange. This dual functionality underscores the complexity of connexin roles in cancer biology and highlights their potential as therapeutic targets. Consistently, elevated Cx26 expression is significantly associated with reduced overall survival across several tumor types, particularly in carcinomas whose progression is promoted by a mechanically perturbed microenvironment, such as vocal fold carcinoma and pancreatic ductal adenocarcinoma.

In the context of VFC, the persistent flocking behavior driven by connexin activity suggests that targeting gap junctions could impede the invasive capabilities of cancer cells. Pharmacological inhibition of connexins partially disrupted collective motility in both physiological and cancerous epithelial models, pointing to the feasibility of connexin inhibitors as potential therapeutic agents to curb metastasis, particularly in tumors that are mechanically challenged as VFC.

Beyond cancer, our findings have broader implications for understanding tissue morphogenesis, wound healing, and regenerative medicine. The ability to manipulate connexin expression and function could enhance collective cell migration in tissue repair processes or, conversely, stabilize the jammed state to prevent unwanted cell migration in pathological conditions. Future research should explore the interplay between connexin‐mediated cytoplasmic and other signaling pathways involved in collective cell behavior to fully elucidate the regulatory networks governing tissue dynamics. Moreover, integrating our molecular findings with biophysical models of tissue rheology can provide deeper insights into the emergent properties of cellular collectives. Understanding how connexin‐mediated fluctuations interact with mechanical forces and cellular adhesions will be crucial for developing comprehensive models that predict tissue behavior under various physiological and pathological conditions.

In summary, our study advances the understanding of collective cell migration by uncovering a connexin‐mediated mechanism that links EGFR signaling to cytoplasmic fluid exchange and cellular fluctuations as a necessary, albeit not sufficient for a flocking fluid transition. By demonstrating that de novo transcription of connexins is essential for flocking behavior in both physiological and cancerous epithelial systems, we provide a novel molecular framework that integrates extracellular growth signals with intracellular communication networks. These insights not only enhance the fundamental understanding of tissue dynamics but also identify potential therapeutic targets for mitigating cancer metastasis through the disruption of collective cell behaviors.

## 4 Experimental Section

### Cell Lines and Cultures

HaCaT (human immortalized keratinocytes, ATCC) and A431 (Skin Carcinoma, ATCC) cell lines were cultured in DMEM medium (Euroclone) supplemented with 10% FBS and 2 mm glutamine. Vocal Fold patient‐derived Cancer cell lines, UT‐SCC‐11 (Early T1 human glottic laryngeal cancer, 58‐year old male, Turku University hospital), UT‐SCC‐103 (Advanced T3 human glottic laryngeal cancer, 51‐year old male, Turku University hospital) (T1 and T3 respectively) were kindly provided by Johanna Ivaska (University of Turku, Finland) and cultured in DMEM medium supplemented with 10% FBS, 2 mm glutamine and Non‐essential AminoAcids (NEAA). For HaCaT, A431, and VFC monolayers, the substrate was intentionally avoided coating with exogenous ECM proteins. HaCaT cells are known to produce and deposit their own ECM, particularly during the 48‐h preincubation period before EGF stimulation. Pilot experiments and literature precedent confirmed that this self‐deposited ECM supports robust adhesion and baseline epithelial architecture without additional coating. Bronchial epithelial cultures, primary human cells obtained via brush biopsy, were initially maintained under submerged culture conditions without ECM coating. This approach is consistent with the manufacturer's protocol (Epithelix, https://www.epithelix.com), which specifies that ECM coating is not required for initial adherence and expansion. Once confluent, the cells were differentiated over 1–2 weeks in air‐liquid interface (ALI) conditions, during which they deposited their own ECM as part of pseudostratified epithelium formation. Bronchial epithelial ALI primary cultures MucilAir were cultured in MucilAir complete medium. All cell lines were cultured at 37 °C and 5% CO_2_. All cells except Bronchial primary cultures were passaged every 2–3 days to maintain sub‐confluency.

For the packaging of lentiviral particles, HEK293T cells (obtained from the BBCF‐Biological Bank and Cell factory, INT, Milan, and grown in DMEM supplemented with 10% fetal bovine serum and 2 mm l‐glutamine) were used. For the generation of retroviral particles, Phoenix‐AMPHO cells (American Type Culture Collection, CRL‐3213) were used and cultured as recommended by the supplier.

Constitutive expression of mCherry H2B was achieved by retrovirus infection of HaCaT cells with the pBABE‐puro‐mCherry‐H2B vector.

### Constructs and Generation of Lentiviral Particles

Packaging of retroviruses and lentiviruses was performed following standard protocols. Viral supernatants were collected and filtered through 0.45 µm filters. Cells were subjected to 4 cycles of infection and FACS sorted to eliminate non‐fluorescent cells. After several passages, stable bulk populations were selected. For the pBABE vector, cells were selected using puromycin (2 µg mL^−1^).

The following Constructs were used:

- Fucci: pCS2‐EF‐MCS‐mKO2‐hCdt1, pCS2‐EF‐MCS‐mAG‐hGeminin (from Miyawaki lab).
- EGFP‐E‐cadherin: pLL5.0‐E.cad_shRNA/mEcad‐GFP (from Alpha Yap lab).
- H2B‐mCherry: pBABE‐puro‐mCherry‐H2B (IFOM).

EGFP‐E‐Cadherin is a fusion construct where the extracellular domain of human E‐cadherin is tagged with enhanced GFP to visualize adherens junctions. H2B‐mCherry is a fusion of histone H2B with mCherry fluorescent protein, enabling visualization of chromatin dynamics and nuclear tracking.

### Drug Treatments

Cells were treated with EGF or Amphiregulin (Peprotech) in the presence of the following drugs added at the same time, except otherwise indicated, and kept for the duration of the experiment: Y27632 (Calbiochem, 15 µm), Palbociclib (MedChemExpress, 100 nm), Dynasore (Sigma, 40 µm), and PD0325901 (Calbiochem, 1 µm). MK2206 (Selleckchem, 10 µm), Stattic (Selleckchem, 5 µm 30 min), Actinomycin D (Sigma, 10 nm), 5, 6‐dichloro‐1‐β‐D‐ribofuranosylbenzimidazole (DRB) 50 µM, Carbenoxolone (Sigma, 16–200 µm), 18beta‐Glucyrrhetinic acid (Sigma, 10 µm), Valproic Acid (Sigma, 1 mm).

### Generation of Crispr‐Cas9 Connexins 26 and Connexins 31 KO Clones

A stable clone of HaCat cells constitutively expressing SpCas9 was first generated. Cells were first reverse transduced with lentiCas9‐Blast lentivirus (Addgene #52962‐LV) at an MOI of 1 in the presence of 10 µg mL^−1^ polybrene. After 48 h, cells were selected with 5 µg mL^−1^ of blasticidin for 10 days. Stable cell clones were obtained from this pool by limiting dilution and screened by Western blotting with a monoclonal anti‐Flag antibody (clone M2, Sigma–Aldrich, cat. n. F1804). One clone (named 1–4) expressing high levels of Cas9‐Flag protein was selected for the following gene editing procedure (Table 2).

To generate single and double KO clones, one million Cas9‐expressing HaCaT cells were transfected by Nucleofection using the Nucleofector IIb device (program U‐020) with sgRNAs targeting either gene and then subcloned by limiting dilution. Protospacer sequences were as follows: GJB2, CCTCCTTTGCAGCCACAACG; GJB3, CCACCACGTATACCAGCACC. Synthetic sgRNAs were purchased from ThermoFisher (Invitrogen TrueGuide, cat. n. A35534).

Single and double KO clones of the GJB2 and GJB3 genes were identified by PCR and Sanger sequencing, followed by TIDE analysis.^[^ ^73^ ^]^ Primers were as follows: GJB2, Fwd: AGCATGCTTGCTTACCCAGAC; Rev: GCTAGCGACTGAGCCTTGAC. GJB3, Fwd: TGATTGTTATTATCAGCCAAAGCAT; Rev: TGGTGAGTACGATGCAGACG (Table 2). Clones harboring frameshift mutations predicted to abrogate protein production were selected for further experiments. Although mRNA transcripts may still be detected by qRT‐PCR, these clones are functionally null for Cx31 due to the generation of out‐of‐frame transcripts incapable of producing protein.

### RNA Interference

RNA interference was performed using Lipofectamine RNAimax (Invitrogen, Cat# 13778150) according to the manufacturer's protocol. Cells were transfected with siRNA (50 nm oligos) in 2 cycles. The first cycle of interference (reverse transfection) was performed on cells in suspension. The day after, a second cycle of interference (forward transfection) was performed on cells in adhesion. The siRNAs used for knocking down specific genes are reported reagents’ table. For each RNA interference experiment, a negative control was performed with the same amounts of scrambled siRNAs. Silencing efficiency was controlled by qRT‐PCR or Western Blot.

Oligo brand and sequence:

**Table:** 

RTN3 Stealth Invitrogen	5“‐CCCUGAAACUCAUUAUUCGUCUCUU‐3”
RTN4 Stealth Invitrogen	5′‐GGCGCCUCUUCUUAGUUGAUGAUUU‐3′
Clathrin Heavy Chain Invitrogen	5“‐GAAGAACUCUUUGCCCGGAAAUUUA‐3”

### Quantitative RT‐PCR Analysis

Quantitative RT‐PCR analysis was performed as previously described.^[^ ^3^ ^]^ Total RNA was extracted using the RNeasy Mini kit (Qiagen) and quantified by NanoDrop to assess both concentration and quality of the samples. 1 microgram of RNA was retrotranscribed using “High‐Capacity cDNA Reverse Transcription Kit – Thermofisher.” RT minus was made to check the absence of genomic DNA.

For gene expression analysis, 5 ng of cDNA was amplified (in triplicate) in a reaction volume of 10 µL containing the following reagents: 5ul of “TaqMan Fast Advanced Master Mix, Thermofisher,” 0.5 µL of “TaqMan Gene expression assay 20x, Thermofisher” (for details see the list below). Real‐time PCR was carried out on the QuantStudio12K Flex (Thermofisher), using a pre‐PCR step of 20s at 95 °C, followed by 40 cycles of 1s at 95 °C and 20s at 60 °C. Samples were amplified with primers and probes for each target, and for all the targets, one NTC sample was run.

Raw data (Ct) were analyzed with “Biogazelle qbase plus” software, and the fold change was expressed as CNRQ (Calibrated Normalized Relative Quantity) with Standard Error (SE). GeNorm Software chose Gapdh and 18s as the best housekeeping genes, and the geometric mean of Gapdh and 18s was used to normalize the data. The qPCR was performed by the qPCR‐Service at Cogentech‐Milano.

GJA1 assay ID: mm00439105_m1.

GJB2 assay ID: Hs00955889_m1.

GJB3 assay ID: Hs02378125_s1.

GJB4 assay ID: Hs00920816_s1.

GJB5 assay ID: Hs01921450_s1.

GJB6assay ID: Hs00922742_s1.

GJC1 assay ID: Hs00271416_s1.

RTN3 assay ID: hs01581961_g1.

RTN4 assay ID: hs01103689_m1.

GAPDH assay ID: Hs99999905_m1.

18s assay ID: Hs99999901_s1.

HPRT assay ID: Hs99999909_m1.

### Immunoblotting

Cells were washed twice with cold phosphate‐buffered saline (PBS), and lysed in JS buffer supplemented with proteases and phosphatases inhibitors (50 mm HEPES pH 7.5, 50 mm NaCl, 1% glycerol, 1% Triton X‐100, 1.5 mm MgCl_2_; 5 mm EGTA, protease inhibitor cocktail (Roche Basel, Switzerland), 20 mm Na pyrophosphate pH 7.5, 50 mm NaF, 0.5 m NaVO_4_ in HEPES pH 7.5 to inhibit phosphatases). Lysates were clarified by centrifugation at 13 000 r.p.m. for 30 min at 4 °C. Protein concentration was quantified by the Bradford colorimetric protein assay. The same amount of protein lysates was run on gradient 4–20% pre‐cast Gels (Bis‐Tris Bolt, Invitrogen) and transferred onto Protran Nitrocellulose Transfer membranes (Amersham). Membranes were then blocked with 1XTBS/0.1% Tween/5% milk for antibodies recognizing the total proteins or in 1XTBS/0.1% Tween/5% bovine serum albumin (BSA) for antibodies recognizing phosphorylated proteins before incubation with primary antibody according to the datasheet. Following TBS‐T washes, membranes were then incubated with the corresponding secondary antibody conjugated with horseradish peroxidase. After additional washes, the signal was detected at iBright (ThermoFisher Scientific) using ECL western blotting detection reagents (GE Healthcare) or ECL picoplus (ThermoFisher Scientific) (**Table** **3**).

**Table 3:** Antibodies and other reagents.

Antibody	Producer	Clone or Epitope	Catalog Number	Usage	Dilution or concentration
Clathrin Heavy Chain	BD	Clone 23	610499	WB	1:1000
RTN3	Homemade	aa 1–47, common to all RTN3 isoforms		WB	1.5 µg mL^−1^
RTN4	Novus		NB100‐5668155	WB	1:500
EGFR					
pY992 EGFR	Cell Signaling	Tyr 992	#2235	WB	1:1000
ERK1/2	Sigma	ERK‐1, 351–368	M7927	WB	1:5000
pERK1/2	Cell Signaling	Thr202/Tyr204	#9106	WB	1:1000
AKT	Cell Signaling		#9272	WB	1:1000
pAKT	Cell Signaling	Thr308	#9275	WB	1:500
STAT3	Cell Signaling		9139	WB	1:500
pSTAT3	BD	Y705	612 356	WB	1:500
Vinculin	Sigma		V9131	WB	1:5000
Tubulin	Sigma		T5168	WB	1:5000
E‐Cadherin	BD Transduction Laboratories		610 181	WB/IF	1:1000/1:200
P‐Cadherin	BD Transduction Laboratories		610 227	WB	1:1000
N‐Cadherin	BD Transduction Laboratories		610 920	WB	1:1000
β−Catenin	BD Transduction Laboratories		61 053	WB/IF	1:1000/1:500
FITC‐conjugated Phalloidin	Sigma–Aldrich		#P5282	IF	1:100
Secondary Antibody (Goat Anti‐Rabbit Antibody Conjugated to Horseradish Peroxidase)	Cell Signaling Technology		#7074	WB	1:5000
Secondary Antibody (Goat Anti‐Mouse Antibody Conjugated to Horseradish Peroxidase)	Cell Signaling Technology		#7076	WB	1:5000
Donkey anti‐Mouse IgG (H + L) Highly Cross‐Adsorbed Secondary Antibody, Alexa Fluor 488	Thermofisher		A21202	IF	1:400
Cy3 AffiniPure Donkey Anti‐Rabbit IgG (H + L)	Jackson ImmunoResearch		Cat# 711‐165‐152	IF	1:400
DAPI	Thermofisher		#D‐1306	IF	1:5000
SPY595‐DNA	Tebubio		SC30	Live Imaging	1:1000
Calcein‐AM	Thermofisher scientific		C3100MP	Live Imaging	1:500

### RNA‐Sequencing

RNA was isolated from three biological replicates of cells seeded in 6‐well plates and treated or not with 100 ng mL^−1^ EGF for 24 h. Cells were washed with cold PBS, followed by RNA extraction using the RNeasy Mini kit (Qiagen) as per the manufacturer's instructions. Total RNA concentration was measured with Nanodrop, and samples were normalized by diluting with RNAse‐free water. Sample quality was verified using Agilent Bioanalyzer 2100, and final concentrations were measured using Qubit/Quant‐IT Fluorometric Quantitation (Life Technologies). Illumina stranded total RNA prep library was prepared using 100 ng of RNA as per the manufacturer's instructions (Illumina Stranded mRNA Preparation and Ligation kit, (Illumina) and sequenced with Novaseq 6000 (S4 instrument, v1.5 (Illumina), 2 × 50 bp.

Reads were aligned to the GRCh38/hg38 assembly human reference genome using the STAR aligner (v 2.6.1d)(1)^[^ ^74^ ^]^ and quantified using Salmon (v1.4.0).^[^ ^75^ ^]^ Differential gene expression analysis was performed using the Bioconductor package DESeq2 (v1.30.0),^[^ ^37^ ^]^ which estimates variance‐mean dependence in count data from high‐throughput sequencing data and tests for differential expression exploiting a negative binomial distribution‐based model.

### Cell Streaming Assay

Cells were seeded in twelve‐well plates (500 000 × 10^6^ cells per well) in complete medium and cultured until a uniform monolayer had formed. In order to induce serum starvation, complete medium was replaced with serum‐free medium for 48 h. Then, before initiating live imaging, the medium was refreshed, and cells were treated with EGF with or without various drugs and inhibitors. Spinning disk Inverted microscope (Olympus) equipped with an IX83 inverted microscope (10 × objective), provided with an IXON 897 Ultra camera (Andor), was used to take pictures every 5 min over a 48 h period. The assay was performed using an environmental microscope incubator (OKOlab) set to 37 °C and 5% CO_2_ perfusion.

Time‐lapse imaging was initiated at the time of EGF stimulation (*t* = 0). Although pre‐stimulation frames were not plotted, non‐EGF‐treated monolayers were cultured and imaged in parallel as static controls. These –EGF monolayers consistently exhibited no detectable collective motility and served as reference baselines for EGF‐induced transitions.

### Wetting Assay

Cells were seeded in an ultra‐low attachment round‐bottom 96‐well plate (Costar) to allow the formation of spheroids. The following day, spheroids were transferred to a 6‐well plate previously coated with 10 µg mL^−1^ Fibronectin (Merck Life Science) (diluted in PBS, incubated overnight at +4 °C, and washed twice with PBS). Spheroids were monitored as they wet the substrate by time‐lapse imaging for 48 h Ix83 inverted microscope ( IXplore (Evident)) equipped with an ORCA Flash 4 camera (Hamamatsu) and driven by CellSens Dimension software 4.3 (Build 31056). Cells were imaged using a 4x/0.13 objective every 10 min over 48 h, maintaining the correct environmental conditions using a CellVivo incubator (Pecon).

Analysis of the spreading area over time was performed using ImageJ. The data were normalized to the area of the spheroid at time 0 h. To evaluate the impact of GJ communication, spheroids were treated with EGF in the presence of GJ blocker (Carbenoxolone) before starting the initiation of the spreading.

### Analysis of Collective Motility, PIV

L_CORR_ was computed from V_PIV_ velocity fields derived from time‐lapse image sequences using a standard cross‐correlation‐based PIV algorithm. V_RMS_ and V_RMS‐CM_ were similarly calculated from sequential frames (time‐lapse), where V_RMS_ reflects local instantaneous cell motility and V_RMS_CM_ captures the velocity of the center of mass of each cell (V_RMS‐CM_) that provides insights into local fluctuations in cell velocity.

Maps of the flow field in the cell monolayer were obtained by analyzing phase‐contrast movies using particle image velocimetry (PIV). A custom MATLAB script was employed that utilizes the PIVLab software (1) or uses the MATLAB MPIV toolbox (https://www.oceanwave.jp/softwares/mpiv/index.php). The interrogation window size was typically 50 × 50 µm, with a 50% overlap between adjacent windows. This size was chosen to ensure that each interrogation window contained ≈5 cells. To verify the robustness of the results, larger and smaller interrogation windows were tested, which yielded consistent outcomes. For each monolayer, time‐lapse images from at least four different fields of view were collected simultaneously. The time evolution of motility parameters was calculated separately for each field of view, and the results were then averaged at each time point over all fields of view of the same cell type. PIV analysis provides the instantaneous velocity v_i_ in each interrogation window i. From this velocity map, the instantaneous velocity of the center of mass at a given time t was computed as the spatial average:

(1) $$V_{\mathrm{cm}}\left(t\right)=\langle v_{i}\left(t\right)\rangle I$$

where [[⟨…⟩]]_i denotes the average over all the interrogation windows.

The velocity fluctuations were evaluated using the root mean square velocity:

(2)

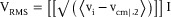




The order parameter ψ, which quantifies the degree of alignment in the velocity field, was computed as:

(3) $$\psi =\left|{\left\langle v_{i\;}\right\rangle }_{i\;}\right|/\surd \left({\left\langle {\left|v_{i}\right|}^{2}\right\rangle }_{i}\right)$$

This definition ensures that ψ = 1 only when all cells move with the same speed and in the same direction (perfectly uniform motion). Conversely, ψ≅0 is expected when velocities are randomly oriented, indicating a disordered motion.

By analyzing the time evolution of V_cm_ and v_RMS_, the peak of maximum motility was identified. A time window centered around this peak was selected to compute the spatial velocity correlation function, restricting the analysis to this interval. By looking at the time evolution of the two velocities V_cm and v_rms, the peak of maximum motility was identified, and a time window centered around the peak was then selected and perform the calculation of the spatial velocity correlation over the restricted time interval for each sample. The spatial velocity correlation function was computed using the Fourier Transform method applied to the velocity field components. Given a velocity field v(x,t) = (v_x_ (x,t),v_y_ (x,t)), the correlation function C_vv_ (r) was obtained as:

(4) $$C_{\mathrm{vv}}\left(r\right)=F^{\left(-1\right)}\left({\left|F\left(v_{x}\right)\right|}^{2}+{\left|F\left(v_{y}\right)\right|}^{2}\right)$$

where F and F^(‐1)^ denote the Fourier and inverse Fourier transforms, respectively. The correlation function was then averaged over all frames within the selected time window.

Since the velocity field may exhibit nonzero mean values due to the presence of coherent motion, C_vv_ (r) was corrected by subtracting the mean velocity variance:
(5)

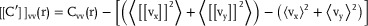




The correlation length L_CORR_ of the velocity field was estimated by fitting the corrected correlation function $[\left[C^{\prime }\right]]\_\mathrm{vv}$ (r) to a stretched exponential function of the form

(6) $$e^{\left(-\left[\left[\left(r/L_{\mathrm{CORR}}\right)\right]\right]\right.}$$

where γ is the stretching exponent. The correlation length L_CORR_ provides a measure of how far velocity fluctuations are spatially correlated. A larger L_CORR_ indicates that cells move in a more coordinated manner over longer distances, whereas a smaller L_CORR_ suggests more localized and independent motion.

### Definition of Dynamic Cell States

While the Order Parameter Ψ is a valuable metric to assess directional alignment, L_CORR_ was also relied upon as a robust, spatially resolved measure of coordinated motion. In the system, flocking was operationally defined as the coexistence of elevated V_RMS_ and extended L_CORR_, which together signify both activation and spatial alignment of collective migration. Importantly, the core mechanistic findings on EGF‐induced unjamming remain valid independently of the presence of flocking, allowing us to generalize the conclusions across both disordered and aligned collective behaviors.

To ensure consistent interpretation of the observed collective behaviors, tissue states were classified according to a combination of physical metrics.

A *jammed solid* was defined by V_RMS_ ≈0, low L_CORR_, and minimal shape deformation or neighbor exchange.

An *unjammed, disordered fluid* exhibited elevated V_RMS_ but low L_CORR_.

A *flocking fluid* combined high V_RMS_ with extended L_CORR_, indicating directional coordination.

Finally, a *flocking solid* maintained alignment (high L_CORR_) and low shape index variability but exhibited moderate neighbor exchange, indicating structural reconfiguration under alignment constraints.

### Cell Tracking FUCCI

To measure the number of cells over time in a different cell cycle phase was used Fiji Trackmate plugin^[^ ^1^, ^76^ ^]^ was used, by analysing the green and red channels separately. Ratio of cells in G2/M is then calculated as the number of green nuclei/(Red Nuclei + Green Nuclei) at each time point.

### Immunofluorescence

Cells cultured in iBidi chambers were washed with 1XPBS, fixed with 4% Paraformaldehyde for 10 min, and permeabilized with 0.1%Triton X‐100 and 10% FBS for 15 min before incubation with the primary antibodies for 2 h at room temperature. Cells were then washed with 1XPBS and incubated with secondary antibodies for 1 h at room temperature. After an additional 1XPBS wash, nuclei were stained with DAPI diluted in 1XPBS for 15 min. Samples were conserved in anti‐fade mounting medium (Vectashield) for confocal imaging. Antibodies were diluted in 1X PBS with 10% FBS and 0.1% Triton X‐100.

### Image Acquisition

Confocal microscopy was performed using a Leica TCS SP8 laser confocal scanner mounted on a Leica DMi8 microscope; a HC PL APO CS2 63 × oil immersion objective was used. The software used for all acquisitions was Leica LAS AF (Laser lines: 405, 488, and 561 nm). For the analysis of E‐Cadherin and β‐Catenin immunostaining, z‐stack (x2.5 optical zoom, 0.5 µm) were acquired and the focal plane was kept at 3 µm distance starting from the bottom for each z‐stack acquired.

### Cell Shape and Cell Volume Estimation

Cells were cultured in iBidi chambers until confluency, serum starved for 48 h, then treated with EGF with or without Carbenoxolone. Then, cells were washed with 1XPBS and fixed with 4% Paraformaldehyde. After washing twice with 1XPBS, Cells were permeabilized for 10 min with 0.1%Triton X‐100 and incubated first with FITC‐Phalloidin for 1 h for Actin staining. After additional PBS washing, nuclei were stained with DAPI diluted in 1xPBS for 15 min. Samples were conserved in anti‐fade mounting medium (Vectashield) for confocal imaging.

For 2D cell shape analysis, confocal images of HaCaT monolayers were acquired using a Leica TCS SP8 laser confocal scanner mounted on a Leica DMi8 microscope; a HC PL APO CS2 40 × oil immersion objective was used. For each field of view, fluorescent nuclei and Phalloidin channels were acquired. Images were processed using Cellpose software^[^ ^77^ ^]^ and cells were segmented using the Cytoplasm 2.0 model. Morphological parameters were then extracted using Fiji.

For cell volume estimation, confocal images of HaCaT monolayers were acquired using a Leica TCS SP8 laser confocal scanner mounted on a Leica DMi8 microscope equipped with a motorized stage (oil immersion 63 × objective, x2.5 optical zoom, 0.3 mm z‐stack). Cell volume and cell shape in 3D were analyzed with ARIVIS (Arivis Vision4D, v4.1.2) using a custom pipeline. For the nuclei segmentation, the Intensity Threshold Segmenter operation, instead of the cells, uses the Membrane‐based Segmenter.

### Cell Shape Segmentation and Tracking over Time

For cell shape segmentation and tracking, HaCaT Ecad‐EGFP cells were used to monitor cell shape using E‐Cadherin signal over time. Cells were seeded in glass‐bottomed dishes (NUNC, 27 mm, 1.5 × 10^6^ cells per well) and treated as previously described. Images were acquired using a spinning disk confocal microscope (Olympus) equipped with an IX83 inverted microscope provided with an IXON 897 Ultra camera (Andor).

The E‐cadherin signal (3 different Z planes spaced 3 mm apart) was recorded every 10 min over a 24 h period using a 20x/0.75 objective. The assay was performed using an environmental microscope incubator (OKOlab) set to 37 °C and 5% CO_2_ perfusion.

Image analysis was performed using two custom‐developed Fiji^[^ ^78^ ^]^ plugins. After z‐stack maximum projection, cell detection was carried out using CellPose software^[^ ^77^ ^]^ with the Cytoplasm model. The resulting labels (cell detections) were filtered using the MorpholibJ^[^ ^79^ ^]^ “label size filtering” module, retaining only those larger than 10 µm^2^.

Cell tracking was then performed using the Label‐Image‐Detector of Fiji Trackmate,^[^ ^76^ ^]^ with the following parameters: “simplify contours” was set to false, gap closing was allowed for a maximum of 3 frames and 25 µm, respectively, and frame‐to‐frame linking was set with a maximum distance of 30 µm. Track merging and splitting were not permitted. For all cells/spots at all time points, the ellipse aspect ratio for each cell (spot) was calculated using TrackMate's “Spot Morphology Analyzer Provider.” Further data analysis was conducted with Python. For each track found, the spot/cell ellipse aspect ratio at any given time was normalized by dividing it by the value calculated at the first available time point. Finally, for each time point, the mean normalized ellipse aspect ratio was calculated and plotted.

### Density Fluctuations and Flocking Behavior

HaCaT H2B‐mCherry and HaCaT Fucci cells were used for nuclei tracking and segmentation. VFC T1 and T3 cells were incubated with SPY595‐DNA for 1 h before initiating the live imaging. Cells were cultured, treated, and processed as for the cell streaming assay. The cells in the movies were first segmented with Cellpose 2.0 software.^[^ ^77^ ^]^ Then the segmented cells were tracked with the plugin of ImageJ, i.e., TrackMate. After that, the tracked data was analyzed by MATLAB R2020b.

To find the neighbors of reference cells, the radial distribution function (RDF) of different samples were first calculated. The distance of the first valley of the RDF was defined as the threshold neighbor distance. The cells whose distance from the reference cell and themselves was smaller than this threshold neighbor distance are defined as the neighbors of the reference cell. The average distance between cell *i* and its neighbors shown in Figure 6F is defined as 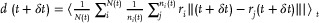, where *δt* is time delay, *N*(*t*) is the total number of cells in the field of view at time *t*, *n_i_* (*t*) is the number of neighbors of cell *i* at *t*, *r_i_* (*t*+*δt*) and *r_j_* (*t*+*δt*) are the coordinates of cell *i* and cell *j* at time *t*+*δt*.

### Density Fluctuations and Flocking Behavior—About the Probability Movies

The probability density distribution shown in the movies characterize the probability of finding the same tagged cell at the location (*x*,*y*) away from a reference cell at different time delays, knowing that it started as a neighbor of the reference cell at *δt* = 0, is defined as $P\;\left(r,\delta t\right)={\left\langle \frac{1}{N(t)}\sum_{i}^{N(t)}\frac{1}{n_{i}\left(t\right)}\sum_{j}^{n_{i}\left(t\right)}\Delta \left(r_{ij}\left(t+\delta t\right)-r_{ij}\left(t\right)\right)\right\rangle }_{t}\;$, where *δt* is the time delay, *N*(*t*) is the total number of cells in the field of view at *t*, *n_i_* (*t*) is the number of neighbors of cell *i* at *t*, *r_ij_* (*t*) is the distance between cell *i* and cell *j* at time *t*.

### Fluorescence Recovery after Photobleaching (FRAP)

Cells were loaded with Calcein‐AM (20 µm, Thermofisher Scientific) for 45 min at 37C. For FRAP measurement, images were recorded using a Confocal Spinning Disk microscope (Olympus) equipped with an IX83 inverted microscope provided with an IXON 897 Ultra camera (Andor), using a 20x/0.75 objective and maintaining the environmental microscope incubator (OKOlab) set to 37 °C and 5% CO2 perfusion.

A specific area of the monolayer (95 mm diameter) was bleached using the cellFRAP/IX3FRAP module with a 405 nm laser at maximum power. The recovery of Calcein‐AM signal was then monitored over 10–30 min post‐bleaching. Image analysis was performed using a custom‐developed Fiji^[^ ^1^ ^]^ plugin. For each movie, the bleached area was automatically detected by sequentially performing the following steps: isolating the bleach frame, applying a median filter (radius 10), inverting the image, and applying a very high intensity threshold (greater than or equal to 65200). Regions were then detected using the Fiji Analyze Particles tool, with an area range between 4500 and 2000 µm^2^. Typically, only one region was detected with these parameters; if multiple regions were detected, user supervision was required. A circle was fitted to each detected region, and the area was shrunk by 20% to avoid measuring intensities from partially bleached cells. The mean intensity value in the bleached area was then measured, corrected for background and acquisition photobleaching, and the intensity curves were normalized to the pre‐bleaching mean intensity values.^[^ ^80^ ^]^

### GJ Intercellular Communication Assay (Parachut Assay)

Gap junction coupling was assessed using a modified parachute dye‐transfer assay.^[^ ^81^ ^]^ Donor cells (HaCaT, Cx26, or Cx31 CRISPR KO or double KO) were double‐labeled by loading with Calcein‐AM (Invitrogen; 5 µm, 30 min, 37 °C) and staining with the lipophilic membrane dye PKH26 (Sigma; according to manufacturer's protocol). After thorough washing to remove excess dye, donor cells were detached, counted, and overlaid (“parachuted”) at a 1:100 ratio onto receiving monolayers of the indicated cell lines followed by far‐red Nuclight‐labeling (Sartorius). After 4 h co‐incubation at 37 °C, gap junctional transfer was evaluated by fluorescence microscopy (Confocal Spinning Disk microscope (Olympus) provided with an IXON 897 Ultra camera (Andor), 40X objective). Successful coupling was scored as the appearance of Calcein fluorescence in PKH26‐negative acceptor cells.

### Electron Microscopy

Electron microscopic examination was performed as previously described (Beznoussenko et al., 2015). A brief description of the process is presented below. Embedding. HaCaT cells were grown on MatTek glass bottom dishes (MatTek Corporation, USA) were fixed with of 2,5% paraformaldehyde and 2,5% glutaraldehyde (EMS, USA) in 0.1 m sodium cacodylate, pH 7.4, for 2 h at RT, followed by 6 washes in 0.2 m sodium cacodylate, pH 7.2, at RT. Then cells were incubated in a 1:1 mixture of 2% osmium tetroxide (OsO4) and 3% potassium ferrocyanide for 1 h at RT, followed by 6 times rinsing in cacodylate buffer pH 6.9. Then the samples were sequentially treated with 0.3% thiocarbohydrazide in 0.2 m cacodylate buffer for 10 min and 1% OsO4 in 0.2 m cacodylate buffer, pH 6,9, for 30 min. Then, samples were rinsed with 0.1 m sodium cacodylate buffer pH 6,9 until all traces of the yellow osmium fixative had been removed, washed in de‐ionized water, treated with 1% uranyl acetate in water for 1 h, and washed in water again.^[^ ^82^ ^]^ The samples were subsequently subjected to dehydration in ethanol and embedded in Epoxy resin at RT and polymerized for at least 72 h in a 60 °C oven.

### Sectioning

Embedded samples were then sectioned with a diamond knife (Diatome, Switzerland) using Leica EM UC7 ultra microtome (Leica Microsystems, Vienna) and then cut the series of 70‐nm sections until the moment when, according to the estimation of the depth of the cell, the cell should be finished. Sections were analyzed with a Tecnai20 High Voltage EM (FEI, now Thermo Fisher Scientific; The Netherlands) operating at 200 kV.

### Statistical Analysis

Time‐lapse metrics (e.g., motility, alignment, intensity) were computed by first extracting per‐frame values from each ROI. For each time point, the mean value was calculated across pixels within each ROI and these means were then averaged across all ROIs from a given biological condition. This procedure was repeated across independent replicates, and the resulting time courses were plotted with mean ± SEM.

Replicates were defined as follows: sampling replicates refer to multiple ROIs from a single well; technical replicates are ROIs from different wells of the same plate; biological replicates consist of independent experiments performed on different days using newly seeded cultures. Unless otherwise stated, statistical comparisons were performed on values aggregated at the level of biological replicates. This approach is consistent with best practices described in Lord et al.^[^ ^83^ ^]^

All statistical analyses were performed using GraphPad Prism 10. Data are expressed as mean ± standard deviation (s.d.) unless otherwise indicated. The number of experiments/samples analyzed is indicated in the figure legends. When comparing two groups, statistical significance was calculated using a two‐tailed Student's *t*‐test or a non‐parametric two‐tailed Mann‐Whitney *t*‐test as indicated. One‐way ANOVA or Kruskal‐Wallis tests were used as reported for comparison of more groups. Significance is defined as ^∗^ *p*  <  0.05; ^∗∗^ *p*  <  0.01; ^∗∗∗^ *p*  <  0.001 and ^∗∗∗∗^ *p*  <  0.0001;ns‐Not Significant. Statistical test used and significance are indicated in Table 1.

## Conflict of Interest

The authors declare no conflict of interest.

## Supporting information



Supporting Information

Supplemental Movies

## Data Availability

The data that support the findings of this study are available from the corresponding author upon reasonable request.
