# Supplementary material for: De Novo Gene Transcription of Connexin Mediates Cytoplasmic Fluid Exchange and Flocking Transitions in Physiological and Cancerous Epithelial Systems
Source: Adv Sci (Weinh). 2025 Dec 23;13(6):e08648. doi: 10.1002/advs.202508648 (PMC12866692; doi:10.1002/advs.202508648)
Supplement: Supplementary file 1 — Supporting Information [file ADVS-13-e08648-s001.docx]

**Supplementary Material**

**Supplementary Figures:**


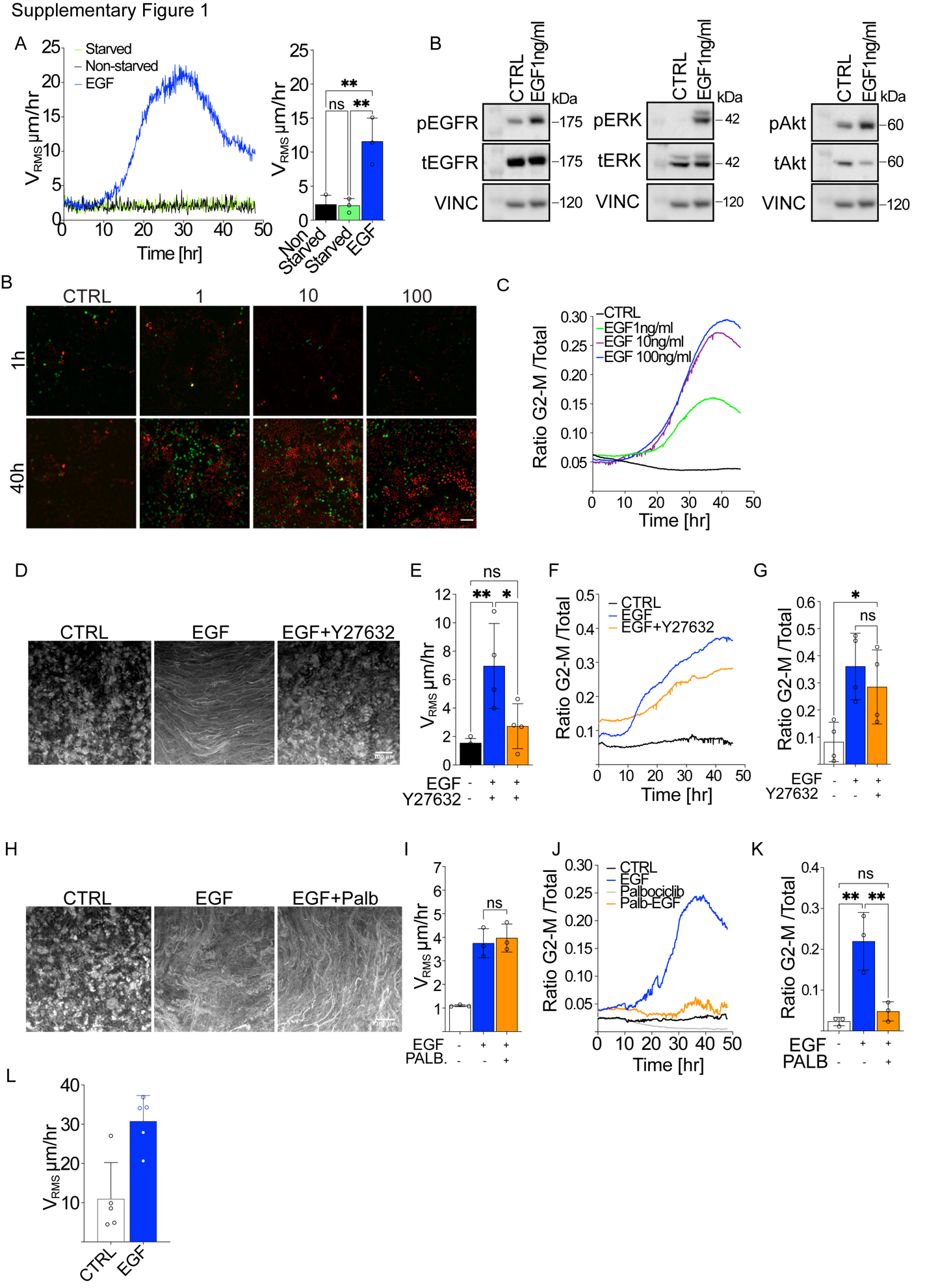


**Supplementary Figure 1. Response to EGF stimulation of epithelial cell ensembles and impact on cell proliferation**

**A.** Confluent HaCaT cells are cultured in complete culture medium (Non-Starved) or in Serum Free medium (Starved) for 48h or subsequently treated with EGF. Time evolution of the Root Mean Square Velocity (V_RMS_) measured by Particle velocimetry analysis (PIV) over 48h (left) and its mean within the 48h (right). (n=3 independent experiments, each including multiple fields of view (FOVs)). NS = Not significant, ** p = 0.006. One way Anova followed by Bonferroni multiple comparison test

These data confirm that non-starved HaCaT monolayers seeded at jamming density do not exhibit collective motility, even in the presence of ambient serum, whereas serum-starved monolayers display a dormant yet mechanically primed state that enables subsequent EGF-induced flocking. This operational baseline is consistent with prior reports demonstrating that serum withdrawal preserves contractile tension and mechanical coupling in quiescent keratinocyte monolayers, thereby allowing rapid emergence of coordinated migration upon growth factor re-stimulation [70] [71].

**B.** Expression and phosphorylation status of EGFR, ERK1/2 and AKT in HaCaT cells, stimulated with EGF (1 ng/ml) for 5 min and analyzed by WB. Vinculin was used as a loading control. Molecular weights are indicated on the right.

**C-D.** Analysis of cell cycle using Fucci construct. B) Snapshots of HaCaT monolayer engineered to express the Fucci construct after 1h and 40h following treatment with EGF (0 (CTRL), 1, 10, and 100 ng/ml). Representative images from n = 3-4 time-lapse series. Scale bar, 100 μm. **C)** Quantification of the fraction of cells in G2/M (green signal) over the total number of cells over 48h timelapse. (n = 3-4 independent experiment).

**E-H.** Analysis of monolayer dynamics and re-entry in cell cycle of HaCaT cells undergoing flocking motion in the presence of ROCK inhibitor Y27632 or DMSO (EGF). D) Representative phase contrast image of the maximum intensity projection (MIP) of all frames acquired over a 24-hour period (5min/frame) of quiescent HaCaT monolayers (Control) treated with EGF to induce flocking motion in the absence or the presence of the 15 mM Rock inhibitor Y27632. Representative images from n = 4 time-lapse series. Scale Bar, 100 μM. **E)** Mean of V_RMS_ calculated in the time frame of flocking motion. Data are mean ± SD (n = 4 independent experiment). **F)** Quantification of the time evolution of the ratio in G2/M (green signal) of Fucci-expressing HaCaT cells over the total number of cells over 48h timelapse after stimulation with EGF and treatment with the Rock Inhibitor, Y27632. **G)** Ratio of cells in G2/M at 40h following EGF treatment. Data are mean ± SD (n = 4 independent experiment).

**I-L.** Analysis of monolayer dynamics and re-entry in cell cycle of HaCaT cells undergoing flocking motion in the presence of Cdk4/6 inhibitor (Palbociclib 100nM) or DMSO (EGF). **H)** Representative phase contrast image of the maximum intensity projection (MIP) of all frames acquired over a 24-hour period (5min/frame) of quiescent HaCaT monolayers (Control) treated with EGF to induce flocking motion in the presence of the Cdk4/6 inhibitor (Palbociclib 100nM) or DMSO. Representative images from n = 3 time-lapse series. Scale Bar, 100 μM. **I)** Mean of V_RMS_ calculated in the time frame of flocking motion (Time frame 10-40 h after EGF stimulation). Data are mean ± SD (n = 3 independent experiment). **J)** Quantification of the time evolution of the ratio of cells in G2/M (green signal) over the total number of cells over 48h timelapse. **K)** The ratio of cells in G2/M at 40h following EGF treatment. Data are mean ± SD (n = 3 independent experiment).

**M.** Collective motility induced in Bronchial epithelial primary cultures in Air-Liquid interface (ALI) upon treatment with EGF. Mean overtime of total cell motility (V_RMS_) by the analysis of Particle Image Velocity (PIV). n = 5 time-lapse series from one healthy donor. Scale bar 100μM.

Statistical tests and significance are indicated in Table 1.


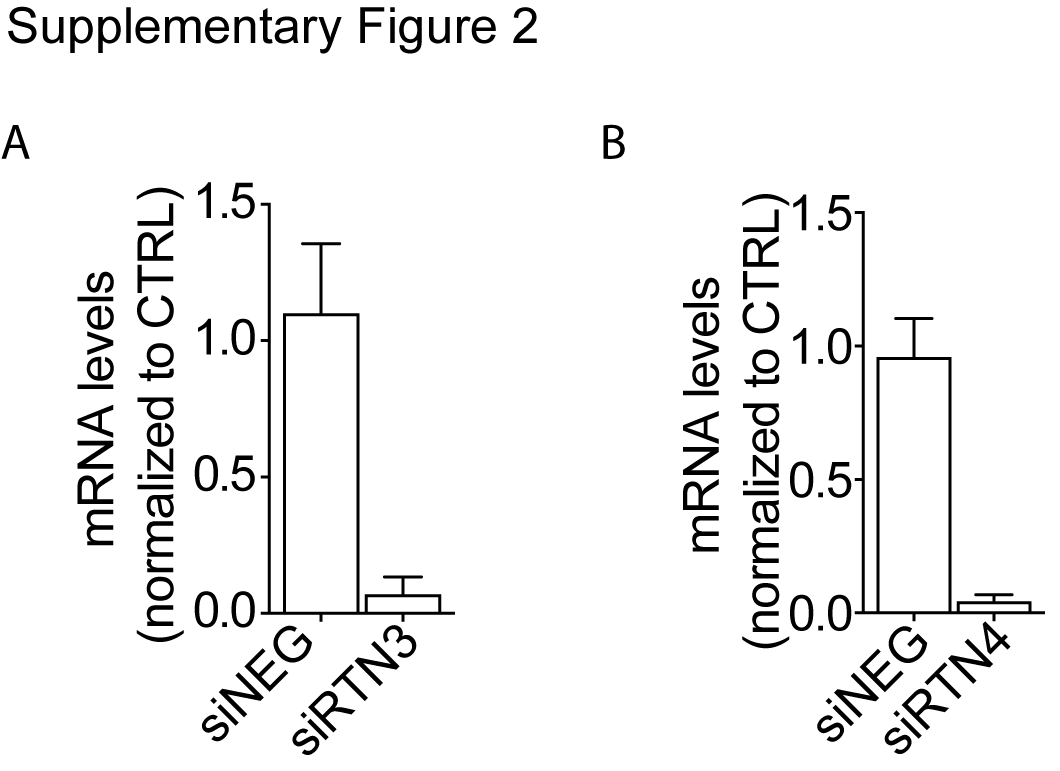


**Supplementary Figure 2. Efficacy of RTN3 and RTN4 silencing**

HaCaT cells were transfected with scrambled siRNA control (siNEG) or siRNA oligo against RTN3 or RTN4. Silenced cells were serum straved before addition of EGF to induce flocking motion. The efficacy of RTN3 (A) and RTN4 (B) silencing was measured by qRT-PCR (mRNA fold increase relative to the levels of control cells after normalizing for GAPDH and 18S mRNA levels) (Related to Figure 2A-C). Data are the mean ± SD (n = 2-3 independent experiments).


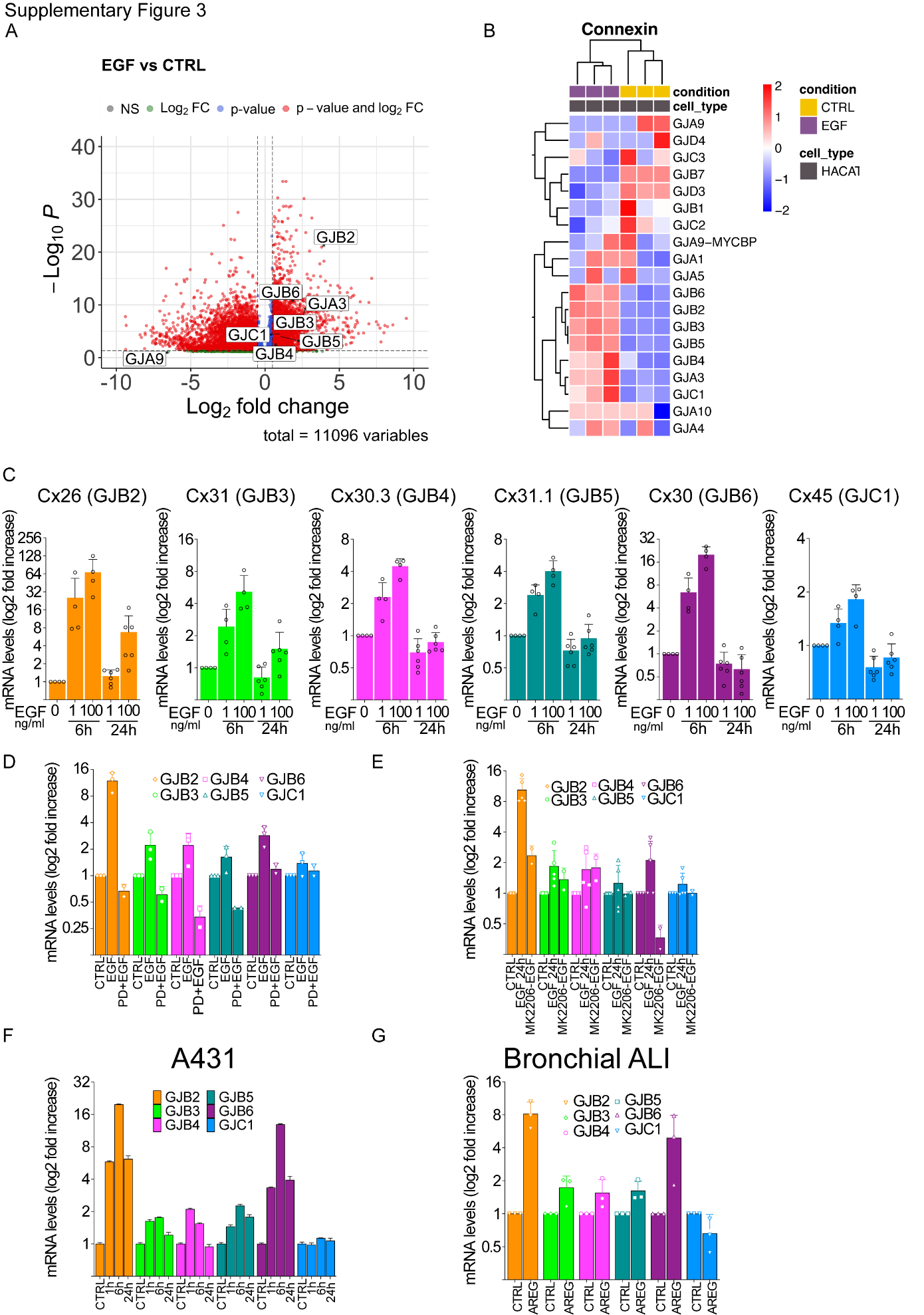


**Supplementary Figure 3. Regulation of Connexins transcript in EGF-treated HaCaT and A431 cells and human Bronchial epithelia explants**

**A-B.** RNAseq analysis of HaCaT cells serum starved for 48h then treated or not with EGF (100ng/ml) for 24h (n=3 independent experiments). **A)** Volcano plot depicting connexin genes. The y axis indicates the −log10 P value, whereas the x axis (log2 fold change) indicates gene expression. Each dot represents a gene. **B)** Heatmap showing connexin genes normalized count RPKM. **C)** mRNA levels of Connexins treated with low (1ng/ml) or high (100ng/ml) doses of EGF for 6h and 24h. Data are the mRNA fold increase relative to the levels of control cells at T0h after normalizing for GAPDH and 18S mRNA levels (n=4-6 independent experiments).

**D-E**. mRNA levels of Connexins genes in HaCaT cells treated with EGF for 24h in the absence or presence of the ERK1/2 inhibitor, PD02501 (D), or the AKT inhibitor, MK2206 (E), quantified by qRT-PCR. Data are the mRNA fold increase relative to the levels of control cells at T0h after normalizing for for GAPDH and 18S mRNA levels (n = 3 independent experiments).

**F.** mRNA levels of Connexins genes in A431 cells treated with EGF for 0h ,1h, 6h, and 24h; quantified by qRT-PCR. Data are the mRNA fold increase relative to the levels of control cells at T0h after normalizing for GAPDH and 18S mRNA levels (n = 3 independent experiments).

**G.** mRNA levels of Connexins genes in human bronchial epithelial explants grown as Air-Liquid Interface cultures cells treated with EGF for 72h; quantified by qRT-PCR. Data are the mRNA fold increase relative to the levels of control cells at T0h after normalizing for GAPDH and 18S mRNA levels (n=3 independent experiments).


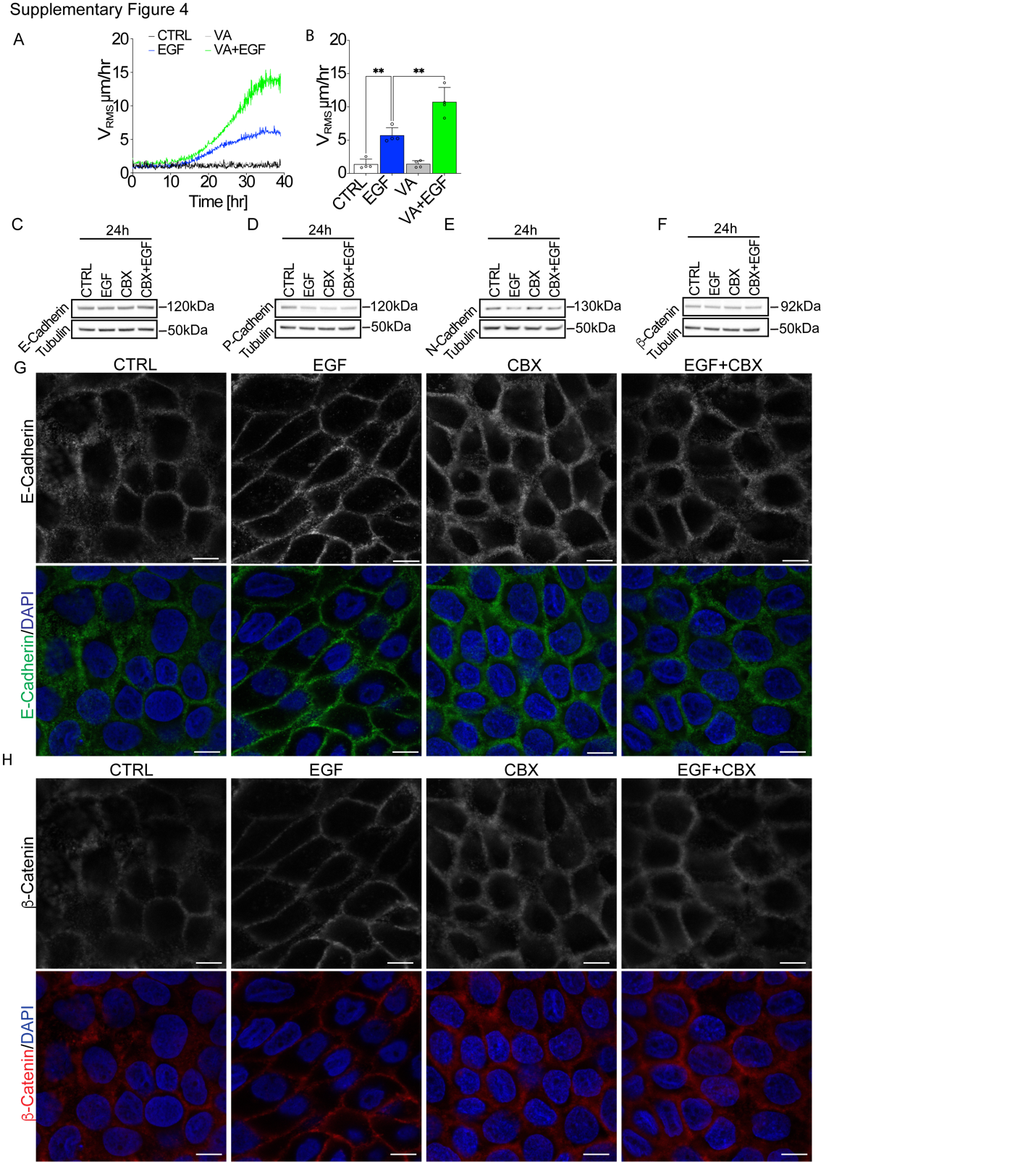


**Supplementary Figure 4. Gap Junction blocker Carbenoxolone does not impact Cell junctions expression and organization**

**A-B**. HaCaT cells are cultured to confluency, serum starved and treated with EGF 100ng/ml in the absence or in the presence of Valproic Acid (VA 1mM). Root Mean Square Velocity (V_RMS_) measured by Particle velocimetry analysis (PIV) over 48h (left) and its mean within cell flocking framecut (right) in control or in cells treated EGf withor without Connexin activator. V_RMS_ is expressed as the mean ± SD (n = 4 independent experiments).

**C-F.** Expression levels of E-Cadherin (C), P-Cadherin (D), N-Cadherin (E) and β-Catenin (F) in HaCaT cells, stimulated with EGF (100 ng/ml) for 24h in the presence or in the absence of Carbenoxolone (CBX, 16μm) and analyzed by WB. Vinculin was used as a loading control. Molecular weights are indicated on the right.

**G-H** Expression and organization of E-Cadherin (G) and β-Catenin (H) detected by immunofluorescence in HaCaT cells, stimulated with EGF (100ng/ml) for 24h in the presence or in the absence of Carbenoxolone (CBX, 16μm). Scale bar 10μm.


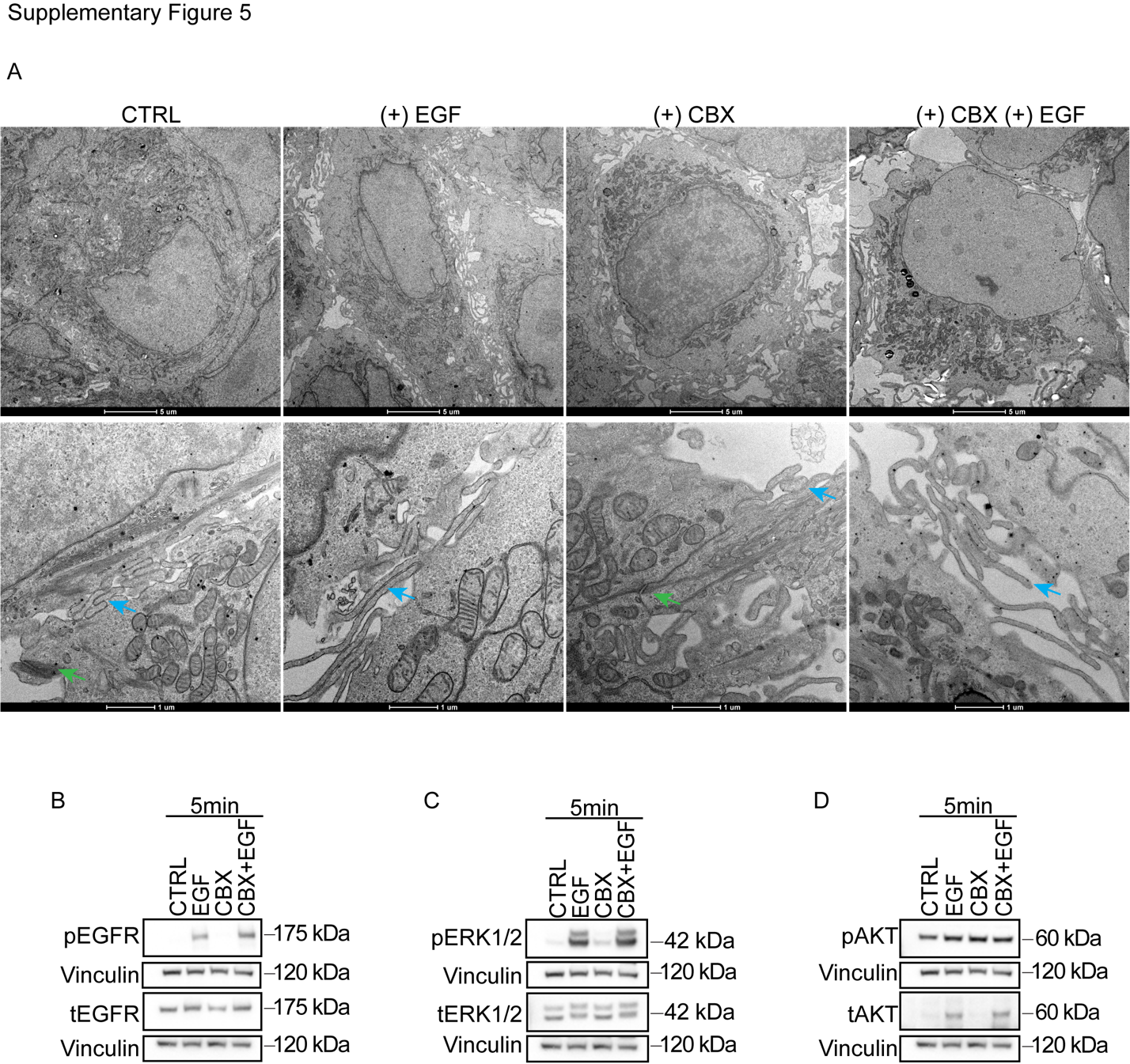


**Supplementary Figure 5. Gap Junction blocker Carbenoxolone does not impact Cells morphology and EGF signalling pathways**

**A.** Ultrastructural Electron Microscopy of HaCaT monolayers, serum starved and treated with EGF 100ng/ml in the absence or in the presence of Carbenoxolone (CBX 16uM) for 24h. Higher panels: An elongated shape of cells is observed after (+EGF) treatment. Lower panels: Cell-to-cell attachments are variable and formed by multiple interdigitations (blue arrows) of the plasma membranes of adjacent cells and cell junctions (mostly desmosomes (green arrows)) in both control (CTRL) and (+CBX) treatment. Elongated microvilli are present after (+EGF) and (+CBX, +EGF) treatments. Scale bars are indicated on the images.

**B-D** Expression and phosphorylation status of EGFR, ERK1/2 and AKT in HaCaT cells, stimulated with EGF (100 ng/ml) for 5 min in the presence or in the absence of Carbenoxolone (CBX, 16mm) and analyzed by WB. Vinculin was used as a loading control. Molecular weights are indicated on the right.


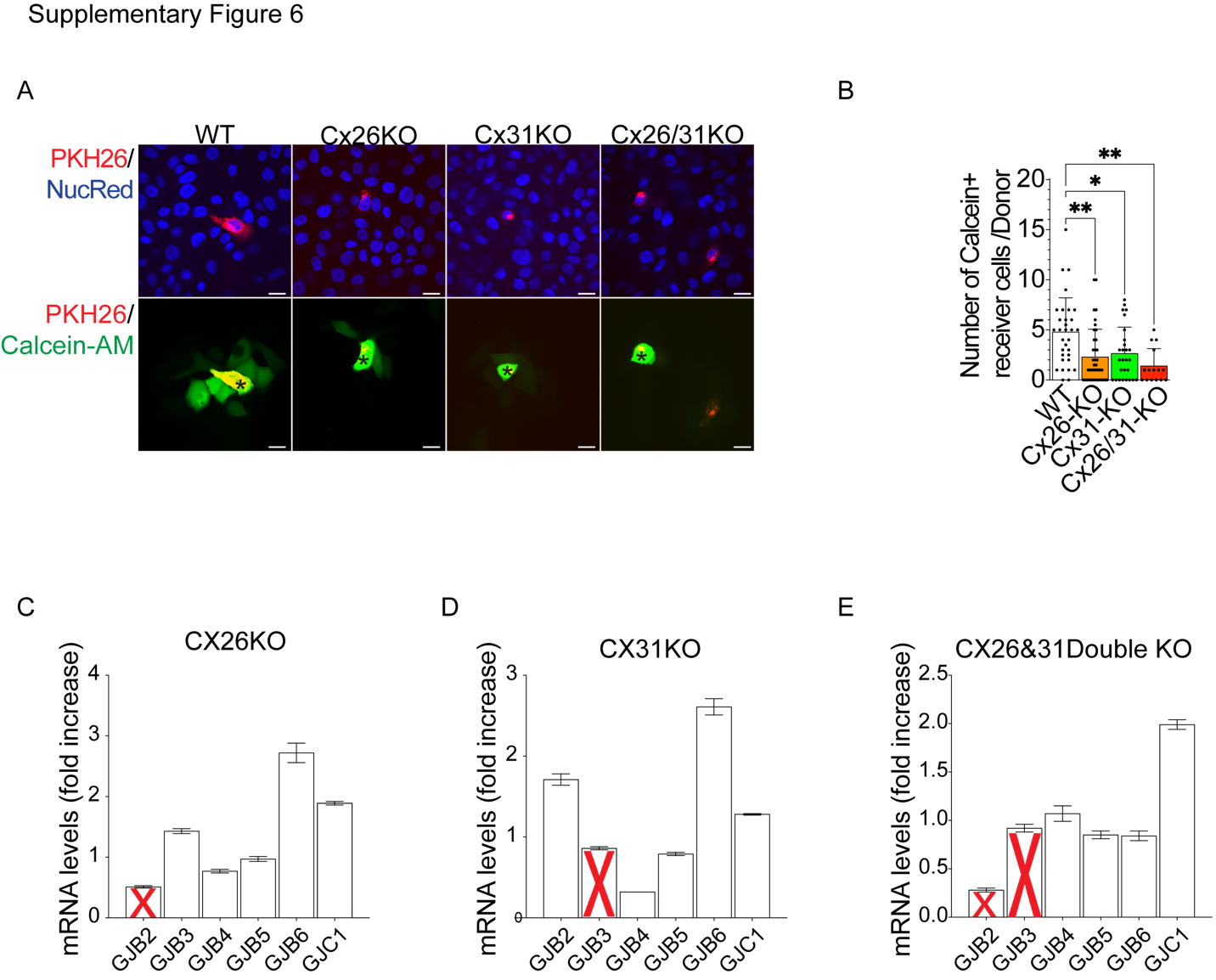


**Supplementary Figure 6. GJIC assays and Expression of mRNA levels of different connexins in HaCaT cells KO for Connexin 26, or Connexin 31 or double KO**

**A-B.** A) Donor cells were loaded with Calcein-AM and PKH26 (indicated by asterisks), then overlaid onto recipient cells followed by far-red NucLight labelling. Representative confocal images are shown for control, Cx26 CRISPR KO, and Cx31 CRISPR KO and double KO cells. Scale bar 20μm. B) Gap junctional transfer was quantified as Calcein spread into NucLight-positive, PKH26-negative recipient cells. Data are expressed as the mean ± SD (n = 15-35 fields of view from 3 independent experiments). *p = 0.028, **p = 0.0022 and 0.0014, one-way Anova followed by Bonferroni multiple comparison test.

**C-E**. Levels of mRNA of the indicated Connexins genes in HaCaT clones following CRISPR-mediated depletion of Connexin 26 (A, Cx26KO) or Connexin 31(B, Cx31KO) or both (C, Cx26/31KO) quantified by qRT-PCR. Data are the mRNA fold increase relative to the levels of control cells after normalizing for GAPDH and 18S mRNA levels (n = 3 replicates). Please note that although mRNA transcripts may still be detected by qRT-PCR, these clones are functionally null for Cx31 due to the generation of out-of-frame transcripts incapable of producing protein. Please note that although mRNA transcripts may still be detected in the KO clones (indicated by red crosses), this is an expected outcome due to the design of qRT-PCR probes targeting regions upstream of the CRISPR-induced frameshift mutations and premature stop codons. As a result, non-degraded mRNA fragments can be amplified despite the absence of functional protein production.


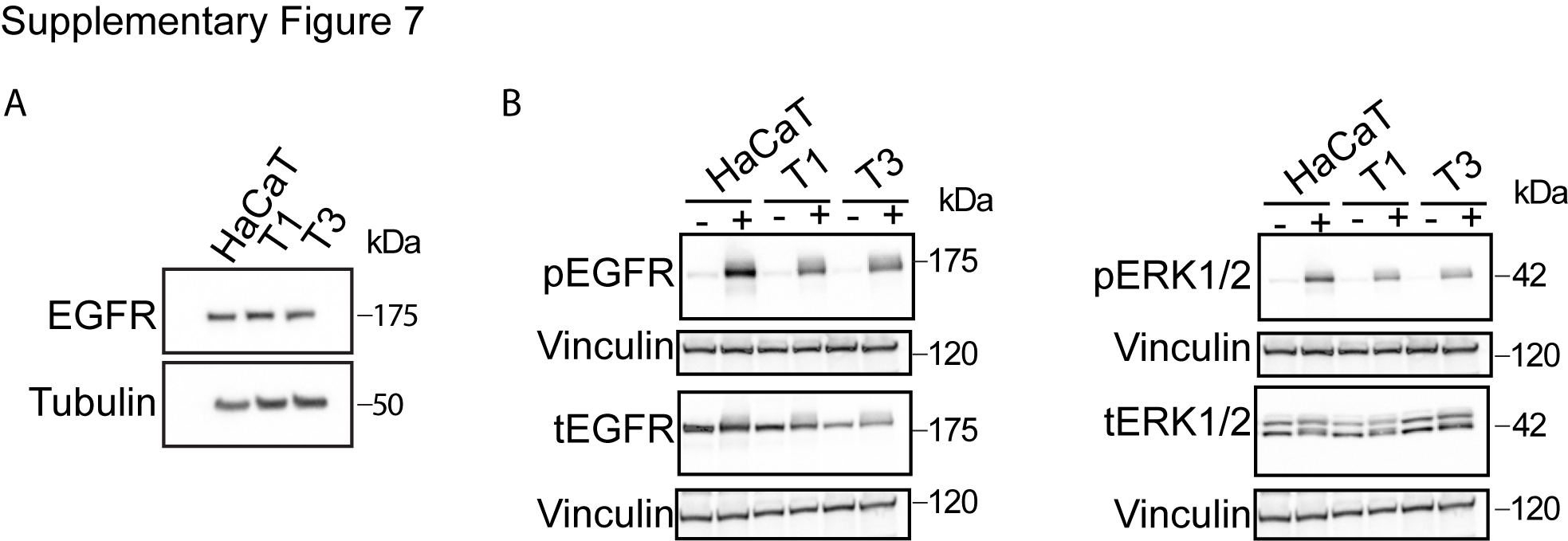


**Supplementary Figure 7. Expression levels of total and phosphorylated proteins of EGFR, and ERK1/2 in VFC T1 and T3 cells.**

**A.** Expression level measured by WB of EGFR in HaCaT, VFC T1 and T3 cells cultured in growing conditions. Tubulin and Vinculin were used as a loading control. Molecular weights are indicated on the right. (Representative WB of 3 independent experiments)

**B.** Expression and phosphorylation status of EGFR (left) and ERK1/2 (right) in HaCaT, VFC T1 and T3 cells, stimulated with EGF (100 ng/ml) for 5 min and analyzed by WB. Vinculin was used as a loading control. Molecular weights are indicated on the right. (Representative WB of 3 independent experiments).


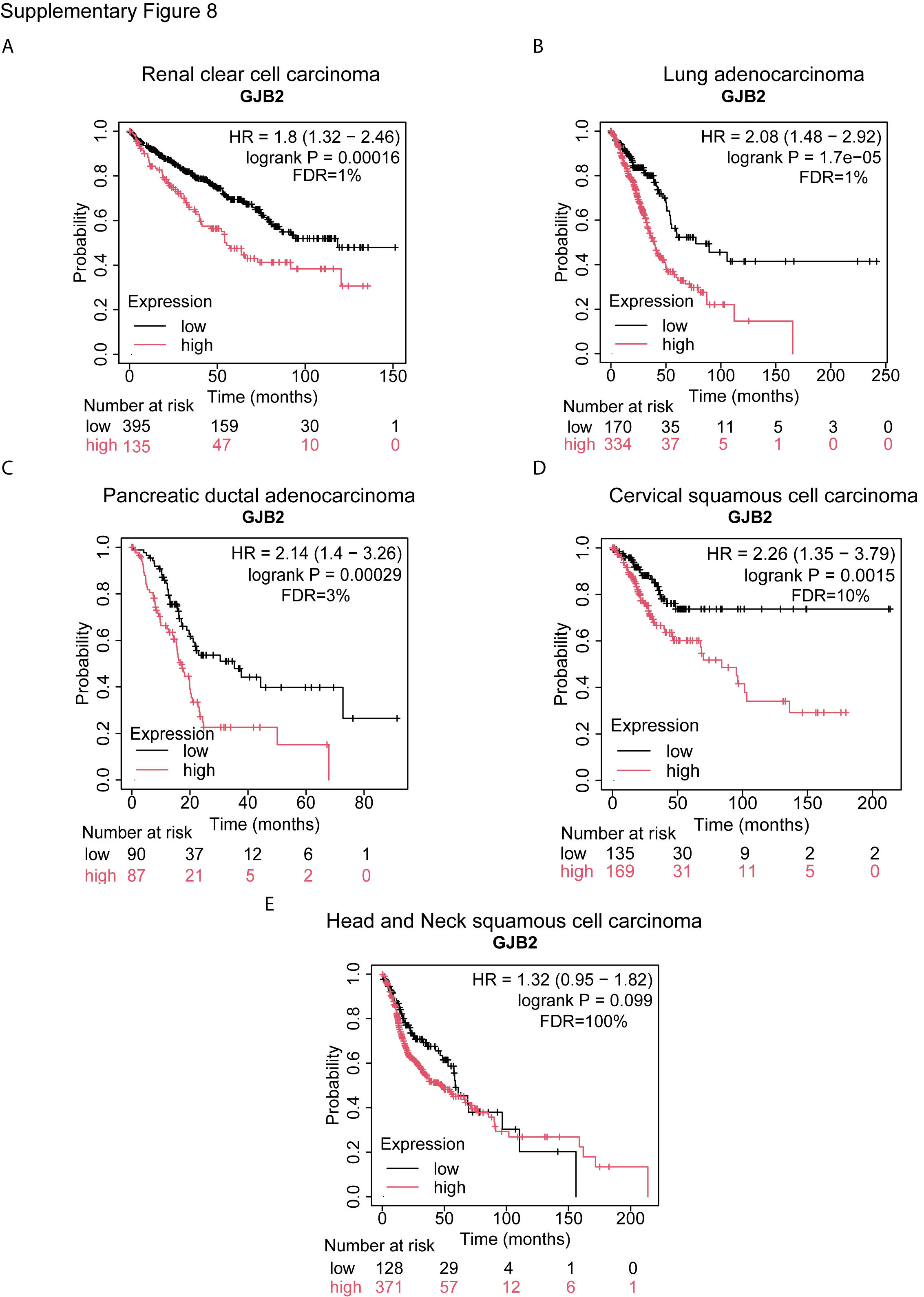


**Supplementary Figure 8. Elevated GJB2 expression is associated with poor overall survival in multiple tumor types.**

Kaplan-Meier survival curves show the correlation between GJB2 mRNA expression and overall survival (OS) in several human cancers. Patients were stratified into high and low GJB2 expression groups based on the optimal cutoff value identified between the lower and upper quartiles. Statistical analysis was performed using the log-rank (Mantel-Cox) test, and the hazard ratio (HR) with 95% confidence intervals (CI) is indicated for each tumor type.

Significant associations between high GJB2 expression and worse prognosis were observed in renal clear cell carcinoma (A), lung adenocarcinoma (B), and pancreatic ductal adenocarcinoma (C), with a false discovery rate (FDR) <5%. A trend toward significance was detected in cervical squamous cell carcinoma (D) (FDR = 10%). No statistically significant difference was observed in head and neck squamous cell carcinoma (E).

**Supplementary Movies**

**Movie 1 collective motility in HaCaT cells treated with different concentration of EGF**

Quiescent HaCaT-Fucci cells mock treated (CTRL) or treated with the indicated concentrations of EGF. The images were captured using phase-contrast microscopy at a frame rate of 50 fps (12h-48h). Scale bar 100µm.

**Movie 2 EGF-induced flocking motion is accompanied by entry into the cell cycle illustrated by the increase ratio of G2/M cells.**

Quiescent HaCaT-Fucci cells treated in the absence (CTRL) or the presence of the indicated concentrations of EGF. Nuclei of cells progressing from G0/G1 to G2/M become green. The images were captured using fluorescence microscopy (FITC, Cy3) at a frame rate of 50 fps (0h-45h50’). Scale bar 100µm.

**Movie 3 Effects of Rock inhibitor (Y2762) on the emergence of EGF-induced collective motility in HaCaT cells**

Quiescent HaCaT-Fucci cells treated or not with 100ng/ml EGF in the presence of Y2762 (15µM).

Upper movies: phase contrast; lower movies: fluorescence (FITC, Cy3). Control untreated cells (CTRL); 100 ng/ml EGF; 100 ng/ml EGF+Y27632(15µM). The images were captured using phase-contrast microscopy at a frame rate of 50 fps (0h-51h). Scale bar 100µm.

**Movie 4 Effects of CDK4/6 inhibitor (Palbociclib) on the emergence of EGF-induced flocking motility in HaCaT cells**

HaCaT-Fucci cells treated or not with 100ng/ml EGF in the presence of Palbociclib (100nM)

Upper movies: phase contrast; lower movies Fluorescence (FITC, Cy3). From left to right: Control untreated cells; 100 ng/ml EGF; 100 ng/ml EGF+ Palbociclib (100nM). The images were captured using phase-contrast microscopy at a frame rate of 50 fps. (0h-53h55’). Scale bar 100µm.

**Movie 5 Collective flocking motility in A431 cells induced by EGF**

Cells were treated with 100 ng/ml EGF or left untreated. The images were captured using phase-contrast microscopy at a frame rate of 50 fps (12h-48h). Scale bar, 100 µm

**Movie 6 Collective flocking motility in bronchial primary cultures at the air-liquid interface (ALI) induced by EGF.**

Left: untreated ALI cultures; right: ALI cultures treated with 100 ng/ml EGF. The images were captured using phase-contrast microscopy at a frame rate of 50 fps (0h-48h). Scale bar, 100 µm**.**

**Movie 7 Collective flocking motility in bronchial primary cultures at the air-liquid interface (ALI) induced by Amphiregulin.**

Left: untreated ALI cultures; right: ALI cultures treated with 100 ng/ml Amphiregulin (AREG). The images were captured using phase-contrast microscopy at a frame rate of 50 fps (0h-35h55’). Scale bar, 100 µm.

**Movie 8 Dynamin inhibition impairs EGF-induced flocking motion in HaCaT cells.**

Quiescent HaCaT cells were treated or not with 100 ng/ml EGF in the presence of the dynamin inhibitor, Dynasore (40 µM). From left to right: control untreated cells, 100 ng/ml EGF, and 100 ng/ml EGF + Dynasore (40 µM). The images were captured using phase-contrast microscopy at a frame rate of 50 fps (0h-48h). Scale bar, 100 µm.

**Movie 9 Silencing of Clathrin-dependent endocytosis inhibits EGF-induced collective flocking motility in HaCaT cells**

Quiescent HaCaT cells treated or not with 100 ng/ml EGF after silencing of Clathrin Heavy Chain (CLTHC). From left to right: Control HaCaT + EGF; CLTHC-KD HaCaT + EGF; HaCaT (Negative control siRNA) + EGF. The images were captured using phase-contrast microscopy at a frame rate of 50 fps (0h-48h). Scale bar , 100 µm.

**Movie 10 Impairment of Non Clathrin-dependent Endocytosis (NCE) inhibits EGF-induced collective flocking motility in HaCaT cells**

Quiescent HaCaT cells treated or not with 100 ng/ml EGF after silencing of Reticulon 3 (RTN3) to inhibit NCE and Reticulon 4 (RTN4), used as control. From left to right: Control HaCaT + EGF; RTN3-KD RTN4-KD + EGF. The images were captured using phase-contrast microscopy at a frame rate of 50 fps (0h-48h). Scale bar , 100 µm.

**Movie 12 Inhibition of MAPK pathway impairs collective flocking motility in HaCaT cells**

Quiescent HaCaT cells treated or not with 100 ng/ml EGF in the presence of the MEK inhibitor PD025901 (1 µM). From left to right: Control untreated cells; 100 ng/ml EGF; 100 ng/ml EGF + PD025901 (1 µM). The images were captured using phase-contrast microscopy at a frame rate of 50 fps (0h-54h). Scale bar, 100 µm

**Movie 13 Inhibition of AKT pathway impairs collective flocking motility in HaCaT cells** Quiescent HaCaT cells treated or not with 100 ng/ml EGF in the presence of the AKT inhibitor MK2206 (10 µM). From left to right: Control untreated cells; 100 ng/ml EGF; 100 ng/ml EGF + MK2206 (10 µM). The images were captured using phase-contrast microscopy at a frame rate of 50 fps (0h-53h55’). Scale bar, 100 µm.

**Movie 13 Stat3 inhibition has no effect on EGF-induced collective flocking motility in HaCaT cells**
Quiescent HaCaT cells treated or not with 100 ng/ml EGF in the presence of the STAT3 inhibitor, Stattic (5 µM). From left to right: Control untreated cells; 100 ng/ml EGF; 100 ng/ml EGF + Stattic (5 µM). The images were captured using phase-contrast microscopy at a frame rate of 50 fps (0h-48h). Scale bar, 100 µm.

**Movie 14 Inhibition of transcription impairs EGF-induced collective flocking motility in HaCaT cells**

Quiescent HaCaT cells treated or not with 100 ng/ml EGF in the presence of the inhibitor of transcriptionActinomycin D (5 µM). From left to right: Control untreated cells; 100 ng/ml EGF; 100 ng/ml EGF + Actinomycin D (5 µM). The images were captured using phase-contrast microscopy at a frame rate of 50 fps (0h-48h. Scale bar, 100 µm.

**Movie 15 Inhibition of transcription by DRB impairs EGF-induced collective flocking motility in HaCaT cells**

Quiescent HaCaT cells treated or not with 100 ng/ml EGF in the presence of the transcription inhibitor DRB (5 µM). From left to right: Control untreated cells; 100 ng/ml EGF; 100 ng/ml EGF + DRB (5 µM). The images were captured using phase-contrast microscopy at a frame rate of 50 fps (0h-51h). Scale bar, 100 µm.

**Movie 16 EGF-induced flocking motion promotes large shape changes in HaCaT-Ecad-GFP cells** Untreated HaCaT-Ecad-GFP cells serum deprived for 48h prior to recording (left); HaCaT-Ecad-GFP cells serum deprived for 48h and treated with 100 ng/ml EGF for 24 hours prior to recording (right). Images were acquired using a spinning disk confocal microscope. The E-cadherin signal (3 different Z planes spaced 3 µm apart) was recorded every 10 min over a 24h period using a 20x/0.75 objective, 30fps, (24h-36h). Scale bar, 100 µm.

**Movie 17 Cell 3D segmentation for cell volume estimation**

3D segmentation obtained with ARIVIS of quiescent HaCaT cells treated or not with 100 ng/ml EGF, stained with Phalloidin-FITC (Phalloidin) and DAPI (DNA). Images acquired using a Leica TCS SP8 laser confocal scanner mounted on a Leica DMi8 microscope with motorized stage (oil immersion 63× objective, x2.5 optical zoom, 0.3mm z-stack). Upper panel: Control untreated cells.
Lower panel: Cells treated with EGF for 30 hours.

**Movie 18 Analysis of density fluctuations during EGF-induced collective flocking motility**Segmentation of nuclei of untreated (left) and EGF-stimulated (right) HaCaT cells. The color bar represents the local cell density, scaling linearly from 0 to 0.006 µm^-^².

**Movie 19 Inhibition of gap junctions impairs EGF-induced collective flocking motility in HaCaT cells**

Quiescent HaCaT cells treated or not with 100 ng/ml EGF in the presence of the gap junction blocker Carbenoxolone (CBX, 16 µM). From left to right: 100 ng/ml EGF; 100 ng/ml EGF + CBX (16 µM The images were captured using phase-contrast microscopy at a frame rate of 50 fps (0h-48h). Scale bar, 100 µm.

**Movie 20 Inhibition of Gap Junctions impairs EGF-induced collective flocking motility in HaCaT cells**Quiescent HaCaT cells treated or not with 100 ng/ml EGF in the presence of the gap junction blocker 6-dichloro-1-β-D-ribofuranosylbenzimidazole (DRB, 50 µM). From left to right: 100 ng/ml EGF; 100 ng/ml EGF + DRB (50 µM). The images were captured using phase-contrast microscopy at a frame rate of 50 fps (0h-48h). Scale bar, 100 µm.

**Movie 21 Inhibition of Gap Junctions impairs Amphiregulin-induced collective flocking motility in Bronchial Aire-Liquid Interface (ALI) cultures**

ALI cultures of human primary bronchial epithelial tissue explant treated or not with 100 ng/ml Amphiregulin (AREG) in the presence of the gap junction blocker Carbenoxolone (CBX, 50 µM).
From left to right: AREG (100 ng/ml); AREG (100 ng/ml) + CBX (50 µM). The images were captured using phase-contrast microscopy at a frame rate of 50 fps (0h-48h). Scale bar, 100 µm.

**Movie 22 Activation of Gap Junctions enhances EGF-induced collective flocking motility in HaCaT cells**Quiescent HaCaT cells treated or not with 100 ng/ml EGF in the presence of the gap junction activator Valproic Acid (VA, 1 mM). From left to right: 100 ng/ml EGF; 100 ng/ml EGF + VA (1 mM). The images were captured using phase-contrast microscopy at a frame rate of 50 fps (0h-39h). Scale bar, 100 µm.

**Movie 23 EGF enhances intercellular dye transfer in HaCaT cells**

Fluorescence recovery after photobleaching (FRAP) in HaCaT cells loaded with calcein-AM (20 µM) for 45 min. A specific area (95 µm diameter) of the monolayer is bleached using the FRAP module with a 405 nm laser at maximum power, and the recovery of calcein-AM signal is monitored for 30 minutes post-bleaching, reflecting intercellular dye transfer. From left to right: Control untreated cells; EGF-treated cells (100 ng/ml) for 24 hours. Images were recorded using a Confocal Spinning Disk microscope (Olympus) equipped with IX83 inverted microscope, 20x/0.75 objective, captured at 10 fps. Scale bar, 50 µm.

**Movie 24 Intercellular dye transfer enhanced by EGF is prevented by Gap junction blocker Carbenoxolone (CBX)**

Fluorescence recovery after photobleaching (FRAP) in HaCaT cells loaded with calcein-AM (20 µM) for 45 min. A specific area (95 µm diameter) of the monolayer is bleached using the FRAP module with a 405 nm laser at maximum power, and the recovery of calcein-AM signal is monitored for 30 minutes post-bleaching. From left to right: EGF-treated cells (100 ng/ml) for 24 hours; EGF (100 ng/ml) + CBX (16 µM)-treated cells for 24 hours. Images were recorded using a Confocal Spinning Disk microscope (Olympus) equipped with IX83 inverted microscope, 20x/0.75 objective, captured at 10 fps. Scale bar, 50 µm.

**Movie 25 Gap junction blocker Carbenoxolone prevents the active wetting of HaCaT spheroids enhanced by EGF.**

Active wetting of FN-coated substrate by HaCaT spheroids over 30 hours. From left to right: HaCaT spheroids treated with EGF (100 ng/ml); HaCaT spheroids treated with EGF (100 ng/ml) + CBX (100 µM). 4x/0.13 objective, the images were captured using brightfield microscopy at a frame rate 50 fps (0h-30h). Scale bar, 200 µm.

**Movie 26 Silencing of Connexin 26 and/or Connexin 31 impairs collective flocking motility in HaCaT cells.**
Quiescent wild-type HaCaT cells or Connexin-depleted clones were serum deprived for 48h and treated with 100 ng/ml EGF. From left to right: HaCaT WT + EGF; HaCaT Cx26KO + EGF; HaCaT Cx31KO + EGF; HaCaT Cx26-31KO + EGF. The images were captured using phase-contrast microscopy at a frame rate of 50 fps (0h-49h55’). Scale bar, 100 µm.

**Movie 27 EGF stimulation induces robust and rapid collective flocking motility in Vocal Fold Cancer (VFC) T1 cells.**

Serum starved VFC T1 cells treated or not with 100 ng/ml EGF. From left to right: Control (CTRL) untreated cells; 100 ng/ml EGF. The images were captured using phase-contrast microscopy at a frame rate of 50 fps (0h-48h). Scale bar, 100 µm.

**Movie 28 EGF stimulation induces robust and rapid collective flocking motility in Vocal Fold Cancer (VFC) T3 cells**

Serum starved VFC T3 cells treated or not with 100ng/ml EGF. From left to right: Control untreated cells and EGF (100 ng/ml). The images were captured using phase-contrast microscopy at a frame rate of 50 fps (0h-48h). Scale bar 100µm.

**Movie 29 Analysis of Neighbour exchanges in HaCaT, VFC T1 and T3 cells during EGF-induced flocking motion.**

a probability density distribution that illustrates the likelihood of finding a particular tagged cell at a position (x, y) relative to a reference cell after a given time delay. Initially, at δt = 0, the tagged cell was a neighbor of the reference cell. The distribution, denoted as P(r, δt), is mathematically defined as: $\boldsymbol{P}\left( \boldsymbol{r}\mathbf{,}\boldsymbol{\delta t} \right)\mathbf{=}\left\langle\left\langle\sum_{\boldsymbol{i}}^{\boldsymbol{N}\left( \boldsymbol{t} \right)} \sum_{\boldsymbol{j}}^{\boldsymbol{n}_{\boldsymbol{i}}\left( \boldsymbol{t} \right)} \boldsymbol{\Delta}\left( \boldsymbol{r}_{\boldsymbol{ij}}\left( \boldsymbol{t+\delta t} \right)\boldsymbol{-}\boldsymbol{r}_{\boldsymbol{ij}}\left( \boldsymbol{t} \right) \right) \right\rangle_{\boldsymbol{r}_{\boldsymbol{ij}}} \right\rangle_{\boldsymbol{t}}$, where δt is time delay, N(t) is the total number of cells in the field of view at t, ni(t) is the number of neighbors of cell i at t, rij(t) is the distance between cell i and cell j at time t. The panels from left to right show data for three different cell types: HaCaT cells, VFC T1 and VFC T3 cells.

**Movie 30 The gap junction blocker Carbenoxolone (CBX) inhibits EGF-induced collective flocking motility in Vocal Fold Cancer (VFC) T1**

Serum-starved VFC T1 cells were exposed to 100 ng/ml EGF either alone or in combination with CBX (125 µM). The left panel shows cells treated with EGF (100 ng/ml) alone. The right panel shows cells treated with both EGF (100 ng/ml) and CBX (125 µM). The images were captured using phase-contrast microscopy at a frame rate of 50 fps. Scale bar, 100 µm.

**Movie 31 The gap junction blocker Carbenoxolone (CBX) inhibits EGF-induced collective flocking motility in Vocal Fold Cancer (VFC) T3 cells**

Serum-starved VFC T3 cells were exposed to 100 ng/ml EGF either alone or in combination with CBX (125 µM). The left panel shows cells treated with EGF (100 ng/ml) alone. The right panel shows cells treated with both EGF (100 ng/ml) and CBX (125 µM). The images were captured using phase-contrast microscopy at a frame rate of 50 fps (0h-48h). Scale bar, 100 µm.

**Movie 32** **The** **Gap junction blocker Carbenoxolone impairs EGF-induced active wetting of VFC T1 spheroids**

This movie depicts the active wetting behavior of VFC T1 spheroids on a fibronectin (FN)-coated substrate over a 30-hour period: The top panel shows untreated spheroids; the middle panel shows spheroids treated with EGF (100 ng/ml); the bottom panel shows spheroids treated with both EGF (100 ng/ml) and CBX (150 mM). Images were captured using a 4x/0.13 objective at 50 fps (0h-30h). Scale bar 500µm

**Movie 33 The Gap junction blocker Carbenoxolone impairs EGF-induced active wetting of VFC T3 spheroids**

This movie depicts the active wetting behavior of VFC T3 spheroids on a fibronectin (FN)-coated substrate over a 30-hour period: The top panel shows untreated spheroids; the middle panel shows spheroids treated with EGF (100 ng/ml); the bottom panel shows spheroids treated with both EGF (100 ng/ml) and CBX (150 mM). Images were captured using a 4x/0.13 objective at 50 fps (0h-30h). Scale bar 500µm

**Tables**

**Table 1 Statistical tests performed and significance**

The following table indicates for each of the experiments in the various figures the statistical tests that have been used to evaluate significance

**Table 2 Connexins Knockout clones selected following Crispr-Cas9 gene editing.**

Table summarizing the selection and validation of CRISPR/Cas9-generated knockout clones targeting different connexin genes (GJB2, GJB3, GJB6, GJA1) in VFC cells. Each row reports the targeted gene, the genotype efficiency (ICE indel %), KO-Score (predicted knockout efficiency), and the R² value (goodness of fit for the ICE analysis). The "Indels" column specifies the types of insertions/deletions identified. Double knockout clones (targeting two connexins simultaneously) are also indicated. Target sequences and corresponding exons used for CRISPR editing are listed at the bottom. Clones were selected based on high indel frequency, high KO-score, and confirmed disruption of the target loci. Clones harboring frameshift mutations predicted to abrogate protein production were selected for further experiments. Although mRNA transcripts may still be detected by qRT-PCR, these clones are functionally null for Cx31 due to the generation of out-of-frame transcripts incapable of producing protein.

**References**

1. Oswald, L., et al., *Jamming transitions in cancer.* J Phys D Appl Phys, 2017. **50**(48): p. 483001.

2. Cheung, K.J. and A.J. Ewald, *A collective route to metastasis: Seeding by tumor cell clusters.* Science, 2016. **352**(6282): p. 167-9.

3. Palamidessi, A., et al., *Unjamming overcomes kinetic and proliferation arrest in terminally differentiated cells and promotes collective motility of carcinoma.* Nat Mater, 2019. **18**(11): p. 1252-1263.

4. Giavazzi, F., et al., *Flocking transitions in confluent tissues.* Soft Matter, 2018. **14**(18): p. 3471-3477.

5. Mitchel, J.A., et al., *In primary airway epithelial cells, the unjamming transition is distinct from the epithelial-to-mesenchymal transition.* Nat Commun, 2020. **11**(1): p. 5053.

6. Friedl, P. and D. Gilmour, *Collective cell migration in morphogenesis, regeneration and cancer.* Nat Rev Mol Cell Biol, 2009. **10**(7): p. 445-57.

7. Mayor, R. and S. Etienne-Manneville, *The front and rear of collective cell migration.* Nat Rev Mol Cell Biol, 2016. **17**(2): p. 97-109.

8. Lawson-Keister, E. and M.L. Manning, *Jamming and arrest of cell motion in biological tissues.* Curr Opin Cell Biol, 2021. **72**: p. 146-155.

9. Zehnder, S.M., et al., *Cell Volume Fluctuations in MDCK Monolayers.* Biophys J, 2015. **108**(2): p. 247-50.

10. Zehnder, S.M., et al., *Multicellular density fluctuations in epithelial monolayers.* Phys Rev E Stat Nonlin Soft Matter Phys, 2015. **92**(3): p. 032729.

11. Wei, C.J., X. Xu, and C.W. Lo, *Connexins and cell signaling in development and disease.* Annu Rev Cell Dev Biol, 2004. **20**: p. 811-38.

12. Yeager, M. and A.L. Harris, *Gap junction channel structure in the early 21st century: facts and fantasies.* Curr Opin Cell Biol, 2007. **19**(5): p. 521-8.

13. Zhou, M., et al., *The roles of connexins and gap junctions in the progression of cancer.* Cell Commun Signal, 2023. **21**(1): p. 8.

14. Maeda, E., et al., *Enhanced gap junction intercellular communication inhibits catabolic and pro-inflammatory responses in tenocytes against heat stress.* J Cell Commun Signal, 2017. **11**(4): p. 369-380.

15. Wong, P., et al., *The Role of Connexins in Wound Healing and Repair: Novel Therapeutic Approaches.* Front Physiol, 2016. **7**: p. 596.

16. Aasen, T., et al., *Connexins in cancer: bridging the gap to the clinic.* Oncogene, 2019. **38**(23): p. 4429-4451.

17. Marins, M., et al., *Gap junctions are involved in cell migration in the early postnatal subventricular zone.* Dev Neurobiol, 2009. **69**(11): p. 715-30.

18. Hudson, L., et al., *Dominant effect of gap junction communication in wound-induced calcium-wave, NFAT activation and wound closure in keratinocytes.* J Cell Physiol, 2021. **236**(12): p. 8171-8183.

19. Polusani, S.R., et al., *Cell coupling mediated by connexin 26 selectively contributes to reduced adhesivity and increased migration.* J Cell Sci, 2016. **129**(23): p. 4399-4410.

20. Wright, C.S., et al., *Connexin mimetic peptides improve cell migration rates of human epidermal keratinocytes and dermal fibroblasts in vitro.* Wound Repair Regen, 2009. **17**(2): p. 240-9.

21. Sharma, M., et al., *Connexin 43 mediated collective cell migration is independent of Golgi orientation.* Biol Open, 2023. **12**(10).

22. Khalil, A.A., et al., *Collective invasion induced by an autocrine purinergic loop through connexin-43 hemichannels.* J Cell Biol, 2020. **219**(10).

23. Lorusso, G., et al., *Connexins orchestrate progression of breast cancer metastasis to the brain by promoting FAK activation.* Sci Transl Med, 2022. **14**(661): p. eaax8933.

24. Zhu, Y., *Gap Junction-Dependent and -Independent Functions of Connexin43 in Biology.* Biology (Basel), 2022. **11**(2).

25. Langlois, S., et al., *The tumor-suppressive function of Connexin43 in keratinocytes is mediated in part via interaction with caveolin-1.* Cancer Res, 2010. **70**(10): p. 4222-32.

26. Kandouz, M. and G. Batist, *Gap junctions and connexins as therapeutic targets in cancer.* Expert Opin Ther Targets, 2010. **14**(7): p. 681-92.

27. Stoletov, K., et al., *Role of connexins in metastatic breast cancer and melanoma brain colonization.* J Cell Sci, 2013. **126**(Pt 4): p. 904-13.

28. Teleki, I., et al., *Correlations of differentially expressed gap junction connexins Cx26, Cx30, Cx32, Cx43 and Cx46 with breast cancer progression and prognosis.* PLoS One, 2014. **9**(11): p. e112541.

29. Lang, E., et al., *Coordinated collective migration and asymmetric cell division in confluent human keratinocytes without wounding.* Nat Commun, 2018. **9**(1): p. 3665.

30. Dupont, S., et al., *Role of YAP/TAZ in mechanotransduction.* Nature, 2011. **474**(7350): p. 179-83.

31. Zhao, T. and H. Yuan, *The analytical solution to the migration of an epithelial monolayer with a circular spreading front and its implications in the gap closure process.* Biomech Model Mechanobiol, 2023. **22**(4): p. 1349-1363.

32. Weston, W.A. and A.R. Barr, *A cell cycle centric view of tumour dormancy.* Br J Cancer, 2023. **129**(10): p. 1535-1545.

33. Zhang, Z., et al., *Endothelial cell-secreted EGF induces epithelial to mesenchymal transition and endows head and neck cancer cells with stem-like phenotype.* Cancer Res, 2014. **74**(10): p. 2869-81.

34. Schultz, P., *Vocal fold cancer.* Eur Ann Otorhinolaryngol Head Neck Dis, 2011. **128**(6): p. 301-8.

35. Kaivola, J., et al., *Restoring mechanophenotype reverts malignant properties of ECM-enriched vocal fold cancer.* bioRxiv, 2024: p. 2024.08.22.609159.

36. Sakaue-Sawano, A., et al., *Visualizing spatiotemporal dynamics of multicellular cell-cycle progression.* Cell, 2008. **132**(3): p. 487-98.

37. Costa, G., et al., *Asymmetric division coordinates collective cell migration in angiogenesis.* Nat Cell Biol, 2016. **18**(12): p. 1292-1301.

38. Petridou, N.I., et al., *Fluidization-mediated tissue spreading by mitotic cell rounding and non-canonical Wnt signalling.* Nat Cell Biol, 2019. **21**(2): p. 169-178.

39. Gupta, V.K. and O. Chaudhuri, *Mechanical regulation of cell-cycle progression and division.* Trends Cell Biol, 2022. **32**(9): p. 773-785.

40. Krupp, M.N., D.T. Connolly, and M.D. Lane, *Synthesis, turnover, and down-regulation of epidermal growth factor receptors in human A431 epidermoid carcinoma cells and skin fibroblasts.* J Biol Chem, 1982. **257**(19): p. 11489-96.

41. Spurlin, J.W., et al., *Mesenchymal proteases and tissue fluidity remodel the extracellular matrix during airway epithelial branching in the embryonic avian lung.* Development, 2019. **146**(16).

42. Park, J.A., et al., *Compressive Stress Causes an Unjamming Transition and an Epithelial-Mesenchymal Transition in the Airway Epithelium in Asthma.* Ann Am Thorac Soc, 2016. **13 Suppl 1**(Suppl 1): p. S102.

43. O'Sullivan, M.J., et al., *Irradiation Induces Epithelial Cell Unjamming.* Front Cell Dev Biol, 2020. **8**: p. 21.

44. Park, J.A., et al., *Unjamming and cell shape in the asthmatic airway epithelium.* Nat Mater, 2015. **14**(10): p. 1040-8.

45. Park, J.A., et al., *Collective migration and cell jamming in asthma, cancer and development.* J Cell Sci, 2016. **129**(18): p. 3375-83.

46. Stancil, I.T., et al., *Pulmonary fibrosis distal airway epithelia are dynamically and structurally dysfunctional.* Nat Commun, 2021. **12**(1): p. 4566.

47. Stern, K.A., T.L. Place, and N.L. Lill, *EGF and amphiregulin differentially regulate Cbl recruitment to endosomes and EGF receptor fate.* Biochem J, 2008. **410**(3): p. 585-94.

48. Shen, Y., et al., *Scale-free flocking and giant fluctuations in epithelial active solids.* bioRxiv, 2024: p. 2024.10.18.619133.

49. Malinverno, C., et al., *Endocytic reawakening of motility in jammed epithelia.* Nat Mater, 2017. **16**(5): p. 587-596.

50. Caldieri, G., et al., *Reticulon 3-dependent ER-PM contact sites control EGFR nonclathrin endocytosis.* Science, 2017. **356**(6338): p. 617-624.

51. Sigismund, S., et al., *Threshold-controlled ubiquitination of the EGFR directs receptor fate.* EMBO J, 2013. **32**(15): p. 2140-57.

52. Wee, P. and Z. Wang, *Epidermal Growth Factor Receptor Cell Proliferation Signaling Pathways.* Cancers (Basel), 2017. **9**(5).

53. Fei, J., et al., *Transfer RNA-mediated regulation of ribosome dynamics during protein synthesis.* Nat Struct Mol Biol, 2011. **18**(9): p. 1043-51.

54. Krumm, A., L.B. Hickey, and M. Groudine, *Promoter-proximal pausing of RNA polymerase II defines a general rate-limiting step after transcription initiation.* Genes Dev, 1995. **9**(5): p. 559-72.

55. Garcia-Rodriguez, C., et al., *The antiseizure medication valproate increases hemichannel activity found in brain cells, which could worsen disease outcomes.* J Neurochem, 2024. **168**(6): p. 1045-1059.

56. Perez-Gonzalez, C., et al., *Active wetting of epithelial tissues.* Nat Phys, 2019. **15**(1): p. 79-88.

57. Sackmann, E. and R.F. Bruinsma, *Cell adhesion as wetting transition?* Chemphyschem, 2002. **3**(3): p. 262-9.

58. Douezan, S. and F. Brochard-Wyart, *Dewetting of cellular monolayers.* Eur Phys J E Soft Matter, 2012. **35**(5): p. 34.

59. Style, R.W., et al., *Surface tension and contact with soft elastic solids.* Nat Commun, 2013. **4**: p. 2728.

60. Mehran, R., G.D. Dangas, and S.D. Weisbord, *Contrast-Associated Acute Kidney Injury.* N Engl J Med, 2019. **380**(22): p. 2146-2155.

61. Mettang, M., et al., *Blocking distinct interactions between Glioblastoma cells and their tissue microenvironment: A novel multi-targeted therapeutic approach.* Sci Rep, 2018. **8**(1): p. 5527.

62. Gyorffy, B., *Integrated analysis of public datasets for the discovery and validation of survival-associated genes in solid tumors.* Innovation (Camb), 2024. **5**(3): p. 100625.

63. Bi, D., et al., *Motility-driven glass and jamming transitions in biological tissues.* Phys Rev X, 2016. **6**(2).

64. Pinheiro, D. and J. Mitchel, *Pulling the strings on solid-to-liquid phase transitions in cell collectives.* Curr Opin Cell Biol, 2024. **86**: p. 102310.

65. Garcia, S., et al., *Physics of active jamming during collective cellular motion in a monolayer.* Proc Natl Acad Sci U S A, 2015. **112**(50): p. 15314-9.

66. Pajic-Lijakovic, I. and M. Milivojevic, *Cell jamming-to-unjamming transitions and vice versa in development: Physical aspects.* Biosystems, 2023. **234**: p. 105045.

67. Tschumperlin, D.J., et al., *Mechanotransduction through growth-factor shedding into the extracellular space.* Nature, 2004. **429**(6987): p. 83-6.

68. Chu, E.K., et al., *Bronchial epithelial compression regulates epidermal growth factor receptor family ligand expression in an autocrine manner.* Am J Respir Cell Mol Biol, 2005. **32**(5): p. 373-80.

69. Stancil, I.T., et al., *Interleukin-6-dependent epithelial fluidization initiates fibrotic lung remodeling.* Sci Transl Med, 2022. **14**(654): p. eabo5254.

70. Lang, E., et al., *Mechanical coupling of supracellular stress amplification and tissue fluidization during exit from quiescence.* Proc Natl Acad Sci U S A, 2022. **119**(32): p. e2201328119.

71. Lang, E., et al., *Topology-guided polar ordering of collective cell migration.* Sci Adv, 2024. **10**(16): p. eadk4825.

72. Kotini, M., et al., *Gap junction protein Connexin-43 is a direct transcriptional regulator of N-cadherin in vivo.* Nat Commun, 2018. **9**(1): p. 3846.

73. Brinkman, E.K. and B. van Steensel, *Rapid Quantitative Evaluation of CRISPR Genome Editing by TIDE and TIDER.* Methods Mol Biol, 2019. **1961**: p. 29-44.

74. Patro, R., et al., *Salmon provides fast and bias-aware quantification of transcript expression.* Nat Methods, 2017. **14**(4): p. 417-419.

75. Dobin, A., et al., *STAR: ultrafast universal RNA-seq aligner.* Bioinformatics, 2013. **29**(1): p. 15-21.

76. Tinevez, J.Y., et al., *TrackMate: An open and extensible platform for single-particle tracking.* Methods, 2017. **115**: p. 80-90.

77. Stringer, C., et al., *Cellpose: a generalist algorithm for cellular segmentation.* Nat Methods, 2021. **18**(1): p. 100-106.

78. Schindelin, J., et al., *Fiji: an open-source platform for biological-image analysis.* Nat Methods, 2012. **9**(7): p. 676-82.

79. Legland, D., I. Arganda-Carreras, and P. Andrey, *MorphoLibJ: integrated library and plugins for mathematical morphology with ImageJ.* Bioinformatics, 2016. **32**(22): p. 3532-3534.

80. Miura, K. *Analysis of FRAP Curves* 2010; Available from: <http://wiki.cmci.info/dls/FRAPmanual.htm>.

81. Warawdekar, U.M., *An Assay to Assess Gap Junction Communication in Cell Lines.* J Biomol Tech, 2019. **30**(1): p. 1-6.

82. Beznoussenko, G.V. and A.A. Mironov, *Correlative video-light-electron microscopy of mobile organelles.* Methods Mol Biol, 2015. **1270**: p. 321-46.

83. Lord, S.J., et al., *SuperPlots: Communicating reproducibility and variability in cell biology.* J Cell Biol, 2020. **219**(6).
